# Distance-dependent inhibition of translation initiation by downstream out-of-frame AUGs is consistent with a Brownian ratchet process of ribosome scanning

**DOI:** 10.1186/s13059-022-02829-1

**Published:** 2022-12-12

**Authors:** Ke Li, Jinhui Kong, Shuo Zhang, Tong Zhao, Wenfeng Qian

**Affiliations:** 1 State Key Laboratory of Plant Genomics, Institute of Genetics and Developmental Biology, Innovation Academy for Seed Design, Chinese Academy of Sciences, Beijing, 100101 China; 2 University of Chinese Academy of Sciences, Beijing, 100049 China; 3 Institute of Microbiology, Chinese Academy of Sciences, Beijing, 100101 China

**Keywords:** Translation initiation, Leaky scanning, Downstream AUG, Strictly unidirectional scanning model, Brownian ratchet scanning model, Preinitiation complex, Protein homeostasis

## Abstract

**Background:**

Eukaryotic ribosomes are widely presumed to scan mRNA for the AUG codon to initiate translation in a strictly 5′–3′ movement (i.e., strictly unidirectional scanning model), so that ribosomes initiate translation exclusively at the 5′ proximal AUG codon (i.e., the first-AUG rule).

**Results:**

We generate 13,437 yeast variants, each with an ATG triplet placed downstream (dATGs) of the annotated ATG (aATG) codon of a green fluorescent protein. We find that out-of-frame dATGs can inhibit translation at the aATG, but with diminishing strength over increasing distance between aATG and dATG, undetectable beyond ~17 nt. This phenomenon is best explained by a Brownian ratchet mechanism of ribosome scanning, in which the ribosome uses small-amplitude 5′–3′ and 3′–5′ oscillations with a net 5′–3′ movement to scan the AUG codon, thereby leading to competition for translation initiation between aAUG and a proximal dAUG. This scanning model further predicts that the inhibitory effect induced by an out-of-frame upstream AUG triplet (uAUG) will diminish as uAUG approaches aAUG, which is indeed observed among the 15,586 uATG variants generated in this study. Computational simulations suggest that each triplet is scanned back and forth approximately ten times until the ribosome eventually migrates to downstream regions. Moreover, this scanning process could constrain the evolution of sequences downstream of the aATG to minimize proximal out-of-frame dATG triplets in yeast and humans.

**Conclusions:**

Collectively, our findings uncover the basic process by which eukaryotic ribosomes scan for initiation codons, and how this process could shape eukaryotic genome evolution.

**Supplementary Information:**

The online version contains supplementary material available at 10.1186/s13059-022-02829-1.

## Introduction

To synthesize functional proteins and maintain protein homeostasis, the genetic information carried by messenger RNAs (mRNAs) must be faithfully transmitted to proteins [1]. In particular, the recognition of the initiation codon of the canonical open reading frame (ORF) by ribosomes is crucial to obtaining functional proteins. While it is well-established that most translation starts at the AUG codon [2, 3], AUG triplets can occur with an approximate frequency of every 4^3^ nucleotides (i.e., 64 nt), presenting a serious challenge for efficient ribosomal recognition of the AUG codon corresponding to the canonical ORF in a given mRNA.

Eukaryotic cells are known to tackle the challenge by using a “scanning” mechanism [4, 5] based on the 43S preinitiation complex (PIC), comprised of a 40S ribosomal subunit, several eukaryotic initiation factors (eIFs), methionyl initiator transfer RNA (Met-tRNAi), and guanosine triphosphate [6–10]. PIC scanning starts with attachment to the 5′-cap of an mRNA, after which the PIC migrates along the mRNA 1 nt at a time searching for the AUG codon: successive triplets enter the P-site of the 40S ribosomal subunit, where they are inspected for complementarity to the Met-tRNAi anticodon [4, 5, 11–13].

According to current understanding, the PIC remains tethered to the eukaryotic mRNA (without jumping) during scanning [5, 14]. This working model is supported by evidence showing that the most upstream (i.e., 5′) AUG triplet is preferentially used as the primary initiation codon [11, 15, 16]; insertion of an additional AUG triplet upstream (uAUG) of the annotated AUG triplet (aAUG, the initiation codon of the canonical ORF) can prevent translation initiation at the aAUG [15, 17–22]. Further, the insertion of a strong mRNA secondary structure between the 5′-cap and the aAUG can also prevent translation initiation [23, 24].

**Fig. 1:**
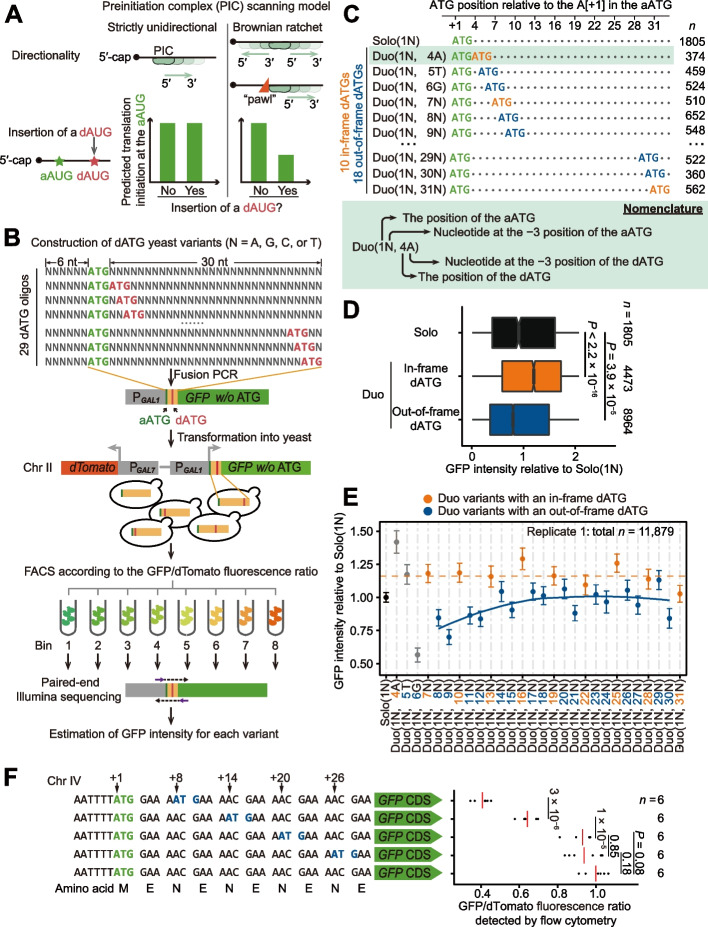
Testing PIC scanning models using thousands of dATG variants. **A** Predictions of the strictly unidirectional model and the Brownian ratchet scanning model on translational efficiency when an AUG is inserted downstream of the aAUG. **B** High-throughput construction of dATG variants with doped nucleotides and detection of GFP intensity en masse via FACS-seq. **C** Nomenclature of Solo and Duo variants. The aATGs, in-frame dATGs, and out-of-frame dATGs are shown in green, orange, and blue, respectively. Each dot in the sequences represents a nucleotide that cannot form an ATG triplet or an in-frame stop codon. **D** Boxplot shows GFP intensities (normalized by dTomato intensity, here and elsewhere in this study when presenting FACS-seq data) for Solo and Duo variants. *P* values were given by the Mann-Whitney *U* tests. **E** The average GFP intensities (dots) and the 95% confidence intervals (error bars) of Duo variants. The orange dashed line represents the average GFP intensity of all Duo variants with an in-frame dATG, and the blue curve represents the local regression line generated by *R* function “geom_smooth” (span = 1) for the Duo variants with an out-of-frame dATG. Duo variants with the dATG at positions from +4 to +6 (shown in gray) had fixed nucleotides at the −3 position of the dATG (due to the aATG), and therefore, were not used to fit the local regression line. **F** A small-scale experiment that introduced dATGs by synonymous mutations and strictly controlled the flanking sequences of the aATG and dATG. The GFP/dTomato fluorescence ratio estimated for each replicate is shown in a dot and the average value is shown in the red line. The GFP/dTomato ratios were normalized to the variant lacking additional dATG, which were 0.41, 0.64, 0.93, and 0.94 for the variants with dATGs inserted at the +8, +14, +20, and +26 positions, respectively. *P* values were given by *t*-tests

Since the PIC starts scanning at the 5′-cap of an mRNA and the aAUG codon is somewhere downstream (i.e., 3′), PIC scanning results in a net 5′–3′ ribosomal movement [6, 25]. Currently, there are two competing models to explain the directionality of individual scanning steps (i.e., 1 nt each step). The more established model that is commonly described in textbooks is the strictly unidirectional scanning model [2, 3, 15, 16], which posits that the PIC scans exclusively in the 5′–3′ direction (Fig. 1A) possibly governed by an RNA helicase constantly fed by adenosine triphosphate (ATP). Sometimes the PIC misses an AUG triplet, an event termed “leaky scanning,” and will continue to scan the mRNA further downstream, which can enable access to downstream AUGs (dAUG) by the PIC. The scanning process proceeds until an AUG triplet is recognized [12, 14, 15].

By contrast, an alternative model—the Brownian ratchet scanning model—was speculated [25, 26], based on the observation that all reported movement of particles with similar size of ribosomes involves Brownian motion [25, 27]. The Brownian ratchet scanning model proposes that the PIC can migrate along an mRNA in both 5′–3′ and 3′–5′ directions governed by Brownian motion and that the oscillation is directionally rectified through a ratchet-and-pawl mechanism: a “pawl” (i.e., possibly RNA-binding proteins) is occasionally placed on the mRNA at the trailing side of the PIC, restricting the 3′–5′ movement of the PIC beyond the pawl (Fig. 1A). In this model, even if an aAUG is missed by the PIC, it may be recognized in a second or subsequent scan as the PIC oscillates back and forth; a proximal dAUG, if present, can retain PICs that miss the aAUG, reducing the chance for a second (or more) inspection of the aAUG. In other words, while the strictly unidirectional scanning model relies on strictly sequential (5′–3′) decision-making involving more than one AUG in translation initiation, in the Brownian ratchet model initiation decisions are competitive between closely spaced AUGs.

The fundamental difference between the two models is whether the PIC can frequently move in the 3′–5′ direction, which can be experimentally determined by insertion of out-of-frame dATGs. The strictly unidirectional scanning model predicts that dATGs will have no effect on translation initiation of the canonical ORF, whereas the Brownian ratchet scanning model predicts that a dAUG can exhibit an inhibitory effect on initiation at the aAUG due to competition as the translation initiation site (Fig. 1A). Consistent with the strictly unidirectional model, previous work has shown that the first-AUG codon can exclusively serve as the site of translation initiation even when a second AUG is located within just a few nucleotides downstream [28], which is known as the first-AUG rule. However, other studies with genetically modified overlapping bicistronic mRNAs from *Turnip yellow mosaic virus* and *Influenza virus B* [29, 30] have revealed that the initiation frequency of an upstream ORF can be reduced by the presence of a proximal, downstream, and overlapping ORF, thus implying the presence of 3′–5′ PIC scanning at least in some sequence context [6, 12, 29]. These findings indeed raised many questions, and led us to investigate whether such 3′–5′ scanning observed in bicistronic viral mRNAs could also occur endogenously in monocistronic eukaryotic mRNAs.

Here, to compare the strictly unidirectional vs. Brownian ratchet scanning models, we generated thousands of green fluorescent protein (*GFP*) reporter gene sequence variants, each containing an out-of-frame ATG downstream of the ATG corresponding to the canonical ORF. We measured the fluorescence intensity of each variant, then performed computational simulations and estimated the leakage rate of each scan, as well as the number of scans for each triplet, before the ribosome eventually migrated to downstream regions of the mRNA. Our results reveal several general rules governing ribosomal scanning and enhance our understanding of how point mutations that introduce dATGs can lead to dysregulation of gene expression in human cells.

## Results

### Generation of thousands of dATG yeast variants

Previous systems studies have observed reduced protein abundance upon the addition of an out-of-frame uATG [17–19], indicating that PICs scan continuously along the mRNA in the 5′–3′ direction [4, 5, 15, 16]. By the same logic, a reduction in GFP intensity following the addition of an out-of-frame dATG will indicate that ribosomes can also scan in the 3′–5′ direction (Fig. 1A). In this study, we investigated the occurrence and prevalence of 3′–5′ PIC movement by inserting ATGs downstream of the aATG of a GFP reporter and then detecting the impacts on GFP synthesis (i.e., through differences in GFP intensity). To avoid reaching conclusions that are caused by some specific flanking sequences (i.e., confounding factors), as in viruses that may use specific sequences to regulate PIC scanning for overlapping ORFs in their bicistronic mRNAs [29, 30], we generated a large number of sequence variants, each with a dATG inserted in various sequence contexts (Fig. 1B).

Specifically, we introduced dATGs by chemically synthesizing a 39-nt DNA oligo, with six upstream and thirty downstream doped (i.e., random) nucleotides (*N* = 25% A + 25% T + 25% G + 25% C) around a fixed ATG triplet (designated as the aATG, Fig. 1B, Additional file 1: Fig. S1A). ATG triplets (either in-frame or out-of-frame) could then form randomly within the 30-nt downstream region of each individual construct, ultimately resulting in a randomly sampled variant library (from a huge number of all possible variants) containing dATGs at each successive position downstream of the aATG in various sequence contexts. To increase the fraction of dATG-containing variants, we further synthesized 28 additional DNA oligos, each with a dATG fixed at one of the 28 possible downstream triplet positions (Fig. 1B, Additional file 1: Fig. S1A). We fused these DNA oligos with the full-length *GFP* sequence (with its initiation codon omitted, Additional file 1: Fig. S1A), and integrated the fusion constructs individually into the same locus in Chromosome II of the yeast genome. We also inserted *dTomato*, encoding a red fluorescent protein, into a nearby genomic region to normalize GFP intensity (Fig. 1B, Additional file 1: Fig. S1A).

We measured the GFP intensities en masse through fluorescence-activated cell sorting (FACS)-seq for individual variants, as described in a previous study [31]. Briefly, we sorted yeast cells into eight bins according to GFP intensity (here and elsewhere in this study, normalized by dTomato intensity). Based on the variant frequencies in high-throughput sequencing reads of the eight bins, and the median GFP intensity and the number of cells belonging to each bin, we calculated the GFP intensity for each variant as the weighted average GFP intensity across the eight bins (Additional file 1: Fig. S1A).

To verify the accuracy of GFP intensities measured en masse, we randomly isolated 20 clones from the yeast library, and individually measured their GFP intensities by flow cytometry. There was good consistency between the GFP intensities measured en masse and individually (Pearson’s correlation coefficient *r* = 0.99, *P* = 1 × 10^−19^, Additional file 1: Fig. S1B). We measured GFP intensity of the yeast variants in two biological replicates, and the values were highly correlated for 18,950 variants shared between both experiments (Pearson’s correlation coefficient *r* = 0.99, *P* < 2.2 × 10^−16^, Additional file 1: Fig. S1C). Consequently, we pooled dATG variants from both replicates in subsequent data analyses (Additional file 1: Fig. S1D, the average GFP intensity was used for variants shared by the two replicates), if not otherwise specified.

We performed two positive control analyses to examine the data quality. First, the GFP intensity of variants with in-frame stop codons formed in the 30-nt region downstream of aATG was lower than that of variants without in-frame stop codons (*P* < 2.2 × 10^−16^, Mann-Whitney *U* test; Additional file 1: Fig. S1E). Second, the variants containing in-frame uATGs showed elevated GFP intensity compared to variants without uATGs (*P* < 2.2 × 10^−16^, Mann-Whitney *U* test; Additional file 1: Fig. S1F), most likely because the second in-frame AUGs could function as an auxiliary initiation site for GFP translation [32]. In contrast, the variants containing out-of-frame uATGs showed reduced GFP intensity (*P* < 2.2 × 10^−16^, Mann-Whitney *U* test; Additional file 1: Fig. S1F), likely because they can prevent translation in the reading frame of *GFP*. These observations bolstered our confidence to compare GFP intensities among the dATG variants in our study. Note that we excluded the variants containing in-frame stop codons or uATGs from the subsequent analyses, to avoid their potential impacts on GFP intensity (remaining variants *n* = 21,598, Additional file 1: Fig. S1D).

Both seminal studies analyzing the consensus sequence across genes [15, 33–35] and the recent structural analysis of the late-stage 48S initiation complexes [36] led to the hypothesis that some flanking sequences could facilitate translation initiation (known as the Kozak sequence). To determine if the sequences flanking the aAUGs exerted any detectable influence on the GFP intensities measured in our yeast library, we grouped the 1805 variants that had only one ATG (i.e., the designed aATG) in the 39-nt region, according to the nucleotide type at each position and estimated the average GFP intensity for each of the four variant groups at each position (Additional file 1: Fig. S2A). Briefly, placing different nucleotides at the −3 position (relative to the A[+1] in the aATG codon) led to the highest variation in GFP intensity compared to variation related to different nucleotides at other positions (from −6 to +15, Additional file 1: Fig. S2A). At the −3 position, “A” conferred the highest GFP intensity, followed by G, C, and finally T. This observation is qualitatively consistent with the prevalence of A at the −3 position among 96 yeast genes investigated in a previous study [33] or among the 500 genes with the highest protein synthesis rate in the yeast genome (Additional file 1: Fig. S2B). For simplicity, we hereafter refer to the ATG context using the nucleotide at the −3 position; in the order from “strong” to “weak” are the A, G, C, and T contexts. The observed differences in the strength of the sequence context are likely related to the frequency of leaky scanning, according to previous studies [15].

### Frame- and distance-dependent translational inhibition by dAUGs

Prior to measuring the effects of dAUGs on GFP intensity, we considered the variation in the number, position, and context of ATGs among the variants in the yeast library, to establish a standardized and clear nomenclature for these variants. Some variants had only one ATG in the 39-nt region (i.e., the designed aATG) and were therefore denoted as “Solo” variants. Some variants had one additional ATG in the 30-nt downstream region (i.e., the dATG) and were thus designated as “Duo” variants. In addition, the names of variants include the position and context of the aATG and dATG (if present). For example, Duo(1N, 4A) represents variants with two ATGs: the aATG having any one of the four nucleotides (N) at the −3 position and a dATG at the +4 position with an A in its −3 position (Fig. 1C). We subsequently focused on the analysis of 1805 Solo and 13,437 Duo variants.

In our design, dATGs were introduced at a total of 28 positions, among which ten were in-frame and 18 out-of-frame, relative to the *GFP* reading frame (Fig. 1C, Additional file 1: Fig. S1A). To investigate whether out-of-frame dAUGs can inhibit translation initiation from the aAUG, we grouped the Duo variants according to the reading frames of their dATGs. The results showed that the Duo variants containing in-frame dATGs showed elevated GFP intensity compared to Solo variants (*P* < 2.2 × 10^−16^, Mann-Whitney *U* test; Fig. 1D), as variants containing in-frame uATGs (Additional file 1: Fig. S1F). In sharp contrast, Duo variants harboring an out-of-frame dATG showed reduced GFP intensity compared to Solo variants (*P* = 3.9 × 10^−5^, Mann-Whitney *U* test, Fig. 1D), strongly suggesting that out-of-frame dAUGs can inhibit translation initiation at the aAUG in a frame-dependent manner. The fraction of reduction in GFP intensity for Duo variants relative to Solo variants is termed as the “inhibitory effect” subsequently.

To test if these inhibitory effects of out-of-frame dAUGs were dependent on the distance between aATG and dATG, we grouped the Duo variants according to the position of their dATG and then estimated the average GFP intensity for each group. The inhibitory effect gradually declined with increasing aATG-dATG distance (Fig. 1E, Additional file 1: Fig. S2C), and no inhibitory effects were evident at aATG-dATG distances of ~17 nt or greater (Fig. 1E, Additional file 1: Fig. S2C). These observations indicated that translation initiation decisions involving two proximal, potential AUGs were not strictly sequential, but competitive. Note that the placement of dATGs at various positions did not significantly alter the synonymous codon usage or the formation of mRNA secondary structure in the 30-nt variable sequence downstream of the aAUG (Additional file 1: Fig. S3), two factors known to affect translation initiation or elongation, and therefore, protein synthesis [37, 38].

We then performed an additional, small-scale experiment that strictly controlled the flanking sequence to further characterize the distance-dependent inhibitory effect of dAUGs. Specifically, we introduced an out-of-frame dATG at +8, +14, +20, or +26 positions downstream of the aATG (Fig. 1F). To exclude any potential impacts of the peptide sequence on GFP intensity, we used only synonymous mutations to introduce these out-of-frame dATGs. The results showed that proximal out-of-frame dATGs indeed reduced GFP intensity, while increases in distance between the two ATGs resulted in a gradual increase in GFP intensity. Beyond 20 nt, the negative impacts on translation initiation were no longer detectable (Fig. 1F). Collectively, these results established that out-of-frame dATGs could inhibit GFP synthesis and that these inhibitory effects decreased with increasing distance from the aATG.

### Context-dependent translational inhibition by dAUGs

The frame- and distance-dependent inhibitory effects of dAUG suggested that ribosomes could sometimes scan in the 3′–5′ direction, which was compatible with the Brownian ratchet scanning process wherein PICs oscillate in both 5′–3′ and 3′–5′ directions, scanning each successive triplet multiple times. An aAUG that is not recognized by the PIC in the first scan may be recognized in a subsequent scan. When a dAUG is inserted near the aAUG, a PIC that misses the aAUG may be instead retained by that nearby dAUG if it is recognized, thereby reducing the likelihood that a PIC will oscillate 3′–5′ and recognize the aAUG. As the aAUG-dAUG distance increases, there is an increased probability that a given PIC will turn to the 3′–5′ direction before encountering a dAUG, explaining why the inhibitory effect of out-of-frame dAUGs diminishes as the dAUG becomes farther.

**Fig. 2:**
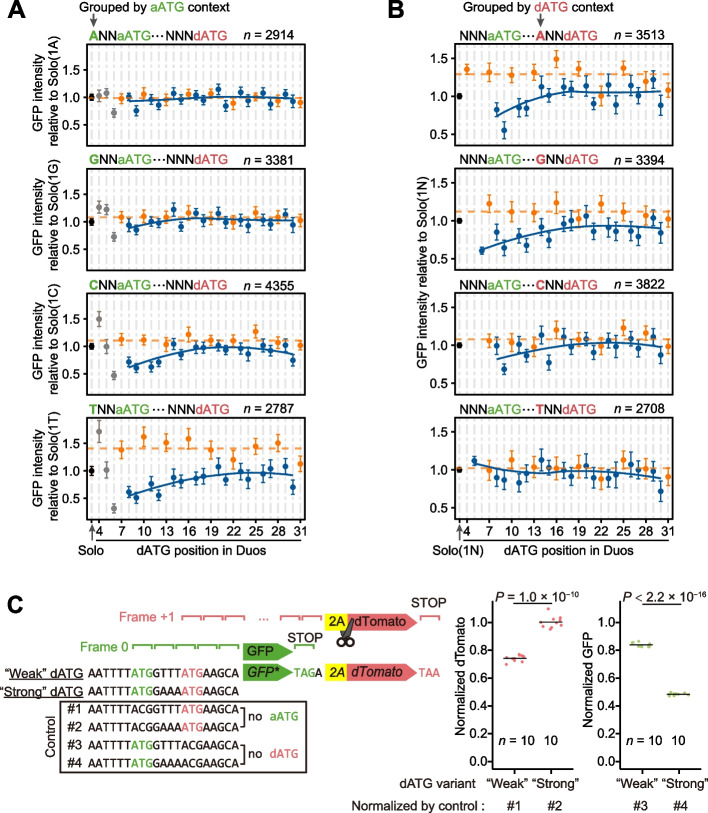
Context-dependent inhibitory effects on protein synthesis by proximal out-of-frame dATGs. **A, B** The average GFP intensities (dots) and the 95% confidence intervals (error bars) of Duo variants, grouped by the sequence contexts of the aATG or dATG (letters in green or red, respectively). The number of Duo variants drawn in each panel (*n*) is shown in its top-right corner (two biological replicates combined). The numbers of A, G, C, and T context Solo variants are 341, 383, 719, and 362, respectively. **C** The dual-frame reporter experiment to confirm the competition between aAUG and dAUG as the translation initiation site. The reporter is composed of a modified *GFP* gene in frame 0 (six stop codons were mutated in the +1 frame of its coding sequence, labeled as GFP*), sequences encoding a 2A self-cleaving peptide in frame +1, and a *dTomato* gene in frame +1. The fluorescence intensities of each dual-frame construct were normalized by the respective control construct as shown at the bottom. The normalized fluorescence intensities of individual replicates are shown by red or green dots and the mean is shown by the black line. *P* values were given by *t*-tests

The Brownian ratchet scanning model further predicted that the aAUG-dAUG competition depended on the leaky scanning at the aAUG. To test if the observed inhibitory effect of proximal out-of-frame dAUGs is indeed related to the leaky scanning at the aAUG, we divided the Duo variants into four groups based on their aATG −3 context. We found that the inhibitory effect of dATGs was greater when the aATG was in a weaker context (i.e., higher leakage rate, Fig. 2A, Additional file 1: Fig. S2D), which indicated that leaky scanning at aAUGs contributed to dAUG inhibition of translation initiation. To then determine whether these inhibitory effects were due to translation initiation at the dAUG, we also divided the Duo variants into four groups according to their dATG −3 context. We found that the inhibitory effect was greater when the dATG was in a stronger context, indicating the competition of translation initiation between the two AUGs (Fig. 2B, Additional file 1: Fig. S2D).

To confirm this apparent competition between aAUGs and dAUGs for translation initiation, we performed an experiment using a reporter construct carrying two fluorescent proteins, GFP and dTomato, encoded in different reading frames (hereafter referred to as a dual-frame reporter). In this reporter, GFP was translated from an aAUG in a weak context, and dTomato was translated from a proximal out-of-frame dAUG (+8 position, Fig. 2C). Furthermore, six “frame +1” stop codons were removed from the *GFP* coding sequence (mainly via synonymous mutations, see “Methods”) to avoid premature termination during dTomato translation. Placing the dATG in two different contexts, we measured both green and red fluorescence intensities with flow cytometry. We observed that dTomato intensity increased with increasing strength of dATG context (i.e., lower leakage rate) while GFP intensity was substantially reduced (Fig. 2C). Meanwhile, the mRNA levels did not significantly vary (Additional file 1: Fig. S4). These results confirmed that translation initiation decisions between two closely spaced AUGs were determined in a competitive manner.

### Proximal out-of-frame dAUGs lead to reduced mRNA levels via nonsense-mediated mRNA decay (NMD)

**Fig. 3:**
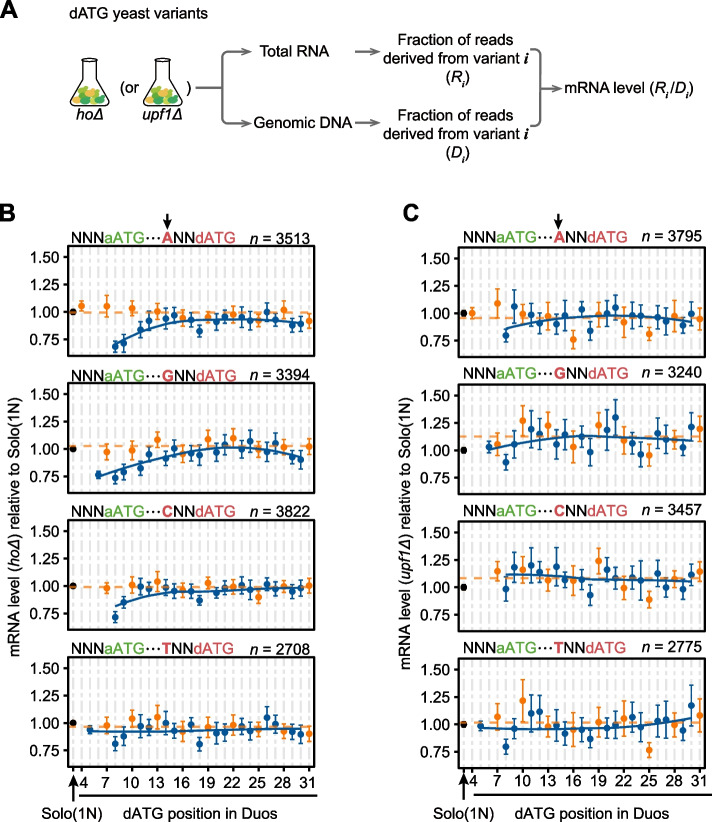
The reduction in the mRNA level caused by proximal out-of-frame dATGs, via the NMD pathway. **A** The experimental procedure for high-throughput determination of the mRNA levels for individual variants in the dATG library. **B, C** The average mRNA levels (dots) and the 95% confidence intervals (error bars) of Duo variants, in the background of *hoΔ* (**B**) and *upf1Δ* (**C**), grouped by the sequence contexts of the dATG (letters in red). Data from the two biological replicates were combined. The numbers of N-context Solo variants are 1805 (*hoΔ*) and 1989 (*upf1Δ*)

Our findings above thus suggested that proximal out-of-frame dAUGs could compete with aAUG for translation initiation. Since out-of-frame termination codons are abundant in the *GFP* coding sequence (see “Methods”), we predicted that if translation indeed initiated at a proximal out-of-frame dAUG, a long distance should remain between its (also out-of-frame) termination codon and the poly(A) tail, a signal for mRNA degradation by the NMD pathway [39–41]. To test if the insertion of proximal out-of-frame dAUGs can result in lower *GFP* mRNA stability, we measured the mRNA levels en masse for each variant in the library, as described in previous work [31]. Briefly, we used Illumina sequencing to determine the mRNA levels of each variant, which was normalized by the number of cells for each variant (as reflected by its fraction of sequencing reads in the DNA-seq, Fig. 3A). Since the mRNA levels of dATG variants were highly correlated between two biological replicates (Pearson’s correlation coefficient *r* = 0.86, *P* < 2.2 × 10^−16^, Additional file 1: Fig. S5A), we pooled dATG variants from both replicates in subsequent data analyses. We grouped the Duo variants according to the position of their dATGs, as well as by the aATG and dATG contexts. The results showed that mRNA levels were lower in the Duo variants when the out-of-frame dATG was closer to the aATG, particularly when the aATG resided in a weaker context (Additional file 1: Fig. S5B) and dATG resided in a stronger context (Fig. 3B), suggesting competition for translation initiation between closely spaced AUGs.

To then determine whether the reduction in the mRNA level was caused by NMD activity, we knocked out *UPF1*, the gene encoding an RNA helicase required for initiating NMD in eukaryotes [42, 43], and created a new yeast library containing a total of 15,256 variants in the background of *upf1Δ* (Fig. 3A). Note that in an effort to control for the potential cellular effects of the selective marker used for knocking-out *UPF1*, a yeast strain with a pseudogene (*HO*) deleted using the same selective marker was used as the wild type for yeast library construction throughout this study. We measured the mRNA levels of these variants and found that the reduction in mRNA levels we previously observed in Duo variants with proximal out-of-frame dAUGs was nearly abolished in the absence of *UPF1* (Fig. 3C, Additional file 1: Fig. S5C). These observations are consistent with the idea that the NMD pathway activated by translation initiation at out-of-frame dAUGs could reinforce the inhibitory effect of proximal dAUGs at the translational level.

To exclude the possibility that the distance-dependent inhibitory effect of out-of-frame dATGs is associated with variation in the activation efficiency for NMD, which has been reported depending on the position of the premature stop codon [41], we further computationally excluded variants that contained out-of-frame stop codons in the variable region in the same reading frame of the corresponding dATGs. After that, all Duo variants containing frame +1 (or +2) dAUG would terminate translation at the same location in the coding sequence of GFP (+56 or +60, see “Methods”). The NMD activity induced by proximal out-of-frame dATGs remained observed (Additional file 1: Fig. S5D), excluding the variation in NMD efficiency among dATG variants as a confounding factor.

To examine if the inhibitory effects of proximal out-of-frame dAUGs can be detected without the impact of NMD-related variation in mRNA stability, we used FACS-seq to measure GFP intensities in the genetic background of *upf1Δ* (Additional file 1: Fig. S6A). Despite full rescue at the mRNA level (Fig. 3C), NMD inactivation via *UPF1* deletion did not result in a full restoration of GFP intensity in these out-of-frame dATG variants (Additional file 1: Fig. S6B, C). These findings were further confirmed in small-scale experiments using the same dATG constructs as those shown in Fig. 1F (Additional file 1: Fig. S6D). Taken together, the inhibitory effects of proximal out-of-frame dAUGs persisted even controlling for the impact of NMD-related variation in mRNA stability, indicating direct competition between an aAUG and its proximal dAUG for translation initiation on the transcripts that have escaped NMD.

We surprisingly noticed that dATGs in frames +1 and +2 exhibited slightly different inhibitory effects, in both *hoΔ* and *upf1Δ* backgrounds (Additional file 1: Fig. S2D and Fig. S6C). This difference was not observed at the mRNA level (Fig. 3B, C, Additional file 1: Fig. S5B, C), suggesting that it was unlikely caused by the difference in translation initiation between dAUGs in these two frames. Instead, we hypothesized that this phenomenon was related to specific amino acids encoded in frame 0, provided that dATGs at frames +1 and +2 will lead to the overrepresentation of different amino acids in the N-terminus of the GFP reporter. To reduce the possible effects of sequence variation in the N-terminus peptide on GFP folding and fluorescence, we inserted a DNA sequence encoding a 2A self-cleaving peptide [44] upstream of the GFP coding sequence (Additional file 1: Fig. S7A). We controlled the sequence context of both aATG and dATG, generated 3402 Solo variants and 32,140 Duo variants, and performed the FACS-seq and the en masse RNA-seq experiments on this 2A-inserted dATG library (Additional file 1: Fig. S7B, C). The difference in GFP intensity between frame +1 and frame +2 dATGs was no longer detectable, and GFP intensity remained increasing with the aAUG-dAUG distance (Additional file 1: Fig. S7B). These observations further confirmed the inhibitory effect of proximal out-of-frame dAUGs.

### Distance-dependent translational inhibition by uAUGs

**Fig. 4:**
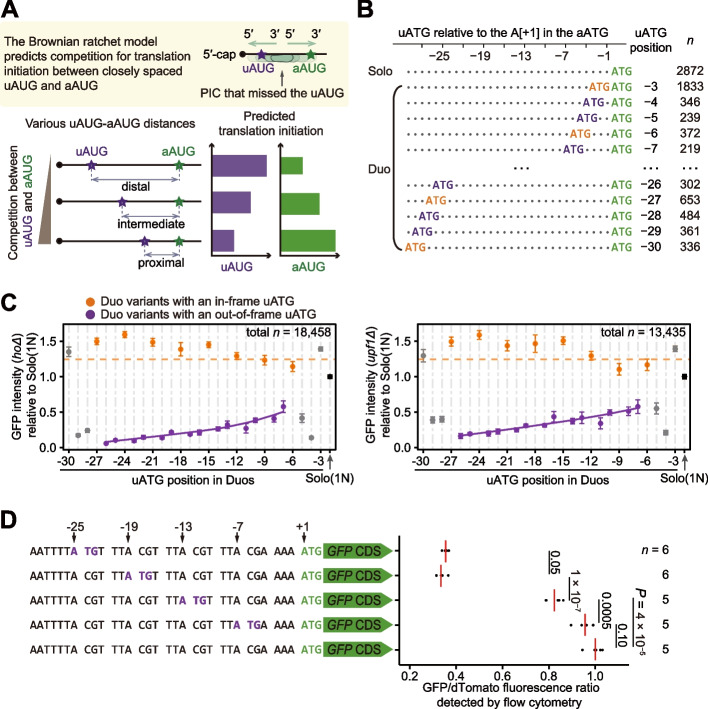
The distance-dependent inhibitory effect of out-of-frame uAUGs on translation initiation at the aAUG. **A** Predictions of the Brownian ratchet scanning model on translational initiation at the aAUG, when an out-of-frame uAUG is introduced at various positions. **B** Solo and Duo variants in the uATG library. The aATGs, in-frame uATGs, and out-of-frame uATGs are shown in green, orange, and purple, respectively. Each dot in the sequences represents a nucleotide that cannot form an ATG triplet or an in-frame stop codon for the uATG (if exists). **C** The average GFP intensities (dots) and the 95% confidence intervals (error bars) of Duo variants with the uATG placed at positions ranging from −30 to −3 in the *hoΔ* (left panel) and the *upf1Δ* (right panel) backgrounds. The orange dashed line represents the average GFP intensity of all Duo variants with an in-frame uATG, and the purple curve represents the local regression line (span = 1) for the Duo variants with an out-of-frame uATG. Duo variants with the uATG at positions from −5 to −3 had fixed nucleotides at the −3 position of the aATG (due to the uATG) and Duo variants with the uATG at positions from −30 to −28 had fixed nucleotides at the −3 position of the uATG (due to the upstream flanking sequence). These dots are shown in gray and were not used to fit the local regression line. **D** A small-scale experiment that strictly controlled the flanking sequence of uATG and aATG. The GFP/dTomato fluorescence ratios were normalized to the variant lacking additional uATG. *P* values were given by *t*-tests

In general, proximity to the 5′-cap grants an AUG triplet some advantages in competition to initiate translation since they are scanned first [6, 15]. Consistent with this hypothesis, it has been widely reported that out-of-frame uAUGs can inhibit translation at the aAUG because the uAUG can retain a proportion of PICs that would otherwise initiate translation at the aAUG [15, 19, 20]. Given our results showing competition for initiation between a closely spaced aAUG-dAUG pair, we further predicted that a closely spaced uAUG-aAUG pair would also compete for translation initiation. That is, when a uAUG is near the aAUG, a PIC that misses the uAUG (due to leaky scanning) may be retained by the nearby aAUG, thereby reducing the likelihood that the PIC will oscillate 3′–5′ and recognize the uAUG. Therefore, the Brownian ratchet scanning model further predicted that the inhibitory effect by an out-of-frame uAUG should diminish with decreasing uAUG-aAUG distance (Fig. 4A).

To test if the inhibitory effects of an out-of-frame uAUG indeed depend on its distance to the aAUG, we synthesized a uATG variant library (Fig. 4B) similar to the dATG variant library. Specifically, we introduced uATGs by chemically synthesizing a 30-nt DNA oligo with doped nucleotides (N) upstream of a fixed aATG triplet. To increase the proportion of variants carrying a uATG, we synthesized 28 additional DNA oligos, each with a uATG fixed at one of the 28 possible upstream triplet positions. We fused these DNA oligos with the full-length *GFP* sequence and integrated the fusion constructs individually into the yeast genome. GFP intensity and mRNA level of individual variants were then measured by FACS-seq and en masse RNA sequencing of the variable region, respectively, following the same protocol as used for the dATG library. GFP intensity and mRNA level were quantified in two biological replicates, and since the values were highly correlated between replicates (Additional file 1: Fig. S8), the data from both replicates were pooled in subsequent analyses.

The 3112 variants containing stop codons in the frame of uATGs and at a position upstream of the aATG were excluded from the subsequent analyses to avoid their potential impacts of translation reinitiation (i.e., the ability of some short upstream open reading frames to retain the 40S subunit on mRNA post-termination, then reinitiate translation at a downstream AUG). We confirmed that the 6553 Duo variants containing in-frame uATGs indeed showed higher GFP intensities than the 2872 Solo variants and the 9033 Duo variants containing out-of-frame uATGs indeed had lower GFP intensities (Fig. 4C, Additional file 1: Fig. S9). These results led us to further examine the impacts of uATG position relative to aATG, as well as uATG sequence context, on GFP intensities among the Duo variants.

To this end, Duo variants were grouped according to the position of their inserted uATGs, in a manner similar to that used for grouping dATGs in Fig. 1E. The results showed that GFP intensities increased with decreasing uATG-aATG distance, a trend which was especially apparent when the distance between the two ATGs was relatively small (Fig. 4C). We also observed that the inhibitory effect of a proximal, out-of-frame uATG was reduced in the variants harboring the aATG in a strong context (Additional file 1: Fig. S9A) or with a uATG in a weak context (Additional file 1: Fig. S9B). We then performed an additional, small-scale experiment in which the flanking sequence was strictly controlled in order to further characterize the distance-dependent inhibitory effects of uAUGs. Specifically, we introduced an out-of-frame uATG in a weak context (with a T in the −3 position) at positions −25, −19, −13, or −7 upstream of the aATG in a strong context (with an A in the −3 position, Fig. 4D). We observed that decreasing distance between the two ATGs indeed resulted in a gradual increase in GFP intensity (Fig. 4D). Taken together, these results showing distance- and context-dependent inhibitory effects by out-of-frame uATGs suggested that aAUGs compete with proximal uAUGs to initiate translation.

Translation initiation at out-of-frame uAUGs would result in the activation of the NMD pathway. Therefore, if the reduced inhibitory effect of proximal out-of-frame uAUGs did result from competition for translation initiation between the aAUG and a proximal uAUG, we predicted that *GFP* mRNA level should increase with decreased uAUG-aAUG distance. En masse quantification of mRNA levels for the out-of-frame uATG variants in the *hoΔ* background revealed that *GFP* mRNA level was higher in the variants with smaller uATG-dATG distance, weaker uATG context, and/or stronger aATG context (Additional file 1: Fig. S9C, D), and upon *UPF1* deletion, the *GFP* transcripts of Duo variants carrying an out-of-frame uATG were restored to levels comparable with that of Solo variants (Additional file 1: Fig. S10A, B), regardless of the uATG-aATG distance and the sequence context. Similar to the observation of the dATG variants, NMD inactivation also did not result in a full restoration of GFP intensity in the out-of-frame uATG variants of the uATG library (Additional file 1: Fig. S10C, D) or of the small-scale experiment (Additional file 1: Fig. S10E). These results thus indicated that the distance- and context-dependent inhibitory effect of out-of-frame uAUGs was indeed a consequence of competition for translation initiation between a pair of uAUG and aAUG.

### Computational modeling reveals that each successive triplet is on average scanned by the PIC approximately ten times

The competition for translation initiation we observed between closely spaced AUGs (either between an aAUG-dAUG pair or between a uAUG-aAUG pair) is qualitatively consistent with a scanning process in which the PIC is tethered to mRNA and progresses toward 3′-end under a Brownian ratchet mechanism and is inconsistent with a strictly unidirectional scanning process. It is worth noting that this observation would also be qualitatively compatible with other scanning models as long as PIC movement in both 5′–3′ and 3′–5′ directions is invoked. For example, some researchers have proposed that the PIC can move to the initiation codon via ATP-independent PIC “diffusion” along the mRNA [10, 45, 46]. Notably, the quantification of GFP intensity we conducted for thousands of variants in this study provided us with an opportunity to estimate the parameters of PIC scanning, such as the number of scans for each triplet, the frequency that a pawl (i.e., the 5′-block) is placed along the mRNA, and the efficiency of AUG recognition by the PIC. If the frequency of pawl placement is estimated to be zero, the diffusion model will be supported. On the contrary, the Brownian ratchet model will be supported if this frequency is greater than zero.

**Fig. 5:**
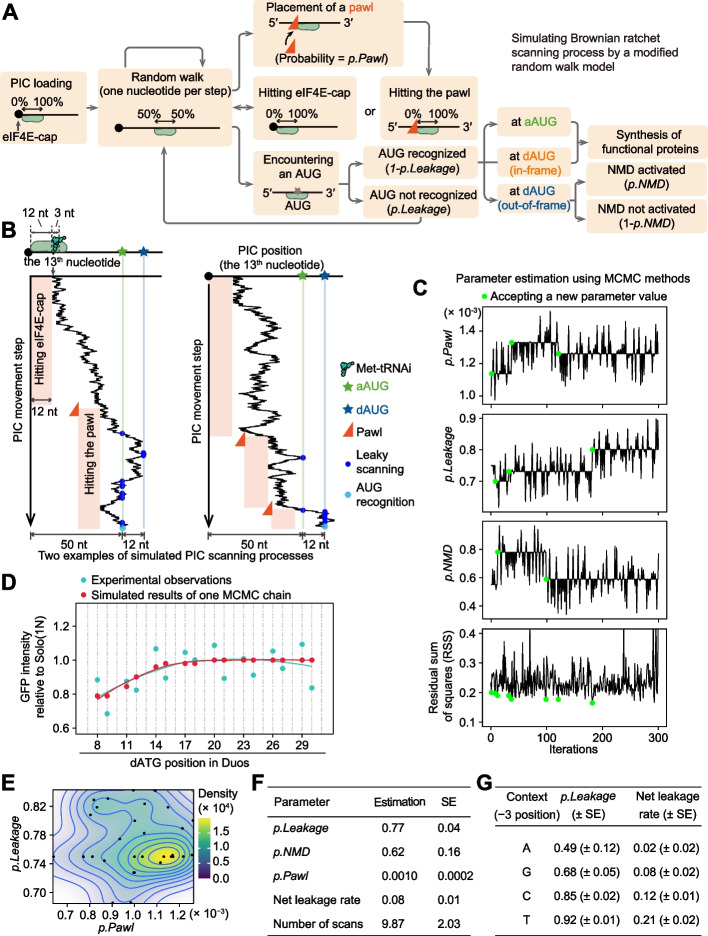
Estimation of parameters in the Brownian ratchet scanning model by MCMC algorithms. **A** A flowchart illustrating decisions involving PIC movement, translation initiation (or leakage), placement of a “pawl”, and activation of the NMD pathway. While it was reported that PICs loaded on the mRNA could move further upstream (5′) to scan AUGs having extremely short UTRs, this scenario has not been considered in our simulation because we have not yet known all the features regarding the steric hindrance between eIF4E-cap and the PIC mRNA-binding channel. **B** Two PIC trajectories exemplify the simulated PIC scanning process along the mRNA. According to Archer et al. [47], the 13th–15th nucleotides of a PIC-binding mRNA fragment are inspected for complementarity to the Met-tRNAi anticodon. Therefore, the 13th nucleotide of a PIC-binding mRNA fragment is used to plot the PIC position. **C** Trace plots show changes in values of *p.Pawl*, *p.Leakage*, and *p.NMD* in an MCMC chain. Green dots mark the MCMC iterations that accepted a new parameter value due to a reduction in the RSS. **D** The observed GFP intensities of out-of-frame dATG variants in the yeast experiments (two replicates combined) and the simulated GFP intensities using one of the 30 sets of optimized parameters by the MCMC algorithms. The results of all 30 sets of optimized parameters are shown in Additional file 1: Fig. S12. **E** Two-dimensional density plot shows the distribution of the outcome values of *p.Pawl* and *p.Leakage* among the 30 MCMC chains (shown by dots). Two additional density plots involving *p.NMD* are shown in Additional file 1: Fig. S11C. **F, G** Estimated parameters and standard error (SE) in the Brownian ratchet scanning model

To this end, we simulated the scanning process using a modified random walk model, as the PIC movement consists of a succession of random steps on the discrete positions along the “one-dimensional space” of a linear mRNA (Fig. 5A). We specified the following parameters in our random walk model. During scanning, the 13th–15th position of a PIC-binding mRNA fragment is the P-site [47], where the inspection for complementarity to the Met-tRNAi anticodon occurs. The PIC started out at the 5′-cap and took 1 nt per step in either the 5′–3′ or 3′–5′ direction, with equal probability (i.e., 50% each). However, the PIC could not move further upstream if its 5′-trailing side hits a pawl or the complex of 5′-cap and its binding protein eIF4E. The pawl was stochastically placed along the mRNA at the 5′-trailing side of a PIC (depending on the PIC location at the time) with the probability *p.Pawl* (Fig. 5A). When an AUG enters the P-site of the PIC, in a probability of *p.Leakage* the AUG might not be recognized by the PIC, and in a probability of (1 − *p.Leakage*) the AUG was recognized by the PIC and initiated translation. Note that in our model AUG triplets could be recognized in either 5′–3′ or 3′–5′ PIC movement. Considering that the NMD pathway can reduce mRNA levels and consequently amplify the impact of translation initiation at out-of-frame dAUGs on protein abundance, we used the parameter *p.NMD* to determine the probability of activating NMD when an out-of-frame dAUG is recognized by the PIC (Fig. 5A).

We employed a Markov Chain Monte Carlo (MCMC) algorithm [48, 49] to calculate numerical approximations for the probability parameters in the Brownian ratchet scanning model. To compare with experimental measurements of GFP intensity (Fig. 1E), we simulated the Brownian ratchet scanning process for 25 Duo variants with N-context dAUGs (representing an “average” dAUG) positioned between +7 and +31. To explore the relevant parameter space by the MCMC sampler, we first tested 1000 parameter sets for *p.Pawl*, *p.Leakage*, and *p.NMD* (each with ten values ranging from 0.001 to 0.8, Additional file 1: Fig. S11A). For each parameter set, we generated 100 simulations of the PIC scanning process for each Duo variant (two examples are shown in Fig. 5B) and estimated GFP intensity based on the number of simulations in which translation was initiated at the aAUG. We identified the ten parameter sets that showed the smallest residual sum of squares (RSS) for the 25 Duo variants and initiated the MCMC simulation using the median value for each parameter among the ten parameter sets: *p.Pawl* = 0.001, *p.Leakage* = 0.75, and *p.NMD* = 0.55 (Additional file 1: Fig. S11B).

We then ran the MCMC algorithm for 300 iterations, in which we sequentially replaced each of the three probability parameters with a random number generated from a uniform distribution (see “Methods”). For each iteration, we calculated the RSS from the simulated and observed GFP intensities in our yeast library, and used the RSS as a proxy to optimize the parameters. If the RSS decreased, the previous set of parameters was replaced by the new parameters, whereas if the RSS increased, the previous set of parameters remained unchanged (Fig. 5C).

The parameters reached the stationary distribution at the end of 300 iterations (Fig. 5C), and the GFP levels observed in the experiments were largely recapitulated by our simulated ratchet-and-pawl mechanism of PIC scanning (Fig. 5D). To obtain a reliable estimation of the parameters, we repeated 30 times of MCMC and found that the estimated parameters were robust after 300 iterations in all MCMCs (Fig. 5E, Additional file 1: Fig. S11C, Fig. S12). The average parameter values that resulted in the smallest RSS were as follows: the probability of adding a pawl to the mRNA was ~ 1 out of 1000 PIC steps; the average leakage rate for every single scan was 77%; and the NMD rate was 62% (Fig. 5F). Based on these values, we estimated that, on average, each triplet was scanned approximately ten times by the PIC (with 95% confidence intervals ranging from 6–14), resulting in a net leakage rate of 8% for a single AUG triplet (i.e., on average 8% of PICs eventually miss an AUG after multiple scans).

So far, we simulated using an N-context dAUG and neglected any difference in the leakage rate among sequence contexts of ATG triplets. To individually estimate *p.Leakage* for ATGs in the A, G, C, or T contexts, we fixed the values of *p.Pawl* (= 0.001) and *p.NMD* (= 0.62) and optimized context-specific *p.Leakage* by running the MCMC algorithm for another 100 iterations, based on the RSS estimated from the GFP intensities of variants Solo(1A), Solo(1G), Solo(1C), and Solo(1T) observed in our experiments (Fig. 2A). The average *p.Leakage* values among 30 times of MCMCs were 0.49, 0.68, 0.85, and 0.92 (Fig. 5G, Additional file 1: Fig. S11D), corresponding to the net leakage rates of 0.02, 0.08, 0.12, and 0.21, for ATGs in the A, G, C, or T context, respectively (Fig. 5G).

The Brownian ratchet scanning model and the ATP-independent diffusion model can be distinguished by determining if the probability of adding a pawl (*p.Pawl*) is equal to zero. *p.Pawl* was estimated to be significantly greater than zero (0.10% with the standard error equal to 0.02%, Fig. 5F) in our MCMC analyses, supporting a Brownian ratchet model for PIC scanning rather than a diffusion model. Note that a linear relationship between the length of the 5′-untranslated region (5′-UTR) and the time required for the first round of translation products was reported in previous studies [26, 50]; this relationship is also consistent with the Brownian ratchet scanning model instead of a diffusion model, which predicts a square relationship.

### Depletion of proximal out-of-frame dATGs in yeast and human genomes

**Fig. 6:**
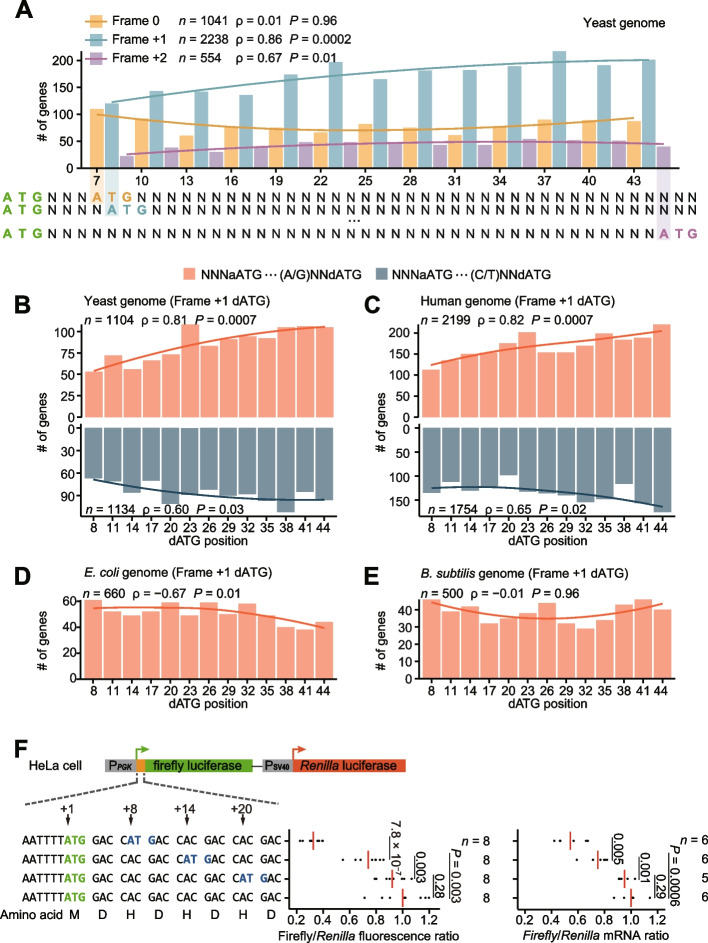
The depletion of proximal out-of-frame dATGs in eukaryotic genomes. **A** Meta-gene analysis shows the number of genes that harbor proximal dATGs at individual positions in the yeast genome. The curves represent the local regression line (span = 2). Spearman’s correlation coefficients (*ρ*) and corresponding *P* values are shown. **B–E** Numbers of genes that harbor frame +1 dATGs at individual positions in the genomes of yeast (**B**), humans (**C**), *Escherichia coli* (**D**), and *Bacillus subtilis* (**E**). *E. coli* and *B. subtilis* are used as negative controls as no ribosomal scanning mechanism is required for translation initiation in prokaryotes. **F** The dual-luciferase reporter experiment to test the distance-dependent inhibitory effect of dAUGs in HeLa cells. The values of individual replicates are shown by black dots and the average values are shown by red lines. The firefly/*Renilla* activity ratios were normalized to the variant lacking additional dATG, which were 0.33, 0.74, and 0.92 for the variants with dATGs inserted at the positions +8, +14, and +20, respectively. mRNA levels were measured with quantitative PCR and normalized to the variant lacking additional dATG. *P* values were given by *t*-tests

Given the reduced efficiency for translation of canonical ORFs and the possibility of enhanced synthesis of potentially cytotoxic peptides, we predicted that proximal out-of-frame dATGs would be generally deleterious. Therefore, we sought to test if proximal out-of-frame dATGs have been purged from the yeast genome by purifying selection. To this end, we counted the number of genes with ATGs at various positions downstream of the aATG across the yeast genome (Fig. 6A). The results showed that the number of out-of-frame dATGs increased gradually with distance from the aATG. The trend was statistically more significant in frame +1, probably because ~80% of out-of-frame dATGs are located in frame +1 due to the preferred usage of some amino acids or codons in frame 0. Moreover, the paucity of frame +1 proximal dATGs was particularly apparent for dATGs in the stronger context, suggesting that this paucity is related to translation at dAUGs (Fig. 6B; for results of frames 0 and +2, see Additional file 1: Fig. S13).

The detrimental impacts of proximal out-of-frame dATGs (e.g., synthesis of toxic peptides) should scale with the gene expression level. Therefore, the mutations that generate proximal out-of-frame dATG should be subject to stronger purifying selection in more highly expressed genes, leading to less proximal out-of-frame dATGs in these genes. Our sequence analyses showed that the 2000 genes with highest expression levels (or transcription rates) in the yeast genome indeed harbored less proximal out-of-frame dATGs than the 2000 genes with lowest expression levels (or transcription rates, Additional file 1: Fig. S14A, B). Collectively, the paucity of proximal out-of-frame dATGs, especially those in the stronger contexts and in more highly expressed genes, suggested that the purifying selection against proximal out-of-frame dATGs can exert a role in yeast genome evolution.

To test if proximal out-of-frame dATGs were also purged from other eukaryotic genomes, we similarly counted the number of frame +1 dATGs at various positions in the human genome. Similar to our analysis of the yeast genome, we found that the number of frame +1 dATGs increased with distance from the aATG, consistent with the observation in a previous study [51]. And the trend was more obvious for dATGs in a stronger context (Fig. 6C) and in more broadly expressed genes (Additional file 1: Fig. S14C). As a negative control, prokaryotes, which do not use the scanning mechanism to search the initiation codon [52, 53], did not show the depletion of proximal out-of-frame dATGs (Fig. 6D, E). These observations implied that the Brownian ratchet scanning process probably generally drove the evolution of eukaryotic genomes.

Although the translation machinery of yeast and humans is largely identical, some differences have been reported in the components of eIFs [6]. To test whether proximal out-of-frame dAUGs can indeed inhibit translation initiation at the aAUG in humans, we constructed a firefly and *Renilla* dual-luciferase reporter system and designed three additional variants, each with a frame +1 ATG introduced at a different location (+8, +14, and +20) downstream of the firefly luciferase aATG, using synonymous mutations (Fig. 6F). We transfected the reporters individually into HeLa cells and measured the *Renilla*-normalized firefly luciferase activity and mRNA level. In agreement with our findings in yeast, the results showed that proximal out-of-frame dATGs reduced firefly luciferase activity, partly contributed by the reduced mRNA level (which was likely caused by the NMD pathway activated by translation initiation at the out-of-frame dATG). Moreover, increases in the distance between the two ATGs resulted in a gradual increase in firefly luciferase activity, which was no longer distinguishable from the wild type in ATG variants located 20-nt downstream of the aATG (Fig. 6F). These results confirmed evolutionary conservation of the inhibitory effects on protein synthesis by proximal out-of-frame dATGs between humans and yeast.

## Discussion

### Brownian ratchet scanning process provides an explanation for the observed competition for translation initiation between closely spaced AUGs

The massive GFP intensity data generated in this study show context- and distance-dependent inhibitory effect of out-of-frame dAUGs on the translation of the canonical ORF (Figs. 1, 2, and 3). Furthermore, although it is well-known that an out-of-frame uAUG can inhibit translation of the canonical ORF, our data show that such an inhibitory effect decreases as the uAUG approaches the aAUG (Fig. 4). These observations indicate that competition for translation initiation exists between closely spaced AUGs, which undermines the first-AUG rule. Our computational modeling based on the experimental data then shows that the ribosome scanning process is better described as small-amplitude 5′–3′ and 3′–5′ oscillations that result in a net 5′–3′ movement (i.e., Brownian ratchet mechanism) instead of the conventional understanding of strictly unidirectional ribosome scanning (Fig. 5). We further show how such a scanning mechanism can influence the evolution of yeast and human genomes (Fig. 6).

The ribosome movement in the 3′–5′ direction has been reported in virus sequences. For example, Matsuda and Dreher modified the sequence of overlapping bicistronic mRNAs from the *Turnip yellow mosaic virus* and generated variants that contained two AUGs with various distances [29]. They showed competition for translation initiation between closely spaced AUGs, which were summarized as the evidence suggesting “limited relaxation over distances of a few nucleotides in the reverse direction” during PIC scanning [6, 12]. Similarly, a recent study reported that the small ribosomal subunit recruited by an internal ribosome entry site from poliovirus could initiate translation of an ORF in the upstream, suggesting the small ribosomal subunit had scanned in the 3′–5′ direction [54]. However, it remains unclear if the PIC movement in the 3′–5′ direction occurs only on a few specific (e.g., virus-related) sequences.

The massive GFP intensity data obtained in our experiments enabled excluding the possibility that the 3′–5′ PIC movement previously reported using a couple of variants was caused by specific flanking sequences, as well as excluding the effect of confounding factors (e.g., codon usage or mRNA secondary structure) on the rate of translation initiation. Furthermore, the massive GFP intensity data also provided us an opportunity to model the dynamics of ribosome scanning computationally and, in particular, to investigate how the net 5′–3′ PIC progression can be achieved from the small-amplitude 5′–3′ and 3′–5′ ribosome oscillations.

Our computational modeling of the massive GFP intensity data revealed a 77% average leakage rate for each single scan of an AUG triplet by the PIC and that the net leakage rate of 8% was achieved by multiple scans of the same triplet. A net leakage rate of 8% may ostensibly seem high considering the volume and variety of functional proteins that require accurate translation to maintain cellular function. However, we propose that this leakage rate is reasonable for two reasons. First, the addition of a second in-frame initiation codon as an auxiliary initiation site for translation initiation significantly elevates GFP levels (orange box in Fig. 1D), indicating that a substantial fraction of PICs appears to miss the aAUG and are instead captured by an in-frame, downstream AUG [32]. Second, the canonical ORFs are sometimes efficiently translated in genes bearing out-of-frame uATGs, which indicates that a significant proportion of uAUGs are not recognized by PICs.

Our calculation of a 77% average leakage rate for each single scan of an AUG triplet is indeed unexpectedly high. However, this high rate is intuitively consistent with data showing that a dAUG placed at +6 position (which has a G at its −3 position) can reduce GFP intensity by ~31% in the *upf1Δ* background (i.e., ~31% PICs that could have initiated at the aAUG were retained by a dAUG 5-nt apart, Additional file 1: Fig. S6B). Furthermore, when the aATG is in a weak context (with a T at its −3 position), the addition of a dATG at the +6 position even causes a ~50% reduction in GFP intensity in *upf1Δ* cells (Additional file 1: Fig. S6C). Similarly, Gu et al. reported that 50% of PICs were relocated to dAUG placed at +8 position (which has an A at its −3 position), as detected by toe-printing assays [54]. All these observations suggested a substantial single-scan leakage rate.

Computational modeling of the massive GFP intensity data suggested that the PIC movement in the 3′–5′ direction is unlikely some occasional relaxation as described in previous reviews [6, 12], but it is widespread that each triplet on average is scanned back and forth by the PIC for a dozen of times or so. While this number of scans may ostensibly seem high, we propose that this number is consistent with our experimental observations. Specifically, we showed that uAUG placed at the −7 position only reduced GFP intensity by ~42% in the *upf1Δ* background (Fig. 4C, right panel, here both uAUG and aAUG are in the N-context), clearly violating the first-AUG rule when two AUGs are sufficiently closely spaced. Similarly, dAUG placed at +9 position also reduced GFP intensity by ~22% in *upf1Δ* cells (Additional file 1: Fig. S6B). These phenomena cannot be well explained by occasional 3′–5′ PIC scanning but is expected when each triplet is scanned back and forth many times so that the first AUG only has a trivial advantage for translation initiation by being firstly scanned by the PIC.

Using an MCMC algorithm, we quantitatively investigate the Brownian ratchet scanning process by estimating the parameters that best fit the observed GFP intensity data in our dATG variant library. Note that although not highlighted in the “Results”, we also performed additional analyses to confirm the accuracy of parameter estimation. For example, we simulated the Brownian ratchet scanning process using the context-dependent *p.Leakage* (estimated from the Solo variants, Fig. 5G) and confirmed that the GFP intensities could be successfully recapitulated for specific aATG/dATG context combinations (Additional file 1: Fig. S11E). We also predicted GFP intensities without NMD activity (by setting *p.NMD* to zero) and confirmed that the GFP intensities in the *upf1Δ* background were largely recapitulated (Additional file 1: Fig. S11F). Moreover, we repeated the MCMC algorithms using the GFP intensity data of the uATG library (Additional file 1: Fig. S11G) and estimated the number of scans for each triplet to be 19.8, with 95% confidence intervals from 9–30 times (Additional file 1: Fig. S11H). This range overlapped with the 95% confidence intervals estimated from the dATG library (6–14 times). Cross-validation using various datasets confirmed the robustness of our computational modeling of the PIC scanning process.

Previous studies showed that the PIC scans at a rate of ∼6–8 nt/s in eukaryotic cell lysates based on the relationship between the 5′-UTR length and the time required for the first round of translation products [50] (a similar estimation of ~10 nt/s was obtained independently by another group [26]; see also [55] for a different estimation). However, it is puzzling that this estimated scanning rate is only approximately two times faster than the rate of translation elongation (~3–4.5 nt/s) measured in the same experimental conditions [50]. Note that PIC scanning only inspects for complementarity to the Met-tRNAi anticodon while translation elongation includes several complicated steps such as tRNA selection (which by itself includes multiple rounds of inspection of codon-anticodon complementarity), peptide bond formation, and translocation [56, 57]. We thus propose that PIC scanning rates measured in previous studies represent the “net” scanning rate, and can be better understood in the framework that each triplet is scanned back and forth: the actual scanning rate over individual nucleotides is approximately ten times faster than the net scanning rate, or ~60–100 nt/s.

### The relationship between the inhibitory effect of proximal out-of-frame dAUG and NMD activity

One of the major observations of this study is the inhibitory effects of a proximal out-of-frame dAUG on protein production (Figs. 1 and 2), which was used as evidence to support 3′–5′ PIC movement (i.e., the basis for the Brownian ratchet model). This apparent inhibitory effect has two potential sources: (i) the reduction in translation initiation rate at the aAUG because translation initiation at the dAUG consumes PICs and (ii) the elevated NMD activity induced by translation initiation at the out-of-frame dAUG. Both effects are the direct consequences of the competition for translation initiation between two closely spaced AUGs, and both contribute to the (total) inhibitory effects of proximal out-of-frame dAUGs on protein production.

We proposed that elevated NMD activity could be used to assess translation initiation from out-of-frame dAUGs. Note that gene transcripts may be subject to NMD activity for various reasons (e.g., transcription errors, mRNA damage, and/or translation initiation at a distal out-of-frame dATG in the GFP reporter sequence), which together account for the basal NMD activity of *GFP* mRNA in the Solo variants. In this study, the basal NMD activity has been controlled because we compared the expression levels of Duo variants to those of Solo variants. Our data showed that the NMD activity increased when proximal out-of-frame dAUGs were inserted (Fig. 3), a phenomenon that cannot be fully explained by a strictly unidirectional model only considering leaky scanning, which predicts that the NMD activity should be irrelevant to the aAUG-dAUG distance. Furthermore, the distance-dependent inhibitory effects of both out-of-frame dAUGs and uAUGs can be detected without the impact of NMD-related variation in mRNA stability (Additional file 1: Fig. S6B, C and S10C, D), illustrating that the effects on protein production induced by proximal out-of-frame AUGs were significantly contributed by mechanisms at the protein translation level.

### The observed inhibitory effect of proximal out-of-frame dAUGs cannot be explained by the steric hindrance effects

It is also possible in principle that the observed inhibitory effects of dAUG could be explained under the strictly 5′–3′ unidirectional scanning model, at least in part, by steric hindrance effects. That is, a PIC occupying a nearby dAUG waiting for translation initiation could reduce the aAUG accessibility to a 5′-trailing PIC [58], thereby reducing the translation initiation rate at the aAUG. Note that this explanation assumes sequential 5′–3′ decision-making by the PIC, and therefore predicts that the translation initiation rate at the dAUG is independent of the aAUG-dAUG distance. Since the NMD activity reflects the translation initiation rate at out-of-frame dAUGs, this explanation further predicts that the mRNA level of Duo variants should not be affected by the aAUG-dAUG distance. Our data showed that the *GFP* mRNA level also gradually declined with decreasing aAUG-dAUG distance (Fig. 3), an observation that is not well explained by the strictly unidirectional scanning model (even considering possible steric hindrance effects) but is compatible with the Brownian ratchet scanning model.

Furthermore, the Brownian ratchet model predicts that a closely spaced uAUG-aAUG pair would also compete for translation initiation, and therefore, the inhibitory effect by an out-of-frame uAUG should diminish with decreasing uAUG-aAUG distance (Fig. 4A). On the contrary, the strictly unidirectional model (and considering possible steric hindrance effects) predicts that the inhibitory effect of uAUGs is independent of the uAUG-aAUG distance because of sequential 5′–3′ decision-making by the PIC. Our data showed that GFP intensity increased in response to decreasing uAUG-aAUG distance (Fig. 4C, Additional file 1: Fig. S9A, B), an observation that is not well explained by the model involving the strictly unidirectional PIC movement (even considering possible steric hindrance effects) but fits well with the Brownian ratchet scanning model. Also note that the distance-dependent inhibitory effect of uAUGs persisted in *upf1Δ* cells (Fig. 4C, Additional file 1: Fig. S10C, D), indicating that the phenomenon cannot be fully explained by the variation in NMD activity associated with the efficiency of translation initiation at the out-of-frame uAUG.

### Previous findings explainable by the Brownian ratchet scanning process

Some seminal experiments showed that immediately post-termination, ribosomes can scan in the 3′–5′ direction and thereby reinitiate translation at a nearby AUG triplet upstream of the stop codon [59–62]. This finding suggests that the 40S ribosomal subunit has an intrinsic capability to migrate in both the 5′–3′ and 3′–5′ directions along unstructured mRNAs [10, 59, 63]. However, these observations differ from our findings reported here, since it remains unknown if the 3′–5′ movement described in previous studies only occurs before the post-termination ribosomes have recruited sufficient eIFs required for the “normal” scanning process that starts at the 5′-cap [59, 64].

Furthermore, it was reported that in case guanosine triphosphate hydrolysis does not occur on time, the small ribosomal subunit that had successfully recognized an AUG codon was capable of resuming “sliding” to search for AUGs [65]. While a proximal dAUG could be efficiently recognized in this sliding process, farther dAUGs were more hardly recognized, as detected by the toe-printing assay [65], which is explainable if the ribosome uses small-amplitude 5′–3′ and 3′–5′ oscillations to search for AUGs. These observations again support that scanning or sliding in both the 5′–3′ and 3′–5′ directions is an intrinsic capability of the small ribosomal subunit.

Previous studies reported that 80S ribosome pauses could trigger the stacking of ribosomes and promote translation initiation [66, 67]. This phenomenon is also explainable under the Brownian ratchet model because paused ribosomes block the 5′–3′ proceeding of PICs, which can elevate the probability of 3′–5′ movement of the PIC, thereby increasing the number of scans for an AUG by the PIC. In contrast, this phenomenon is not easily explained by the strictly unidirectional scanning model that each triplet is scanned only once. Note that the distance between the pausing site and the AUG triplet reported in these studies (43 and 144 nt, respectively) was much greater than the distance between AUGs competing for initiation as reported in our study (< 17 nt), therefore reflecting a different facet of the Brownian ratchet scanning process (i.e., when the PIC movement in the 5′–3′ direction is restricted). Similarly, the Brownian ratchet model can also help explain the phenomenon in a human cell line that translation initiation at uAUGs substantially increased upon the treatment of 3 μM Rocaglamide A. Rocaglamide A specifically increases eIF4A’s binding affinity with polypurine RNA sequences, which likely blocks 5′–3′ PIC movement and elevates the probability of 3′–5′ movement, thereby increasing the probability that a uAUG is recognized by the PIC. Consistently, the observed translation initiation sites were often ~24 nt upstream of the Rocaglamide A binding sites [68].

It remains controversial as to how eIF4E-bound mRNA initially enters the mRNA-binding channel of the small ribosomal subunit. A “slotting” model proposes that eIF4E-bound mRNAs “slot” directly into the mRNA-binding channel whereas a “threading” model hypothesizes that mRNAs “thread” into the mRNA-binding channel after disruption of the eIF4E-cap interaction. The “slotting” model was supported by the exit-tunnel location of eIF4E relative to the translational initiation complex in a structural analysis [69] and by the observation of 5′-UTR length-dependent translation inhibition by tethering eIF4E to the 5′-cap [54]. The “slotting” model would further predict the existence of a translation “blind spot” (i.e., AUG triplets sufficiently close to the 5′-cap cannot be recognized due to the steric hindrance between eIF4E-cap and the small ribosomal subunit) under a strictly unidirectional scanning model [70], and this prediction is contradictory to the observations that eukaryotic mRNAs with extremely short 5′-UTRs could still initiate translation [54, 70, 71]. Nevertheless, this apparent contradiction can be resolved by considering the Brownian ratchet scanning process because AUGs close to the 5′-cap can still be scanned through a 3′–5′ PIC movement after the eIF4E-bound mRNA is “slotted” into the mRNA-binding channel.

### Future directions

Our findings in this study suggest at least three major directions for future experimental exploration. First, it would be of great value to confirm or refute the Brownian ratchet model by tracing the movement of a single PIC along an mRNA with super-resolution light microscopy or optical tweezers, in real-time and at single-nucleotide resolution, in an effort to observe small-amplitude 5′–3′ and 3′–5′ oscillations with a net 5′–3′ movement. Second, the quantification of the consumption of ATP usage for PIC scanning along unstructured mRNAs would also help estimate the parameters involved in the Brownian ratchet scanning model, such as the frequency of pawl placement onto mRNAs [25, 72]. Third, the identification of eukaryotic initiation factors that participate in the Brownian ratchet scanning mechanism (e.g., the protein identity of the “pawl”) could also offer insight into the mechanism by which directional PIC movement can be achieved from PIC diffusion in both 5′–3′ and 3′–5′ directions [25]. A recent study estimated a net scanning rate of ∼100 nt/s in a reconstituted translation system [55]; this rate is an order of magnitude faster than the net scanning rate reported based on cell lysates in two previous studies [26, 50] and is similar to the scanning rate over individual nucleotides estimated in our study. We suspect that PICs migrate at a faster rate in the reconstituted system due to the lack or excess of some translation-related factors so that PICs do not change directions as often as in cells. Therefore, additional investigation into the compositions of such reconstituted translation systems could shed light on the mechanisms regulating the Brownian ratchet scanning process. It is also possible that 5′-trailing PICs may serve as a pawl that prevents backward scanning of 3′-leading PICs since several previous studies reported that multiple PICs could scan simultaneously on the same 5′-UTR [47, 73, 74] (i.e., the 5′-cap becomes unattached to the scanning PIC after recruitment of the small ribosomal subunit, also known as the “cap-severed” model).

In addition, our findings also have potential medical implications. Specifically, the currently common presumption by experimental biologists and medical scientists that disease-associated, translational defect mutations are likely associated with uAUGs [22, 75] should be expanded to include inhibitory effects of proximal, out-of-frame dAUGs on the translation of canonical ORFs. This new insight will help in computational predictions of disease-causing mutations from whole genome/exome sequencing data in the future.

## Conclusions

Proximal out-of-frame dAUGs can reduce protein production of the canonical ORFs in a context-dependent manner. The inhibitory effect of out-of-frame uAUGs diminishes with the decrease of the uAUG-aAUG distance. These phenomena violate the first-AUG rule and indicate the competition for translation initiation between closely spaced AUGs. The massive GFP intensities measured in this study are quantitatively consistent with the Brownian ratchet model of PIC scanning rather than a strictly unidirectional scanning model. Proximal out-of-frame dATGs are purged from eukaryotic genomes during evolution by purifying selection.

## Methods

### Construction of the yeast variant library

We constructed the yeast dATG library as described in a previous study [31]. Specifically, we first constructed a yeast strain (BY4742-dTomato, *MAT*α *his3Δ1 leu2Δ0 lys2Δ0 ura3Δ0 gal7Δ0::dTomato-hphMX*) which expressed dTomato from the *GAL7* promoter in the background of BY4742 [76], using a recombination-mediated polymerase chain reaction (PCR)-directed allele replacement method (primers provided in Additional file 2: Table S1). The dTomato expression would be later used to normalize GFP intensity, which in principle could be affected by cell-to-cell variation in galactose induction and cell-cycle status [77]. We selected the transformants on the 1% yeast extract–2% peptone–2% dextrose (YPD) solid medium with 200 μg/ml hygromycin B (Amresco, Cat#97064–454). We performed PCR on the extracted genomic DNA of the yeast transformants to verify the successful *GAL7* deletion and *dTomato* integration (here and also hereafter when genetic manipulation was performed).

To construct the dATG yeast library in the background of *upf1Δ*, we further deleted *UPF1* in the background of BY4742-dTomato using recombination-mediated PCR-directed allele replacement method (BY4742-dTomato-*upf1Δ*, primers provided in Additional file 2: Table S1). To this end, we amplified the *natMX* cassette from plasmid PAG25 (Addgene, Cat#35121), transformed the PCR product into BY4742-dTomato, and selected the transformants on the YPD solid medium with 100 μg/ml nourseothricin (Amresco, Cat#6021-878). To control for the potential cellular effects of *natMX* expression when *GFP* expression in the background of *upf1Δ* was compared with that in the wild type, we replaced the gene encoding homothallic switching endonuclease, *HO* (a pseudogene in the BY4742 background), using *natMX* [78]. The resultant yeast strain (i.e., BY4742-dTomato-*hoΔ*) was used as the wild type in this study.

We chemically synthesized 29 oligos with specific positions using doped nucleotides (Fig. 1B, Additional file 1: Fig. S1A, Additional file 2: Table S2); 28 of them contained an ATG designed at a particular position downstream of the aATG. We mixed these oligos and fused them with the *GAL1* promoter, the full-length *GFP* coding sequence (CDS, with the initiation codon of *GFP* omitted, including a ten amino-acid “linker” sequence in its N-terminus), the *ADH1* terminator, *URA3MX*, and the *GAL1* terminator (in the order shown in Additional file 1: Fig. S1A), using fusion PCR and the GeneArt Seamless Cloning and Assembly Kit (Thermo Fisher, Cat#A14606). The resultant sequence surrounding the synthetic oligo is CTT TAA CGT CAA GGA GAA AAA (NNN NNN ATG NNN NNN NNN NNN NNN NNN NNN NNN NNN NNN) *GCA GGT CGA CGG ATC CCC GGG Tta aTt aaC* AGt aaA GGA GAA GAA CTT TTC ACT GGA GTT GTC CCA ATT CTT GTt gaA Tta gAT GGt gaT GTt aaT GGG CAC AAA TTT, where out-of-frame dATGs and out-of-frame stop codons are underlined and shown in lowercase letters, respectively. Thirty nucleotides encoding the “linker” sequence are italicized.

To construct the dATG yeast library, we transformed the PCR product to replace the coding sequence of *GAL1* in the BY4742-dTomato-*hoΔ* strain (Additional file 1: Fig. S1A). Specifically, the *GAL1* promoter (500 nt) and *GAL1* terminator (500 nt) were used as long homologous sequences to allow efficient integration of the PCR product into the yeast nuclear genome [31]. We selected successful transformants in the synthetic complete medium (dextrose as the carbon source) with uracil dropped out. We collected a total of ~50,000 yeast transformants, which most likely contained various sequences surrounding the aATG of *GFP*, due to the huge number of possible sequences that could be generated from the synthesized oligos (4^36^ ≈ 5 × 10^21^). We similarly constructed the dATG yeast library in the background of BY4742-dTomato-*upf1Δ*, and collected a total of ~60,000 yeast transformants*.*

We also constructed a 2A-inserted dATG yeast library to eliminate the potential impacts of nonsynonymous substitutions introduced by the doped nucleotide in the N-terminus of the GFP reporter. To this end, we inserted a DNA sequence encoding a 2A self-cleaving peptide [44] (GGT TCT GGT GGT GCT ACT AAT TTT TCT TTG TTG AAA TTG GCT GGT GAT GTT GAA TTG AAT CCA GGT CCA) between the “linker” sequence and the *GFP* CDS. The construction procedure for the 2A-inserted dATG variant library is otherwise similar to the protocol aforementioned for the dATG variant library, except that the oligos synthesized to introduce dATGs in this 2A-inserted dATG yeast library had a fixed trinucleotide sequence upstream of the designated aATG (TTT, “weak” context) and a fixed trinucleotide sequence upstream of each dATG (AAA, “strong” context, whenever not overlapped with the aATG, see Additional file 2: Table S2 for details).

The uATG yeast library was similarly constructed in both *hoΔ* and *upf1Δ* backgrounds (Fig. 4B), with the sequence structure CTT TAA CGT CAA GGA GAA AAA TTT (NNN NNN NNN NNN NNN NNN NNN NNN NNN NNN) atg GCA GGT CGA CGG ATC CCC GGG TTA ATT AAC AGT AAA GGA, where the designated aATG is shown in lowercase letters (see Additional file 2: Table S2 for details).

### FACS-coupled high-throughput sequencing (FACS-seq)

We gauged GFP intensities for each of the thousands of yeast variants using a high-throughput strategy, FACS-seq, as described in a previous study [31]. Specifically, we pooled yeast variants that contained various sequences surrounding the aATG of *GFP* and cultured them in the liquid medium (YPGEG) that contained 1% yeast extract, 2% peptone, 2% glycerol and 2% ethanol (both served as the carbon source), and 2% galactose (to induce the expression of *GFP* and *dTomato*). We harvested yeast cells after 18 h, in which duration the optical density at 660 nm increased from ~0.1 to ~0.7. We nitrogen froze half of the harvested cells for total RNA and DNA extraction (to perform RNA-seq and DNA-seq as described below), and re-suspended the other in the 1× phosphate-buffered saline for FACS-seq.

We sorted yeast cells into eight bins using Aria III cytometer (BD Biosciences) based on the intensity ratio of GFP and dTomato fluorescence, which were excited by 488- and 561-nm lasers and were detected using 530/30- and 610/20-nm filters, respectively. We recorded the median GFP/dTomato intensity ratio for each bin, as well as the proportion of cells belonging to the “gate” of each bin (Additional file 1: Fig. S1A). We collected at least 20,000 yeast cells for each bin and cultured them individually in YPD overnight at 30°C to amplify the cell population for easier DNA extraction. Since GFP was not expressed in YPD (therefore should confer limited fitness cost), the relative fraction of yeast variants in each bin should be largely maintained during this amplification.

We extracted the genomic DNA from yeast cells of each bin and performed two rounds of PCR amplification on the variable region (e.g., 6-nt upstream and 30-nt downstream of the *GFP* aATG for the dATG library) to construct the Illumina sequencing libraries. Taking the dATG library for example, in the first round PCR, a pair of sequences identical to the 21-nt sequence upstream of aATG (positions −35 to −15, relative to the A[+1] of the aATG) and the reverse complement of the 20-nt sequence downstream of the aATG (positions +45 to +64) were used to amplify the variable region (primer sequences for other yeast libraries are provided in Additional file 2: Table S1). Meanwhile, we introduced 19-nt or 21-nt sequences identical to the 3′-end of the P5 or P7 adaptor, respectively, a 12-nt stretch of random nucleotides (NNNNNNNNNNNN, designed to avoid difficulty in base calling of Illumina sequencing when sequencing “constant” region), and a 6-nt bin-specific barcode to the ends of the PCR product (Additional file 1: Fig. S1A, Additional file 2: Tables S1, S3). In the second round PCR, the full-length P5 and P7 adaptors as well as the sequencing indices were added to the ends. The PCR products were then subject to Illumina sequencing (NovaSeq 6000 platform, in the PE150 mode).

### Small-scale validation of fluorescence intensities using flow cytometer

We randomly isolated 20 yeast variants from the yeast dATG library from individual colonies on the solid medium, and sequenced the variable region for each variant by Sanger sequencing. We induced the expression of the fluorescent proteins in the liquid YPGEG medium for individual strains and harvested yeast cells in the mid-log phase. For each strain, we measured the GFP and dTomato fluorescence intensities by Aria III cytometer using the same settings as in the FACS-seq experiment.

### RNA-seq and DNA-seq for the yeast variant library

We extracted the total RNA from the harvested cells of the yeast library (cultured in the YPGEG liquid medium for 18 h) and performed reverse transcription using the GoScript^TM^ Reverse Transcription System (Promega, Cat#A5001). We built the Illumina sequencing library by two-round PCR amplification of the variable region (similarly to FACS-seq, primers provided in Additional file 2: Table S1). Illumina sequencing was performed on the NovaSeq 6000 platform under the PE150 mode. To control for the variation in the cell number among the yeast variants and the potential bias in Illumina sequencing, we also extracted the total genomic DNA from the harvested cells and PCR-amplified the variable region for Illumina sequencing (primer sequences provided in Additional file 2: Table S1).

Two biological replicates were performed for the dATG library, the 2A-inserted dATG library, or the uATG library by independently inducing GFP and dTomato expression. In each replicate, we performed RNA-seq and DNA-seq on the same group of harvested cells used for the FACS-seq experiments.

### Dual-fluorescence reporter assay in yeast

We determined the distance-dependent inhibitory effect of out-of-frame dAUGs on translation initiation at the aAUG in small-scale experiments, using a dual-fluorescence reporter assay. To this end, we first constructed *TEF* promoter-*GFP* CDS-*CYC1* terminator-*TDH3* promoter-*dTomato* CDS-*ADH1* terminator-*URA3MX* in the background of pUC57 plasmid (GenBank: Y14837.1) using the GeneArt Seamless Cloning and Assembly Kit (sequence shown in Additional file 2: Table S4). Then, with this plasmid as the template, we introduced an out-of-frame dATG at the position +8, +14, +20, or +26 by inserting a 27-nt sequence downstream of the aATG of *GFP* using fusion PCR (primer sequences provided in Additional file 2: Table S1). A control variant lacking additional dATG was also constructed.

We inserted each of the five variants into the BY4742 genome by replacing the endogenous *HO* locus in Chromosome IV, using recombination-mediated PCR-directed allele replacement method (59-nt homologous sequences in both ends, primer sequences are provided in Additional file 2: Table S1). We harvested yeast cells in the mid-log phase and used Accuri^TM^ C6 cytometer (BD Biosciences) to measure the GFP and dTomato fluorescence (excited by 473- and 552-nm lasers and detected with 530/30- and 610/20 nm filters, respectively). The reported GFP fluorescence intensity was normalized by the dTomato fluorescence intensity. We similarly performed the experiments for these five variants in the *upf1Δ* background.

We constructed four uATG variants, each with a uATG inserted at the position −25, −19, −13, −7, and one control variant without uATG in the backgrounds of *hoΔ* and *upf1Δ*, and performed dual-fluorescence reporter assay. Primer sequences used for fusion PCR are provided in Additional file 2: Table S1.

### Dual-frame reporter assay in yeast

To detect the translational competition between two closely spaced AUGs, we designed a dual-frame reporter, in which GFP (frame 0) and dTomato (frame +1) were encoded in the same transcript expressed from the *TDH3* promoter (Fig. 2C). To avoid truncated protein in frame +1, we removed all six “frame +1” stop codons in the *GFP* CDS (five of them via synonymous mutations). A “frame +1” stop codon residing in ATG AAA (coding Met-Lys in GFP) could not be removed via synonymous mutations, so we replaced Met with its most exchangeable amino acid (according to the BLOSUM matrix), Leu, resulting in a sequence of “CTT AAA.” To minimize the influence of the long peptide in the N-terminus (peptide sequence encoded by frame +1 of the *GFP* CDS) on protein folding of dTomato, we inserted a 2A self-cleaving peptide in frame +1 right upstream of the *dTomato* CDS. The dual-frame reporter DNA was synthesized by BGI Tech (sequence shown in Additional file 2: Table S4), based on which we generated two dual-frame constructs with 3-nt difference in the sequence upstream of the dTomato ATG and four control constructs lacking either the GFP ATG or the dTomato ATG (Fig. 2C).

We inserted the dual-frame reporter constructs into the yeast genome, which replaced the endogenous *HO* locus in BY4742, by recombination-mediated PCR-directed allele replacement method (59-nt homologous sequences in both ends, primer sequences provided in Additional file 2: Table S1). We harvested yeast cells in the mid-log phase for each yeast strain and measured the GFP and dTomato fluorescence using Accuri^TM^ C6 cytometer and mRNA levels using the Bio-Rad CFX384 Touch real-time PCR detection system (PCR primers are provided in Additional file 2: Table S1).

### Dual-luciferase assay in HeLa cells

HeLa cells were cultured in Dulbecco’s modified Eagle’s medium containing 10% Fetal Calf Serum, 2 mM L-glutamine at 37°C in a 5% CO_2_ incubator. To detect the inhibitory effect of proximal out-of-frame dAUGs on translation initiation at the aAUG in HeLa cells, we performed a dual-luciferase assay based on modified pmirGLO plasmids (Promega, Cat#E1330), in which firefly and *Renilla* luciferases were individually expressed from *PGK* and SV40 promoters, respectively. Specifically, using site-directed mutagenesis methods, we modified the pmirGLO plasmids by introducing a 6-nt sequence (AATTTT, weak context) right upstream of the firefly luciferase ATG to increase its leakage rate. We designed synonymous mutations to generate four 21-nt sequences that encode the same amino acid sequence; one sequence lacked proximal out-of-frame dATGs and the other three each contained a proximal out-of-frame dATG at the +8, +14, or +20 position relative to the aATG (Fig. 6F).

We introduced each of the four 21-nt sequences right downstream of the firefly luciferase ATG in the plasmid using site-directed mutagenesis, and individually transfected the four modified plasmids into HeLa cells using Lipofectamine^TM^ 2000 (Thermo Fisher, Cat#11668030). We determined the activities of luciferases in 96-well microliter plates 48 h after transfection, using a commercial dual-luciferase assay kit (Promega, Cat#E1910) following the manufacturer’s protocol. Briefly, we lysed HeLa cells using 500 μL of passive lysis buffer and mixed 20 μL suspension with 100 μL firefly luciferase substrate. We first measured firefly luciferase activity using the Synergy HTX multi-mode microplate reader (BioTek). Then, we added 100 μL of Stop-and-Glo reagent to the solution and measured the *Renilla* luciferase activity using the same equipment. mRNA levels were determined using the Bio-Rad CFX384 Touch real-time PCR detection system (PCR primers are provided in Additional file 2: Table S1).

### Quantification of GFP and mRNA levels for individual variants in the yeast libraries

For the dATG variant library, the “read 1” of a read pair from the DNA-seq data should follow the pattern of N(12)-barcode (6 nt)-CCTCTATACTTTAACGTCAAGGAGAAAAA-N(6)-ATG-N(30)-GCAGGTCGACGGATCCCCGGGTTAATTAACA-barcode (6 nt)-N(12)-P7. Note that P7 adaptor would also be sequenced downstream of the inserted sequence because the length of the inserted sequence of the Illumina sequencing library generated in this study was 135 nt, shorter than that of a sequencing read (150 nt). For the same reason, the reverse complements of the barcodes and the variable region would be sequenced for a second time in the “read 2”, in which part of the P5 adaptor would be sequenced. For each read, we extracted the barcodes (6-nt upstream and 6-nt downstream) as well as the 36-nt variable sequence surrounding the ATG using pattern matching. We discarded the whole read pair in the following three scenarios: (1) if either read of a read pair could not match to the pattern, (2) if any of the four barcodes extracted from a read pair was different from the barcodes that were introduced during library preparation for a particular sample, or (3) if the read 1 sequence and the reverse complement sequence of the read 2 were not identical in the variable region. We then classified read pairs into biological replicates according to the barcode sequence and grouped read pairs into variants according to the sequence in the variable region. The sequencing data from the RNA-seq and FACS-seq libraries were similarly analyzed. The numbers of read pairs that passed the three criteria and the numbers of identified variants are summarized in Additional file 2: Table S5.

Some sequences in the variable regions were not detected in all three libraries (FACS-seq, RNA-seq, and DNA-seq), which implied that they were potentially originated from PCR amplification errors during Illumina sequencing library preparation. We therefore discarded the variants that did not appear in all three libraries. Furthermore, the frequencies of some dATG variants appeared to be too low in the DNA-seq (number of read pairs ≤8) and FACS-seq libraries (all read pairs from the eight bins combined ≤64). To be conservative, we also discarded these variants (the remained variant numbers are shown in the Venn diagram of Additional file 1: Fig. S1D). Additional filtering criteria are listed in Additional file 1: Fig. S1D. In particular, variants containing in-frame stop codons in the 30-nt downstream regions showed lower GFP intensities (Additional file 1: Fig. S1E) as they are potential NMD substrates; we removed these variants from the subsequent analyses. Furthermore, we also discarded the variants containing uATG due to their potential impact on translation initiation (Additional file 1: Fig. S1F).

Following a previous study [31], the dTomato-normalized GFP intensity of each yeast variant (*GFP*_*j*_) was calculated as the average GFP/dTomato intensity ratio among the eight bins, weighted by the proportions of its cells distributed in the eight bins (Additional file 1: Fig. S1A, Additional file 2: Table S6). The weight of variant *j* in bin *i* was estimated from *n*_*ij*_ *× P*_*i*_, where *n*_*ij*_ was the fraction of read pairs for variant *j* among all read pairs in bin *i*, and *P*_*i*_ was the proportion of cells belonging to the “gate” of bin *i* as recorded by the flow cytometer. The GFP level of variant *j* was calculated from the formula:

$${GFP}_j=\frac{\sum_{i=1}^8{G}_i\times {n}_{ij}\times {P}_i}{\sum_{i=1}^8\ {n}_{ij}\times {P}_i}$$

where *G*_*i*_ was the median GFP/dTomato ratio estimated from the collected yeast cells in bin *i* by the flow cytometer. The GFP intensity of each dATG variant is provided in Additional file 2: Table S7.

We estimated the mRNA level for each dATG variant from the ratio of read pair frequencies in the RNA-seq and DNA-seq libraries. Specifically, the read pair frequency of variant *i* in the RNA library (*R*_*i*_) or DNA-seq library (*D*_*i*_) was calculated from the fraction of read pairs derived from variant *i* among the total number of read pairs in the RNA-seq or DNA-seq library, respectively. Then, the mRNA level (abundance per cell) of variant *i* was estimated from the *R*_*i*_/*D*_*i*_ ratio. The mRNA level of each dATG variant is provided in Additional file 2: Table S7.

For the 2A-inserted dATG yeast library, the sequencing data should follow the pattern of N(12)-barcode (6 nt)-CCTCTATACTTTAACGTCAAGGAGAAAAAAATTTT-ATG-N(30)-GCAGGTCGACGGATCCCCGGGTTAATTAACA-barcode (6 nt)-N(12)-P7. And for this library, we further discarded Duo variants with a dATG located at the +4, +5, or +6 position (relative to the aATG) to ensure that all dATGs were in a strong context (AAA at positions −3 to −1 relative to the dATG). For the uATG yeast library, the “read 1” of a read pair from the sequencing data should follow the pattern of N(12)-barcode (6 nt)-CCTCTATACTTTAACGTCAAGGAGAAAAATTT-N(30)-ATG-GCAGGTCGACGGATCCCCGGGTTAATTAACA-barcode (6 nt)-N(12)-P7. Subsequent analysis procedures for these two libraries were identical to that used for the dATG yeast library. The GFP intensity and mRNA level of each variant in the 2A-inserted dATG library and in the uATG library is provided in Additional file 2: Tables S8 and S9.

### Estimation of minimum free energy (MFE) and codon adaptation index (CAI)

The mRNA secondary structure right downstream of ATG was reported to regulate translation initiation/elongation [37, 79, 80]. Since the intrinsic propensity of RNA sequences to form a secondary structure could be inferred from MFE, we estimated MFE in the 30-nt region downstream of the aATG for each variant in our dATG library using the RNAfold (http://rna.tbi.univie.ac.at/) command (RNAfold -d2 --noLP --noPS) in the package ViennaRNA [81].

The synonymous codon usage was also reported to regulate protein synthesis rate [38]. We therefore calculated CAI in the 30-nt (10-codon) region downstream of the aATG for each dATG variant, following the computational procedure described in previous studies [82, 83].

### Simulation of Brownian ratchet scanning process using a random walk model

We simulated the ratchet-and-pawl mechanism using a modified random walk model [84]. Based on a previous study [47], the triplet at the 13th–15th position of a PIC-binding mRNA fragment is inspected for complementarity to the Met-tRNAi anticodon. The PIC starts out with its 5′-trailing side at the 5′-cap and takes 1 nt per step along the mRNA. A pawl is stochastically placed onto the mRNA at the 5′-trailing side of the PIC with the probability of *p.Pawl*, and the disassociation of the pawl from the mRNA is sufficiently slow that is not considered in the model. The PIC moves with equal probabilities in the 5′–3′ and 3′–5′ directions (each 50%) unless its 5′-trailing side hits the eIF4E-cap or a pawl, in which circumstances, the PIC moves in the 5′–3′ direction with 100% probability. When an AUG is located within the 13th–15th positions covered by the PIC, the PIC may recognize the AUG in the probability of (1 − *p.Leakage*) or miss the AUG in the probability of *p.Leakage*; in the latter case, the PIC continues scanning. Note that in our simulation we assume that AUG triplets can be recognized when the PIC moves in either the 5′–3′ or 3′–5′ direction. Sometimes the PIC may recognize out-of-frame AUGs, which would activate the NMD pathway and mRNA degradation with the probability of *p.NMD*.

We simulated the PIC scanning processes on one Solo and 25 Duo variants. The Solo variant used for the computational simulation contained 50-nt sequence upstream and 50-nt sequence downstream of the aATG. The distance between the two ATGs in one of the 25 Duo variants varied within the range of 6–30 nt. When the PIC moved beyond the 50-nt downstream region (i.e., beyond the 3′-end), NMD was also activated with the probability of *p.NMD* as the PIC might encounter out-of-frame AUGs further downstream (the next three ATG triplets downstream of the variable region of *GFP* used in our experiments are all out-of-frame). We simulated the PIC scanning process for each variant (the Solo or one of the 25 Duos) 100 times and calculated the fraction of successful translation initiation events at the aATG. The protein expression level of a variant was estimated from the product of this fraction and the proportion of mRNA that did not activate the NMD pathway. We then compared this protein expression level in our simulation (Fig. 5D) with the GFP intensities measured in the experiments (variants of the two replicates combined), by calculating the residual sum of squares (RSS). Note that translation initiation at the in-frame dAUGs in Duo variants would synthesize functional proteins and would not activate NMD (Fig. 5A).

### Estimation of p.Pawl, p.Leakage, and p.NMD by the MCMC algorithms

To determine the parameters to start with in the MCMC algorithms, we screened the parameter space for *p.Pawl*, *p.Leakage*, and *p.NMD*. Specifically, we set each of the probability parameters as one of the numbers in 0.001, 0.01, 0.1, 0.2, 0.3, 0.4, 0.5, 0.6, 0.7, and 0.8 (Additional file 1: Fig. S11A). Together, there were (10^3^ =) 1000 combinational value sets. Each parameter set was individually used to simulate the Brownian ratchet scanning process, and then, the RSS was estimated from the simulated protein expression levels and observed GFP intensities in the dATG yeast library. Note that the protein expression levels of Duo variants were normalized by that of the Solo variant in both simulation and experimentation, so that the protein levels were directly comparable.

To estimate *p.Pawl*, *p.Leakage*, and *p.NMD* from the GFP intensities of the dATG variants, three hundred iterations of the MCMC simulation were performed. In each iteration, one of the three parameters was changed sequentially in the order of *p.Pawl*, *p.Leakage*, and *p.NMD*. We set the sampling window width for each parameter as 0.0004, 0.2, and 0.5, respectively, which were proximately two times the standard deviation in the ten parameter sets obtained in the initiation screening (Additional file 1: Fig. S11B). We generated a new parameter randomly based on the uniform distribution in the window defined by the sampling window width, centered at the parameter value in the previous iteration. If the boundary of the window was smaller than 0 or greater than 1, the boundary was set as 0 or 1 since all three parameters were probabilities. The new parameter set was then used to simulate the PIC scanning processes. If the RSS of the current iteration was smaller than the previous iteration, the previous set of parameters was replaced by the new set of parameters, whereas if the RSS increased, the previous set of parameters remained. After 300 iterations, the parameter set that exhibited the minimum RSS values was recorded. The net leakage rate (i.e., the fraction of PICs that eventually miss an AUG after multiple scans) was estimated from the fraction of PICs that failed to initiate translation at the aATG in the Solo variant. Based on the intrinsic relationship that the net leakage rate = (single-scan leakage rate)^number of scans^, the number of scans can be estimated as log(net leakage rate)/log(single-scan leakage rate). We repeated the simulation 30 times and obtained the distribution of the three parameters as shown in Fig. 5E and Additional file 1: Fig. S11C*.* The average value and the standard errors (SE) were estimated from the 30 sets of optimized parameters by the MCMC algorithms (Fig. 5F).

To estimate the *p.Leakage* for the ATG in the A, G, C, and T contexts individually, we performed the MCMC algorithms with 100 additional iterations, during which we estimated the RSS between the simulated protein expression levels and the GFP intensities measured for the Solo variants that with A, G, C, or T at the −3 position in the dATG library. In the simulation, we fixed the *p.Pawl* and *p.NMD* values as acquired above (*p.Pawl* = 0.001 and *p.NMD* = 0.62). The MCMC algorithms were performed 30 times, and the average *p.Leakage* for each of the four ATG contexts was estimated from the average value of the 30 MCMC chains (Fig. 5G).

We also used the MCMC algorithms to estimate numerical approximations for the parameters in the Brownian ratchet scanning model from the GFP intensities of the uATG variants. The same procedures (including initial parameter sets and sample window width) as the analyses of the dATG library were used.

### Detection of out-of-frame dAUGs in eukaryotic and prokaryotic genomes

The coding sequences of the budding yeast (*Saccharomyces cerevisiae*, R64-1-1) and human (*Homo sapiens*, GRCh38) genes were downloaded from the Ensembl database [85] using BioMart [86], and the sequence of the main transcript of each gene defined in the Ensembl database were used for the subsequent analyses. A gene was discarded if its annotated initiation codon was not ATG or if it was shorter than 45 nt in length. The numbers of genes used for the subsequent analyses were 6564 (yeast) and 19,496 (humans). The genes harboring dATGs located at each position within the 45-nt region downstream of the aATG were counted in each genome.

As a negative control, we also retrieved the coding sequences of two prokaryotic genomes [87], *Escherichia coli* (GenBank: NZ_CP027599.1) and *Bacillus subtilis* (GenBank: NC_000964.3), where no scanning mechanism is required for recognition of the initiation codon. We applied the same set of criteria as described above for yeast and humans and identified 5113 (*E. coli*) and 3282 (*B. subtilis)* genes for the subsequent analyses.

The distribution of dAUGs in all three possible frames was also analyzed in subsets of genes based on their gene expression level (in yeast) or the number of tissues expressing the gene (in humans). The yeast gene expression levels and transcription rates were retrieved from previous studies [88, 89] and the transcript expression levels for each human gene in 54 tissues were retrieved from the GTEx database (dbGaP accession number phs000424.v8.p2) on 09/13/2022. The number of tissues that a human transcript is expressed (transcript per million greater than or equal to 1) was counted.

## Supplementary Information

Additional file 1: Fig. S1. High-throughput quantification of GFP intensities by FACS-seq. Fig. S2. Frame- and context-dependent inhibitory effects on protein synthesis by proximal dAUGs. Fig. S3. Codon adaptation index (CAI) and minimum free energy (MFE) do not significantly vary among Duo variants that contain dATGs at different positions. Fig. S4. The mRNA levels of dual-frame reporters and their respective controls measured by quantitative PCR. Fig. S5. High-throughput measurement of mRNA levels for dATG variants. Fig. S6. GFP intensities of dATG variants in the background of *upf1Δ*. Fig. S7. GFP intensity and mRNA level of the dATG variants in the 2A-inserted library. Fig. S8. Scatter plots showing GFP intensity and mRNA level in two biological replicates for yeast libraries. Fig. S9. GFP intensity and mRNA level for uATG variants in the background of *hoΔ*. Fig. S10. GFP intensity and mRNA level for uATG variants in the background of *upf1Δ*. Fig. S11. The optimization of the parameters in the Brownian ratchet scanning model using the MCMC algorithms. Fig. S12. The observed GFP intensities of dATG variants in FACS-seq experiments and the simulated GFP intensities under the Brownian ratchet scanning model. Fig. S13. Numbers of genes that harbor frame 0 or frame +2 dATGs at individual positions downstream of the aATG in the yeast or human genomes. Fig. S14. Numbers of genes harboring out-of-frame dATG for highly/broadly and lowly/narrowly expressed genes.

Additional file 2: Table S1. Primers used in this study. Table S2. Doped nucleotide oligos used to construct yeast libraries. Table S3. Indices and barcodes used for Illumina sequencing library. Table S4. Sequences of the dual-fluorescence and dual-frame reporter. Table S5. Number of sequencing reads and variants in each sample. Table S6. Detailed information on individual bins in the FACS-seq experiment. Table S7. Detailed information on dATG variants in this study. Table S8. Detailed information on 2A-inserted dATG variants in this study. Table S9. Detailed information on uATG variants in this study.

Additional file 3. Peer review history.

## Data Availability

The reference sequence and reference annotation of the *Saccharomyces cerevisiae* genome (R64-1-1) and the *Homo sapiens* genome (GRCh38.p13) were downloaded from Ensembl database [90]. The reference sequence and gene annotation of *Escherichia coli* genome (NZ_CP027599.1) and *Bacillus subtilis* genome (NC_000964.3) were retrieved from NCBI database [91]. The expression levels and transcription rates of yeast genes were retrieved from previous studies [88, 89]. The expression levels of human transcripts in 54 tissues were obtained from the GTEx Portal (dbGaP accession number phs000424.v8.p2) on 09/13/2022. The high-throughput sequencing data of ribosome protected fragments were retrieved from a previous Ribo-seq study [92]. The high-throughput sequencing data of FACS-seq, RNA-seq, and DNA-seq have been deposited at Genome Sequence Archive [93] under the accession number CRA005456 [94]. Codes to analyze the data are available at Zenodo [95], under the terms of the Creative Commons Attribution 4.0 license.
