# Supplementary material for: Distance-dependent inhibition of translation initiation by downstream out-of-frame AUGs is consistent with a Brownian ratchet process of ribosome scanning
Source: Genome Biol. 2022 Dec 12;23:254. doi: 10.1186/s13059-022-02829-1 (PMC9743702; doi:10.1186/s13059-022-02829-1)
Supplement: Supplementary file 1 — Additional file 1: Fig. S1. High-throughput quantification of GFP intensities by FACS-seq. Fig. S2. Frame- and context-dependent inhibitory effects on protein synthesis by proximal dAUGs. Fig. S3. Codon adaptation index (CAI) and minimum free energy (MFE) do not significantly vary among Duo variants that contain dATGs at different positions. Fig. S4. The mRNA levels of dual-frame reporters and their respective controls measured by quantitative PCR. Fig. S5. High-throughput measurement of mRNA levels for dATG variants. Fig. S6. GFP intensities of dATG variants in the background of upf1Δ. Fig. S7. GFP intensity and mRNA level of the dATG variants in the 2A-inserted library. Fig. S8. Scatter plots showing GFP intensity and mRNA level in two biological replicates for yeast libraries. Fig. S9. GFP intensity and mRNA level for uATG variants in the background of hoΔ. Fig. S10. GFP intensity and mRNA level for uATG variants in the background of upf1Δ. Fig. S11. The optimization of the parameters in the Brownian ratchet scanning model using the MCMC algorithms. Fig. S12. The observed GFP intensities of dATG variants in FACS-seq experiments and the simulated GFP intensities under the Brownian ratchet scanning model. Fig. S13. Numbers of genes that harbor frame 0 or frame +2 dATGs at individual positions downstream of the aATG in the yeast or human genomes. Fig. S14. Numbers of genes harboring out-of-frame dATG for highly/broadly and lowly/narrowly expressed genes. [file 13059_2022_2829_MOESM1_ESM.docx]

**Additional file 1: Supplemental figures for**

**Distance-dependent inhibition of translation initiation by downstream out-of-frame AUGs is consistent with a Brownian ratchet process of ribosome scanning**

K. Li, J. Kong, S. Zhang, T. Zhao, and W. Qian


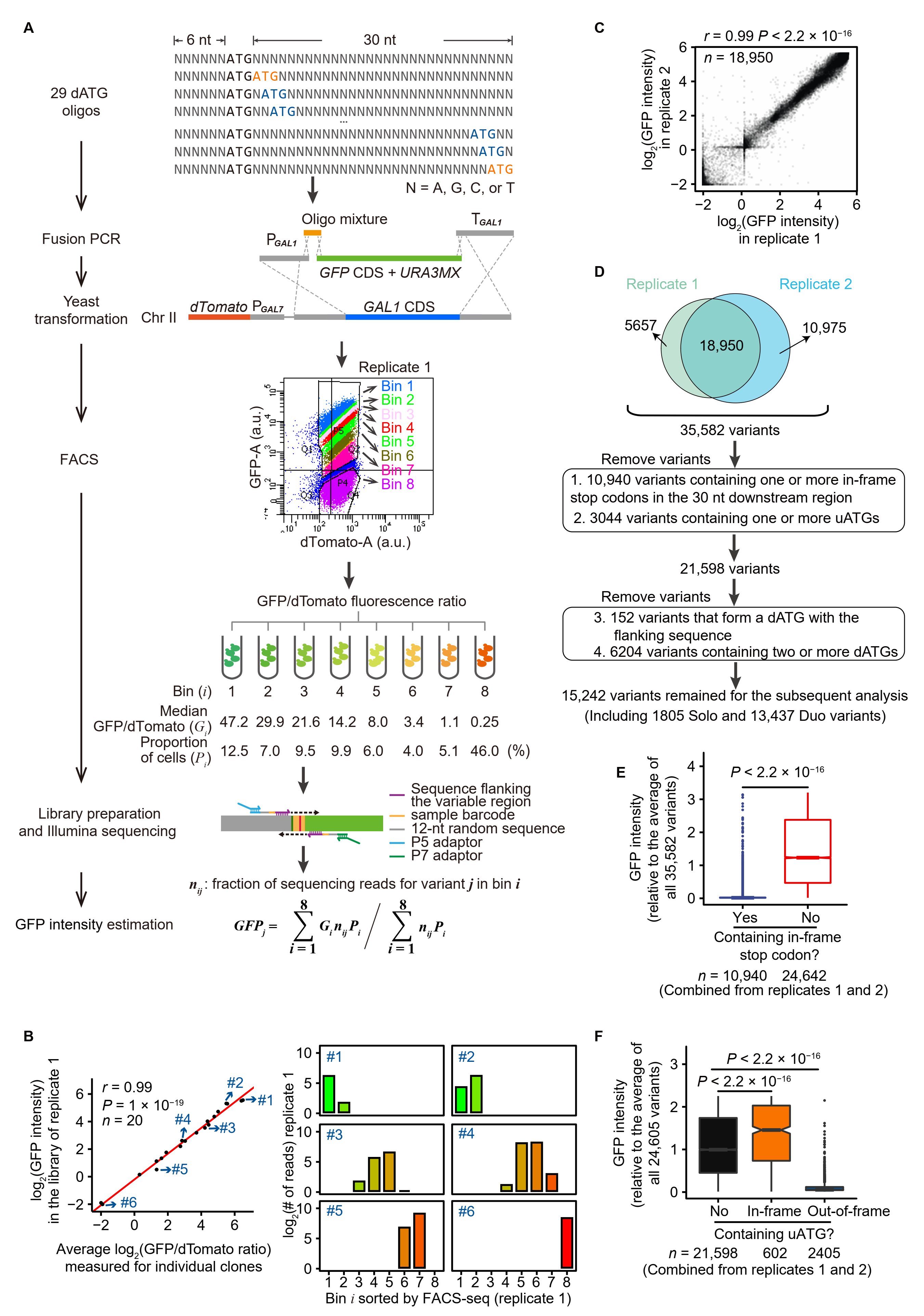


**Fig. S1. High-throughput quantification of GFP intensities by FACS-seq.**

(A) A schematic shows FACS-seq procedure to estimate GFP intensity. The scatter plot shows the GFP and dTomato intensities (the total pulse area) of individual yeast cells and the “gates” for sorting yeast cells into eight bins by FACS.

(B) A scatter plot shows the comparison of the GFP intensities of 20 randomly chosen yeast variants measured *en masse* by FACS-seq (*y*-axis) vs. measured individually by flow cytometry (*x*-axis). Specifically, we randomly isolated 20 clones from the library and for each clone, we estimated the GFP/dTomato fluorescence ratio for >19,000 cells; the average GFP/dTomato ratio is shown on the *x*-axis. The red line represents the standard major axis. Pearson’s correlation coefficient *r* and the corresponding *P* value are also shown. The six histograms exemplify the distribution of read numbers among the eight bins in the FACS-seq experiment. Overall, these results confirm the accuracy of our FACS-seq experiments in estimating GFP intensity for the dATG variants in the yeast library.

(C) A scatter plot shows GFP intensities measured in two biological replicates for yeast dATG variants. Pearson’s correlation coefficient *r* and the corresponding *P* value are shown.

(D) The flowchart shows the number of variants passing individual computational filters. The variants that contain only one ATG in the synthesized 39-nt region (*i.e.*, the designed aATG) are denoted as “Solo” variants, and the variants that contain one additional ATG in the 30-nt downstream region (*i.e.*, the dATG) are denoted as “Duo” variants.

(E) A boxplot shows GFP intensities for variants with or without in-frame stop codons in the 30-nt variable region. *P* value was given by the Mann-Whitney *U* test.

(F) A boxplot shows GFP intensities for variants without uAUGs, with at least one in-frame uAUG, and with an out-of-frame uAUG. *P* values were given by the Mann-Whitney *U* tests.


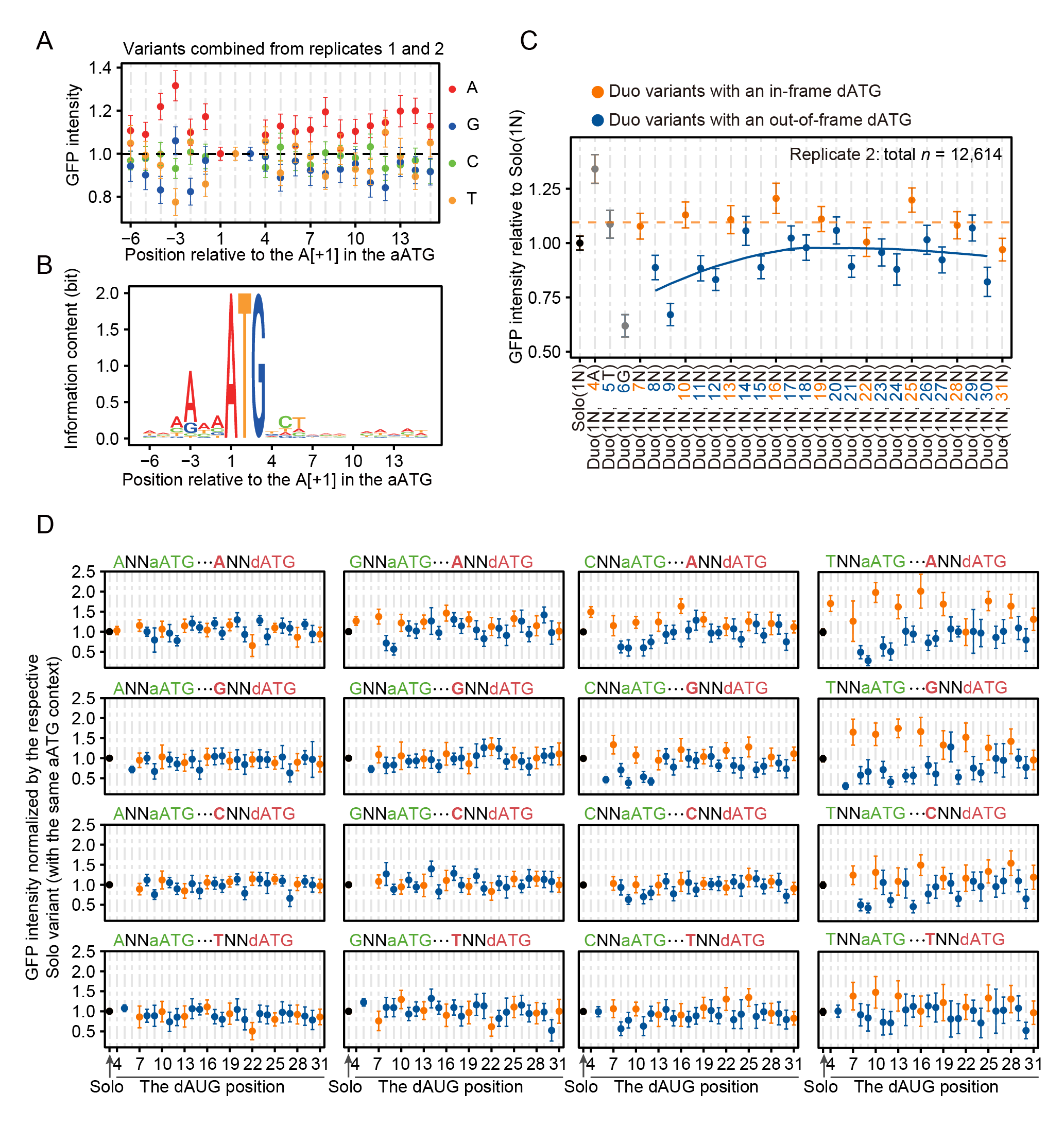


**Fig. S2. Frame- and context-dependent inhibitory effects on protein synthesis by proximal dAUGs.**

(A) The average GFP intensity of Solo variants grouped by the nucleotide at each position. Variants were aligned according to their aATGs. The GFP intensity for each Solo variant was normalized by the average GFP intensity of all the 1805 Solo variants. Error bars represent the 95% confidence intervals.

(B) Sequence logo around the aATG among the 500 genes in the yeast genome that show the highest protein synthesis rate (reflected by the abundance of ribosome protected fragments per unit gene length in a ribosome profiling dataset generated by Ingolia *et al*. in 2009). The sequence logo was generated with R package “ggseqlogo”. Note that in the upstream region of the aATG, the GFP intensities for the four nucleotides shown in Fig. S2A (which reflects the relative capability in retaining PIC by an aAUG during PIC scanning) are largely consistent with the sequence logo shown here: “A” is over-represented from positions −6 to −1, with the −3 position being the most significant. We speculate that the discrepancy between Fig. S2A and Fig. S2B downstream of the aATG is related to the coding potential of these sequences. That is, downstream adenines may be favored for AUG recognition by the PIC, as suggested by the GFP reporter data in Fig. S2A but are not necessarily favored during sequence evolution of endogenous genes due to natural selection for their respective protein functions (thereby amino-acid sequences).

(C) The average GFP intensities and the 95% confidence intervals of dATG variants measured in biological replicate 2.

(D) The average GFP intensities and the 95% confidence intervals of dATG variants, grouped jointly by the nucleotides at the −3 position of aATG and dATG. Variants from both biological replicates were combined.


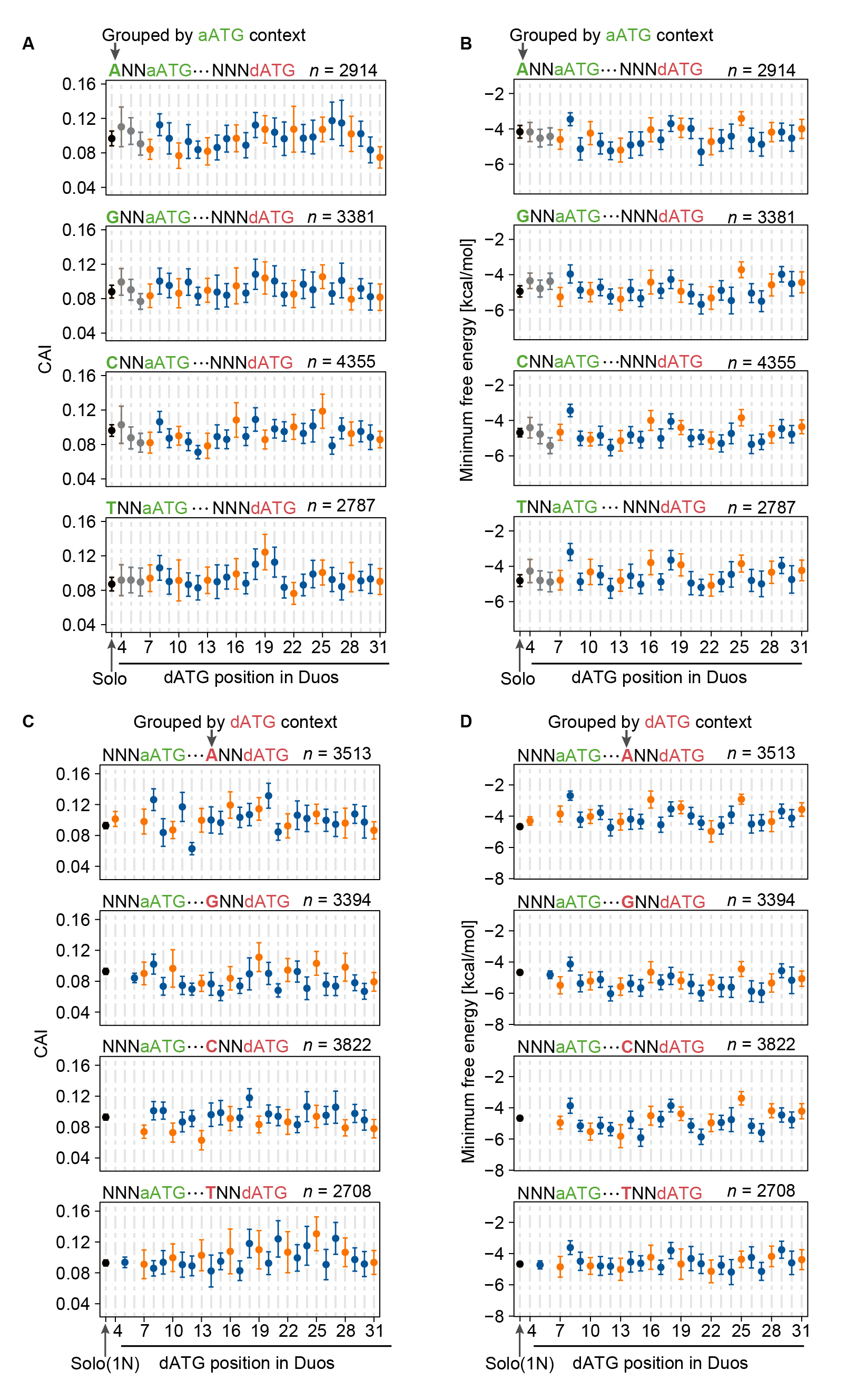


**Fig. S3. Codon adaptation index (CAI) and minimum free energy (MFE) do not significantly vary among Duo variants that contain dATGs at different positions.**

Duo variants were grouped by the nucleotide at the −3 position of the aATG (A–B) or dATG (C–D). Error bars represent the 95% confidence intervals, and the total number of Duo variants used in each panel (*n*) is shown on the top. A higher CAI value means the tendency to use more preferred codons, and a higher MFE value means more unstable mRNA secondary structure.


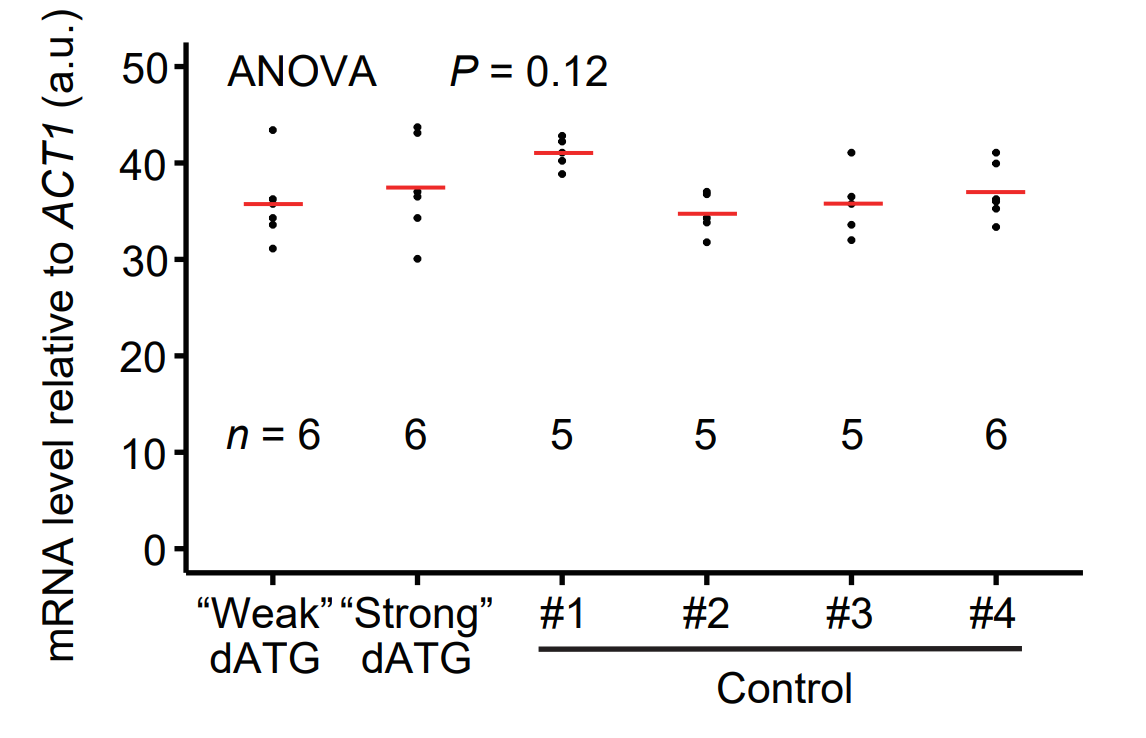


**Fig. S4. The mRNA levels of dual-frame reporters and their respective controls measured by quantitative PCR.**

The reported mRNA levels were normalized by the mRNA level of *ACT1* in the respective yeast strain. *P* value was given by the one-way analysis of variance.


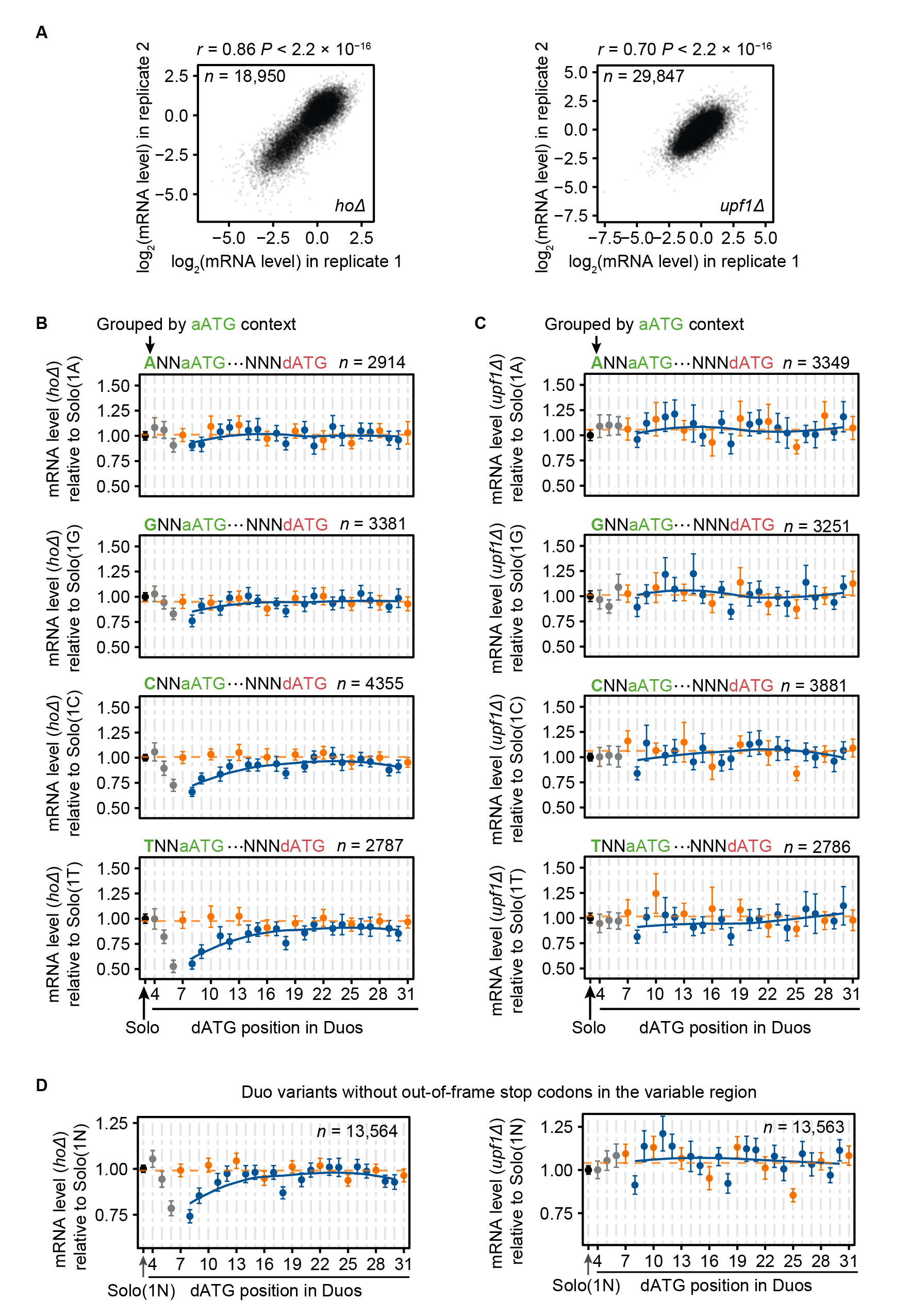


**Fig. S5. High-throughput measurement of mRNA levels for dATG variants.**

(A) Scatter plots showing mRNA levels of the dATG variants measured in two biological replicates, in the genetic background of *hoΔ* or *upf1Δ*. Pearson’s correlation coefficients *r* and the corresponding *P* values are shown.

(B–C) The average mRNA levels and the 95% confidence intervals of dATG variants, in the background of *hoΔ* (B) and *upf1Δ* (C), grouped by the nucleotide at the −3 position of the aATG.

(D) The average mRNA levels and the 95% confidence intervals of dATG variants that do not contain stop codons in the variable region that can terminate translation from dATGs, in the *hoΔ* or *upf1Δ* background.


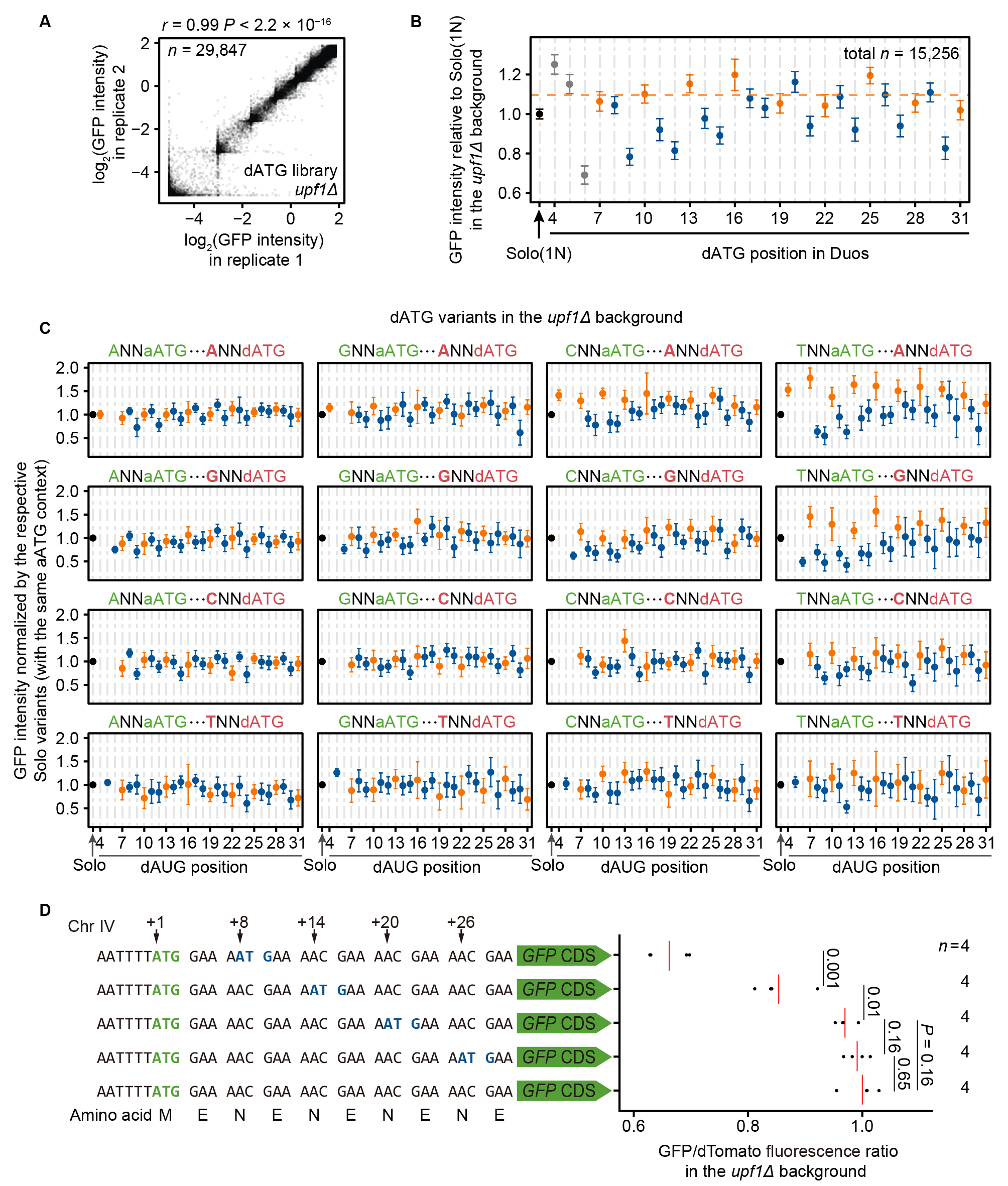


**Fig. S6. GFP intensities of dATG variants in the background of *upf1Δ*.**

(A) A scatter plot showing GFP intensity in two biological replicates for dATG variants in the background of *upf1Δ*.

(B) The average GFP intensities (dots) and the 95% confidence intervals (error bars) of Duo variants in the background of *upf1Δ*.

(C) The average GFP intensities and the 95% confidence intervals of dATG variants, in the background of *upf1Δ*, grouped jointly by the nucleotide at the −3 position of the aATG and dATG.

(D) GFP/dTomato fluorescence ratios measured for the variants used in Fig. 1F, in the background of *upf1Δ*.
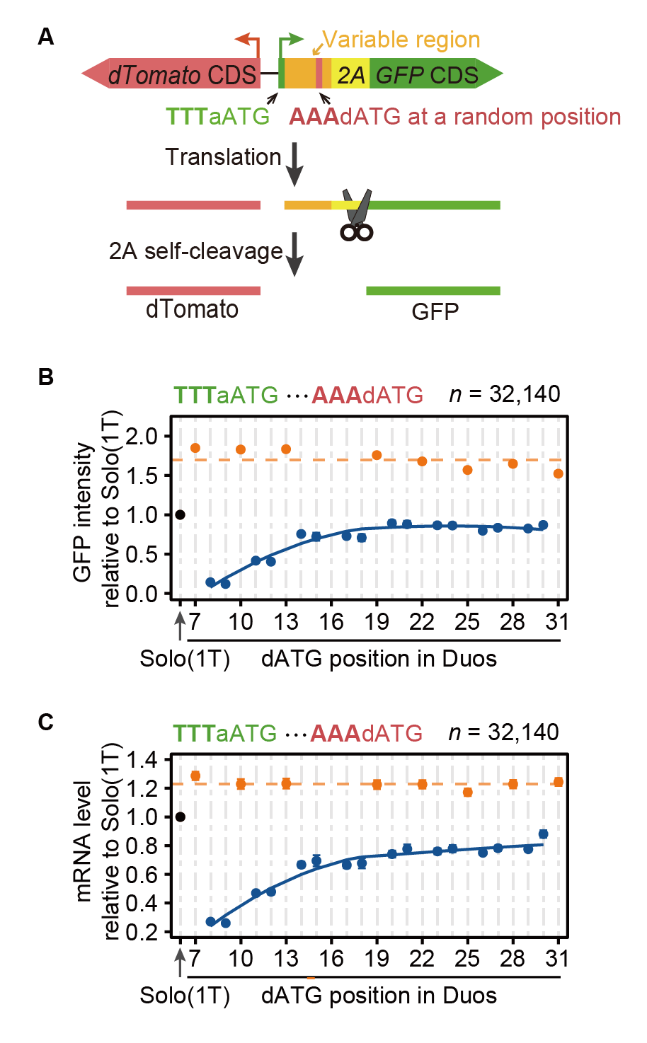


**Fig. S7. GFP intensity and mRNA level of the dATG variants in the 2A-inserted library.**

(A) DNA sequence encoding a 2A self-cleaving peptide was inserted between the 30-nt variable region and the GFP coding sequences, and therefore, amino-acid differences introduced to the N-terminus of GFP were removed after the 2A self-cleavage. We fixed the sequence context of the aATG and dATG to TTT and AAA (at positions from −3 to −1 relative to the ATG), respectively, when constructing the 2A-inserted dATG library.

(B–C) The GFP intensity (B) and mRNA level (C) of Duo variants with the dATG at locations ranging from +7 to +31 in the 2A-inserted dATG library. The missing data for the Duo variants with the dATG at position +15 was caused by the relatively small number of variants detected (*n* = 3). Similar to Fig. 1E, dots represent the mean and error bars show the 95% confidence intervals.


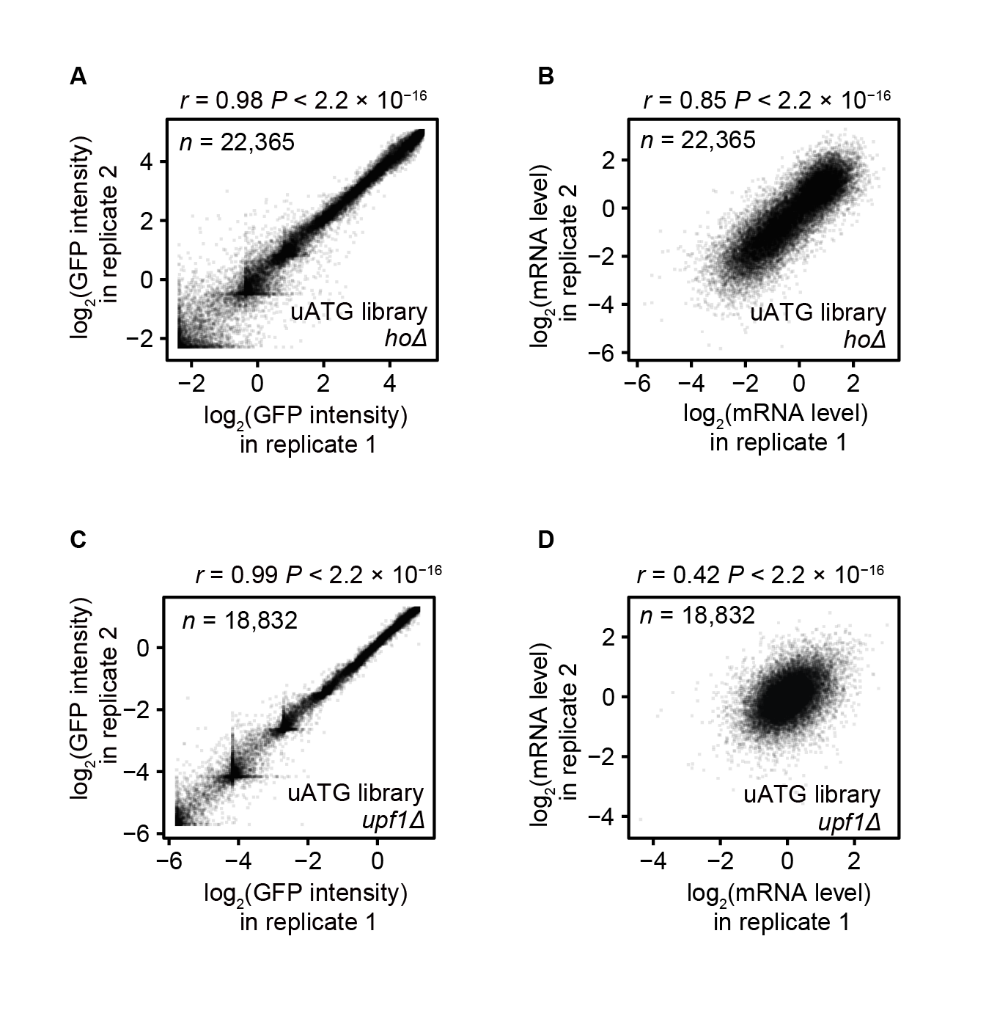


**Fig. S8. Scatter plots showing GFP intensity and mRNA level in two biological replicates for uATG yeast libraries.**


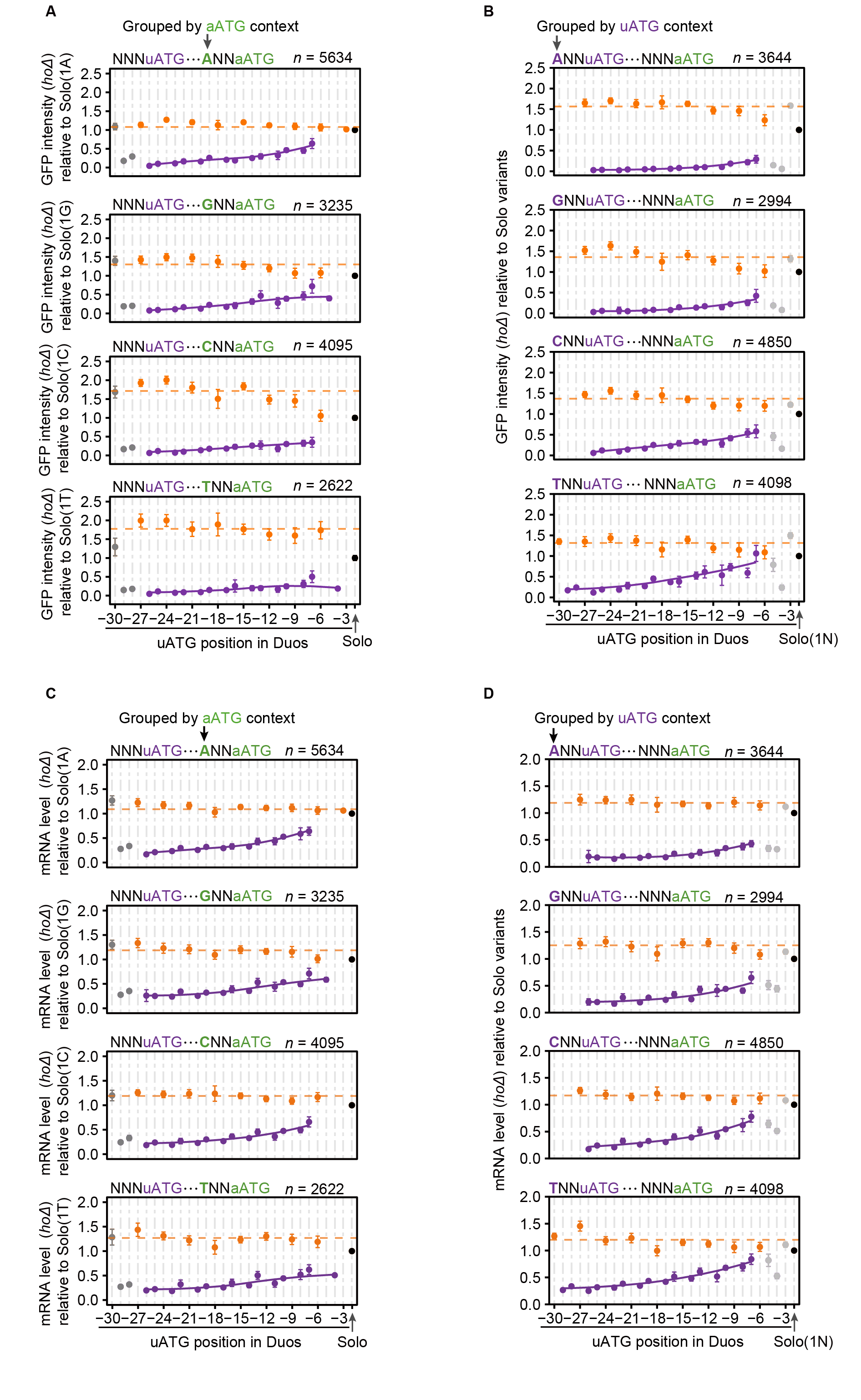


**Fig. S9. GFP intensity and mRNA level for uATG variants in the background of *hoΔ*.** Similar to Fig. 2A–B, dots represent the mean and error bars show the 95% confidence intervals.


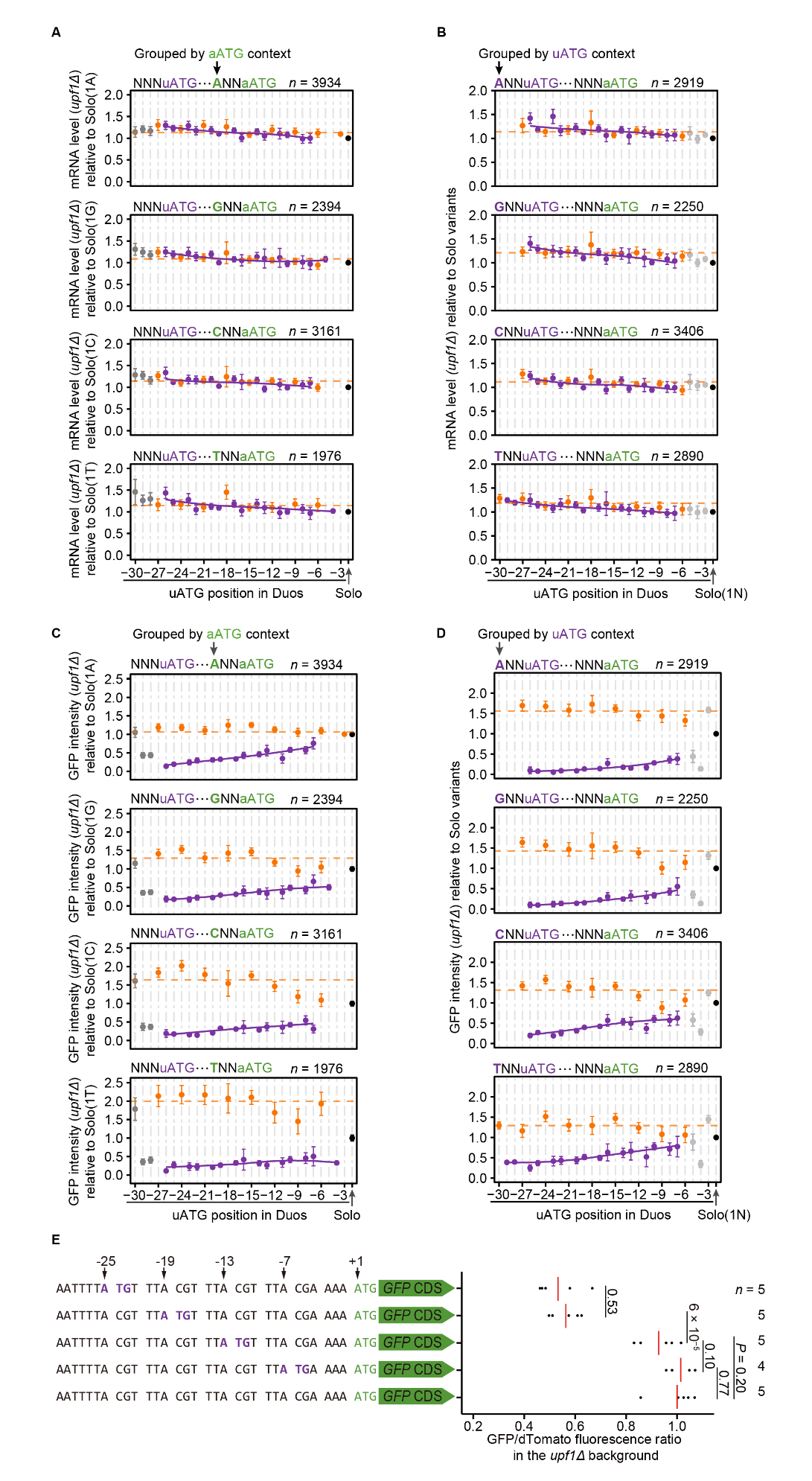


**Fig. S10. GFP intensity and mRNA level for uATG variants in the background of *upf1Δ*.**

(A–D) GFP intensity and mRNA level for the variants in the uATG library in the background of *upf1Δ*. Dots represent the mean and error bars show the 95% confidence intervals.

(E) GFP/dTomato fluorescence ratios measured for the variants used in Fig. 4D, in the background of *upf1Δ*.


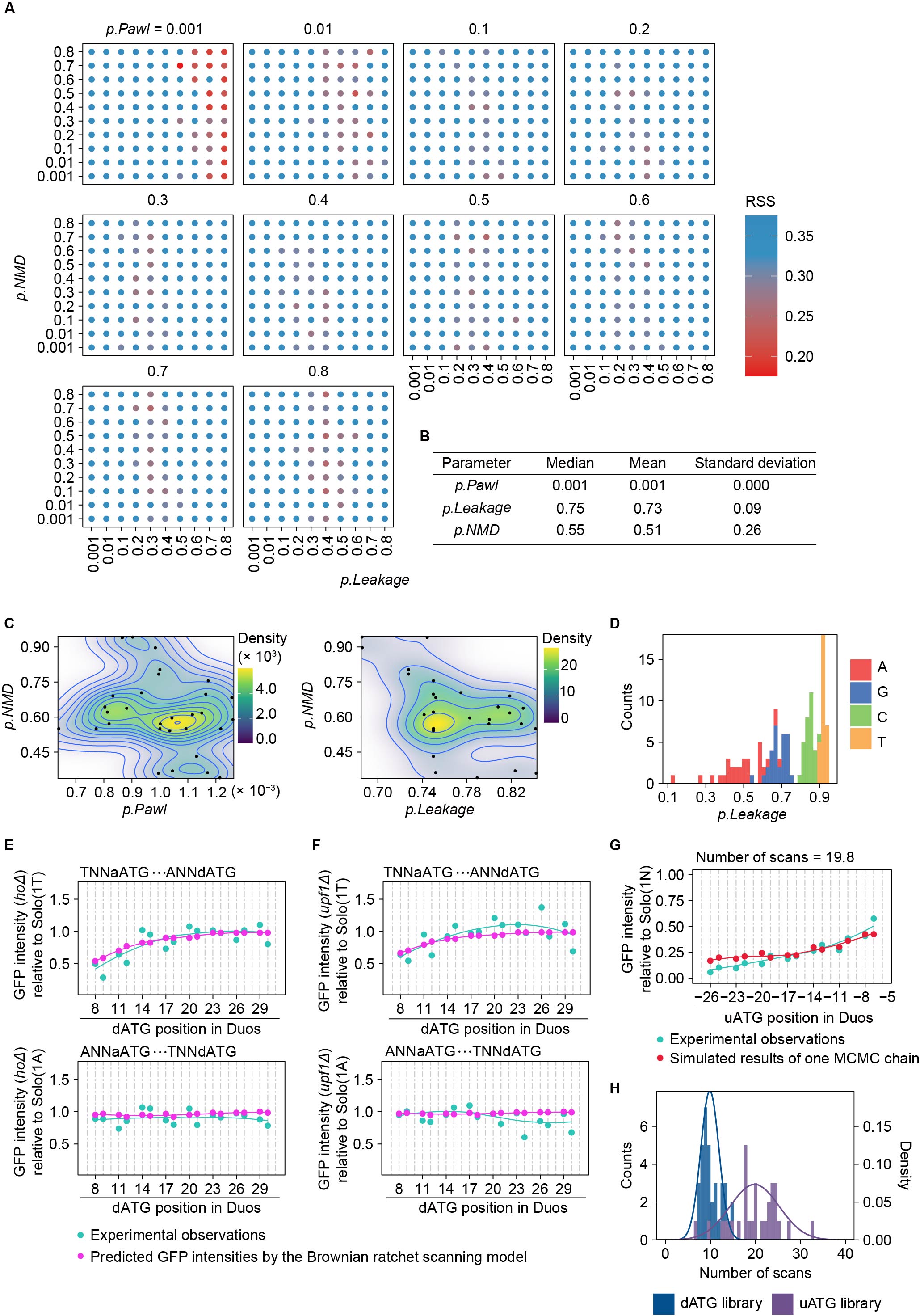


**Fig. S11. The optimization of the parameters in the Brownian ratchet scanning model using the MCMC algorithms.**

(A) The residual sum of squares (RSS, values shown in color) estimated from the observed GFP intensities in the FACS-seq experiments on the dATG library and the simulated protein expression level, using each of the 1000 parameter sets (10 values for *p.Pawl* × 10 values for *p.Leakage* × 10 values for *p.NMD*).

(B) A table shows the summary statistics for *p.Pawl*, *p.Leakage*, and *p.NMD*, estimated from ten (out of the 1000) parameter sets with the smallest RSS. The standard deviation of *p.Pawl* was equal to zero due to the absence of variation among the ten *p.Pawl* values.

(C) Similar to Fig. 5E, two additional two-dimensional density plots show distributions of the outcome parameter values among the 30 MCMC chains.

(D) A stacked histogram plot shows the distributions of the outcome values of *p.Leakage* for ATGs in the A-, G-, C-, and T-contexts, respectively, among 30 MCMC chains.

(E, F) The observed and predicted GFP intensities for out-of-frame dATG variants, in the genetic background of *hoΔ* (E) or *upf1Δ* (F). Here, we predicted GFP intensities under the Brownian ratchet scanning model using the context-dependent *p.Leakage* estimated from Solo variants, for a subset of Duo variants that harbor aATG in a weak context and dATG in a strong context, or the other way around. Specific parameters used: *p.Leakage* = 0.92 for ATGs in the T-context and *p.Leakage* = 0.49 for ATGs in the A-context, *p.Pawl* = 0.001, and *p.NMD* = 0.62 in the *hoΔ* background. When simulating in the *upf1Δ* background, *p.NMD* was set to 0.

(G) The observed GFP intensities of out-of-frame uATG variants in the yeast experiments (two replicates combined) and the simulated GFP intensities using one of the 30 sets of optimized parameters by the MCMC algorithms.

(H) A stacked histogram plot shows the distributions of the estimated number of scans (for each triplet, before the PIC eventually migrated to downstream regions of the mRNA). These numbers were estimated by the MCMC algorithms under the Brownian ratchet scanning model, using either the experimental data of dATG or uATG variants. The curves show the normal distributions with means and standard deviations of the number of scans estimated from the dATG and uATG data.


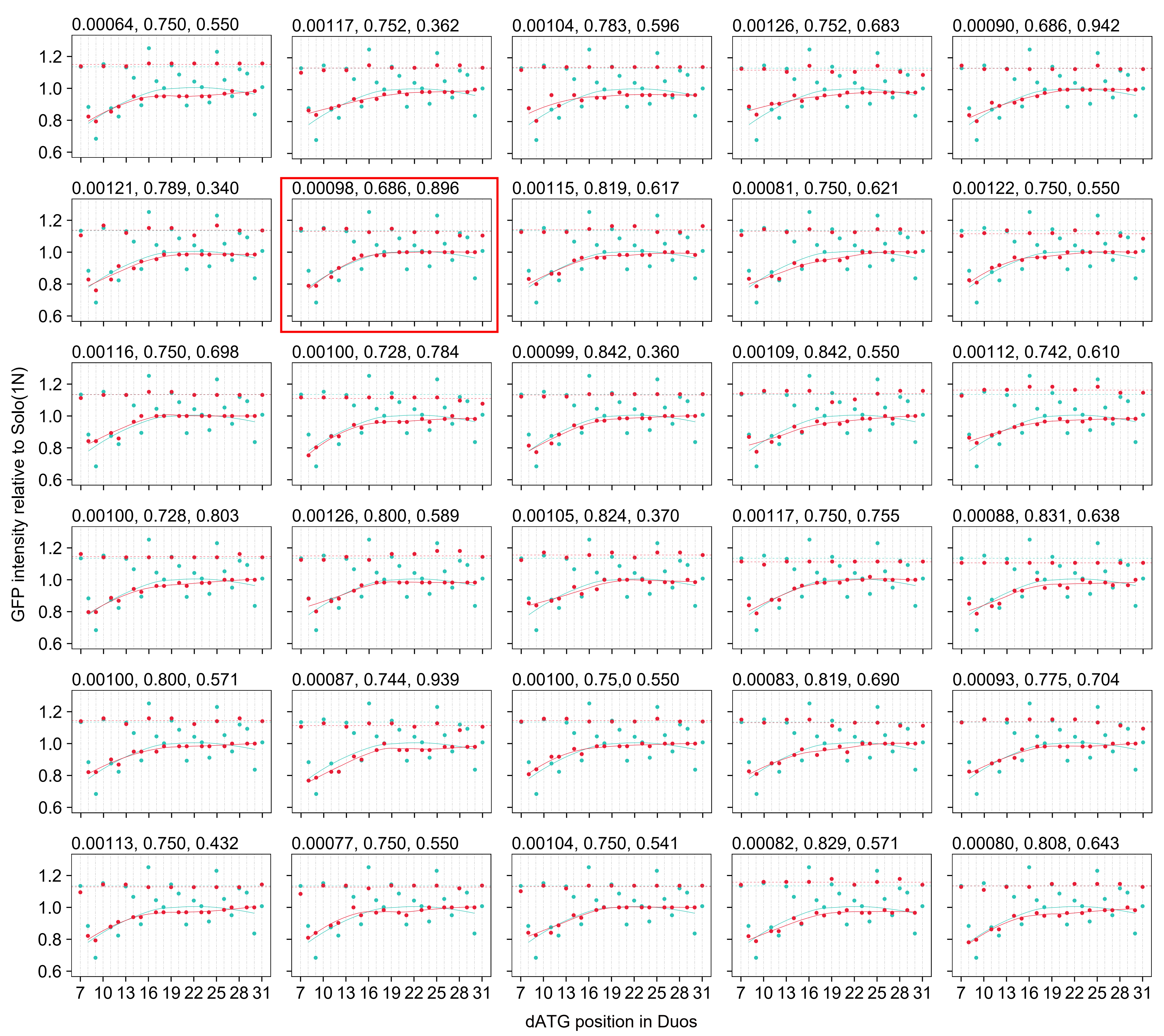


**Fig. S12. The observed GFP intensities of dATG variants in FACS-seq experiments and the simulated GFP intensities under the Brownian ratchet scanning model.**

Similar to Fig. 5D, plots show the results of all 30 MCMC chains. The parameter values optimized by each MCMC chain are shown on the top of each panel, in the order of *p.Pawl*, *p.Leakage*, and *p.NMD*. The result shown in Fig. 5D is indicated by the red box.


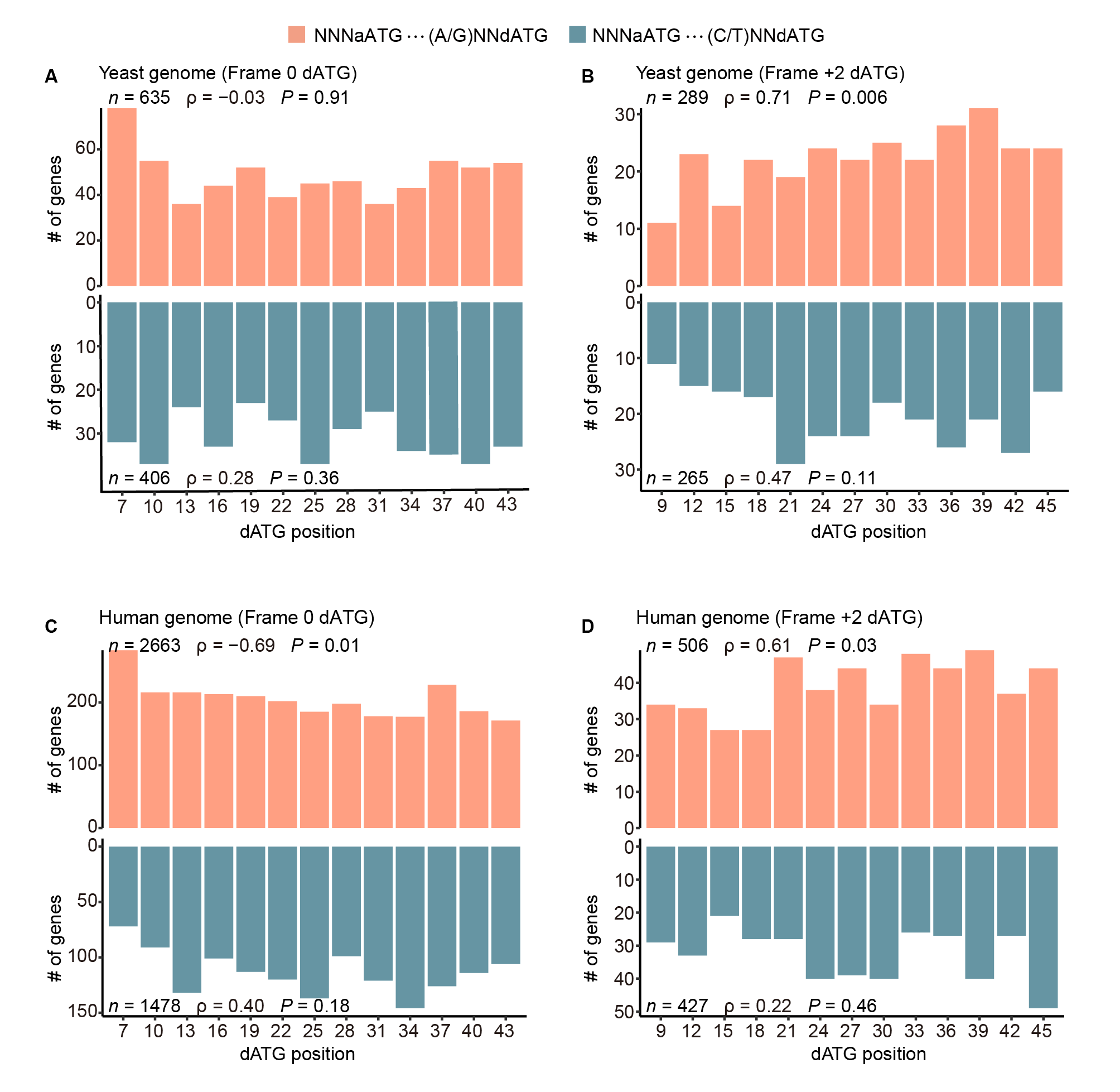


**Fig. S13. Numbers of genes that harbor frame 0 or frame +2 dATGs at individual positions downstream of the aATG in the yeast or human genomes.**

Similar to Fig. 6B, the color of bars shows the context of dATGs. Spearman’s correlation coefficients ρ and the corresponding *P* values are shown.


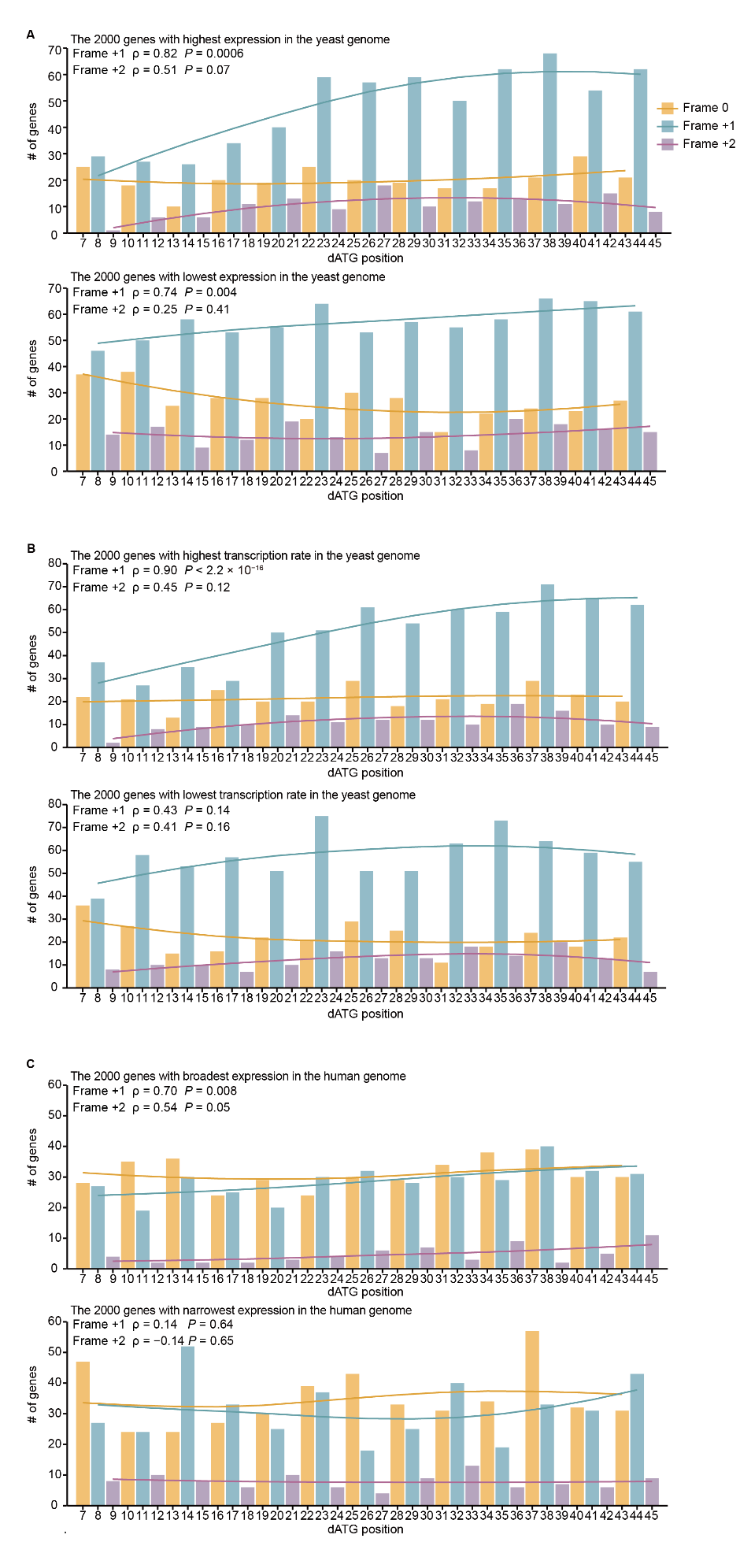


**Fig. S14. Numbers of genes harboring out-of-frame dATG for highly/broadly and lowly/narrowly expressed genes.**

Similar to Fig. 6A, the numbers of genes harboring out-of-frame dATG among 2000 highly and 2000 lowly expressed genes in the yeast genome (A), among 2000 highly and 2000 lowly transcribed genes in the yeast genome (B), and among 2000 broadly and 2000 narrowly expressed genes in the human genome (C). Spearman’s correlation coefficients (ρ) and corresponding *P* values are shown. Note that although the *P* values for highly and lowly expressed yeast genes were similar for the frame +1 dATGs, highly expressed genes exhibited stronger depletion, as indicated by the steeper curve.
