# Supplementary material for: Distance-dependent inhibition of translation initiation by downstream out-of-frame AUGs is consistent with a Brownian ratchet process of ribosome scanning
Source: Genome Biol. 2022 Dec 12;23:254. doi: 10.1186/s13059-022-02829-1 (PMC9743702; doi:10.1186/s13059-022-02829-1)
Supplement: Supplementary file 3 — Additional file 3. Peer review history. [file 13059_2022_2829_MOESM3_ESM.docx]

**Review history**

**First round of review**

**Reviewer 1**

The manuscript entitled "Distance-dependent inhibition of translation initiation by downstream out-of-frame AUGs reveals that ribosome scans in a Brownian ratchet process" by Li et al. describes a really nice study aimed at solving an old conundrum in the field of eukaryotic translation initiation.
Since 1970s, when M.Kozak established her scanning model, a number of important questions arose regarding mechanistic aspects of ribosomal scanning. Among them, one of the most intriguing ones is the question of how the 5'-to-3' directionality of scanning is provided and whether it is unidirectional or includes diffusional wandering back and forth along the 5' untranslated region. A few classic (mostly in vitro) studies were devoted to solving this puzzle, leading to a concept of net unidirectional movement instead of "random wandering" (stochastic bi-directional movement). However, there is yet a lack of understanding whether scanning is strictly unidirectional (governed by, say, an RNA helicase constantly fed by ATP) or includes some limited back-and-forth movements (governed by Brownian motion and directed from 5'- to 3' by an occasional pawl placement - in this case, ATP is needed for the pawl placement). This is actually very important issue, as these two modes should lead to different patterns of AUG recognition. I would note, however, that most of recent studies in the field, unfortunately, have been based on the premise that scanning is strictly unidirectional, although no evidence has been provided for this model.

In this regard, the study by Prof. Qian's group is very important, as it clearly shows that the 43S pre-initiation complex uses limited bi-directional oscillations (governed by Brownian motion) instead of the strictly unidirectional movement. To reveal this, the authors used systems biology approaches, including a reporter library screen, a genome-wide mRNA sequence analysis and analysis of gene expression data from The Cancer Genome Atlas. Importantly, they also built a mathematical model, which allowed them, taking into account their experimental data, revealing quantitative parameters of 43S PIC scanning.

For those who are well acquainted with the literature on this issue, this is a long-expected discovery, as some fragmentary indications of this could be found in the literature before (carefully cited by the authors: i.e. Matsuda and Dreher, 2006; Williams and Lamb, 1989; Terenin et al., 2016; Gu et al., 2021). However, this has never been clearly stated and confirmed by elegant experiments. Thus, to my opinion, this study should be considered as one of the brightest event in the field during the last few years. I would also note an inventive experimental design and the fact that the authors inspected many aspects of their finding and extensively used good positive and negative controls in their experiments, as well as a careful presentation and very competent discussion of the findings. That's why I enthusiastically recommend this manuscript to be published in Genome Biology, once the authors address a few major and several important minor issues listed below.

Major points

1. Lines 269-277 and Fig. 1F: This experiment clearly lacks the analysis of a control construct having no dAUG. It is strange that the authors did not add these data (all the more so that the corresponding construct was indeed present in a similar experiment in HeLa cells).

2. Please double check the data, their description, and the protocol details for the experiment described in lines 174-183 (Fig.S1B). I'm really quite amazed that the authors were so lucky to obtain almost ideal correspondence between a GFP/dTomato ratio for a number of variants in the library (where the GFP/dTomato ratio is actually an abstract mathematical estimation, i.e. the result of a complex calculation based on distribution of a particular variant between eight bins (the variant frequencies) obtained after quantification of Illumina reads) and a real GFP/dTomato ratio obtained for the same variants individually (in this case directly measured by flow cytometry). It could be that in the second case the GFP/dTomato ratio was also a result of some calculation based on distribution between some bins (but which frequencies? - there was no Illumina sequencing here). Please clarify this issue.

3. It would be useful to analyze effects of dAUGs in the upf1Δ strain, to get an estimation of a "clear" dAUG effects on translation initiation without impact of NMD-related mRNA stability. Of course, it is not necessary to repeat en masse analysis in this strain, but it would not be difficult to perform an additional small-scale experiment with a few particular reporter constructs like those shown in Fig. 1F.


Minor points

Line 74-76: [Berthelot et al., 2004] was clearly not the only or the first paper where "the insertion of a strong mRNA secondary structure between the 5′-cap and the aAUG" was shown to "prevent translation initiation" (either in yeast or in other eukaryotes). Some classic review (by M.Kozak or A.Hinnebusch, e.g. PubMedID 16213112, 27313038 or 24499181) could be cited here; if you prefer a research article, it should be probably PMID 3458245, but I suggest using a review instead.

Lines 137-138: Studies that "have observed reduced protein abundance upon the addition of an out-of-frame uATG" are not limited to these three, of course - there have been multiple studies showing that, published since 1970s. Please specify this issue (e.g. systems studies or smth. like that).

Line 156, "resulting in a representative library": the library used by the authors was not representative. They were actually able to use just a few thousands of constructs in their study, while the complete library, to be indeed representative (at least 1 molecule per variant), should include 4e36 molecules, i.e. ~10e21 ones (which is impossible, as such DNA oligos have a weight of ~0.1 kg)! This is not a problem, as they actually had enough dAUG-containing variants to calculate all they needed, but a reader should be informed that the variants taken into analysis were just a little of the huge number of all possible variants.

Lines 206-213: the prevailing importance of "A" in the -3 position, as the only recognizable feature of "good" AUG context in yeast, was described by Hamilton et al., 1987. It should be mentioned here.

Lines 263-264: It is actually a delusion that IRES is a "consensus sequence", so "IRES similarity" makes no sense. All well approved IRESs are long and complex RNA segments, usually having no extensive similarity at the level of nucleotide sequence but instead belonging to a few types at the level of secondary and tertiary structure. You can find the corresponding discussion elsewhere (e.g. PMID 27853833, 23378589, 16314320, 34565312). I recommend removing this analysis form the text (as well as from Fig. S3), so as not to compromise this high-competent study. Please also check whether citing Kudla et al., 2009 (a study of coding-sequence determinants of gene expression in E.coli) is relevant here.

Lines 294-305 and Fig. 2A: I suggest using normalization to results obtained with the corresponding particular Solo constructs: Solo(1A/1G/1C/1T). To my opinion, using the normalization to Solo(1N) is misleading, as a reader may not understand why in the two top subpanels addition of dATGs "stimulated" translation (it was actually the good aAUG context that stimulated, but at first sight it is unclear).

Line 308: "an overlapping dual-fluorescent reporter": please re-phrase.

Line 342 and elsewhere: according to yeast strain nomenclature, it is better to use "upf1Δ" instead of "Δupf1".

Line 343: I suggest adding a short comment here on using the hoΔ strain as a control (it is actually present in Methods section, but as this issue is mentioned in Fig. 3, it is better to shortly explain this here as well).

Line 392, "the translational competition… between… AUGs": I suggest re-phrasing.

Line 404, "The PIC occupies 19 nt": the authors should be careful in stating this, as it is well known that a portion of mRNA covered by the 40S ribosomal subunit is actually ~28-30 nts (shown by RNase digestion, toe-printing, as well as in structural studies). In some cases, however, a 3'-proximal portion of mRNA is displaced from the mRNA entry channel, thus reducing the footprint length - but this is likely an artificial situation (a result of complex fixation or smth. like that). This does not affect the analysis (as the P-site is still at the 13-15th position), but just textually, it is better to avoid such statement.

Line 409 and Fig. 5A, B: the words about "hitting the 5' cap" sound not very serious. The m7G cap itself can be easily accommodated within the 40S exit channel (it follows from the fact, for example, that mRNAs with no or extremely short leaders can initiate translation quite efficiency in eukaryotic systems, see PMID 27892500, 27401559, 34782646 and some others). However, it is logically to assume that in most cases (normally) the cap is bound by eIF4E, which cannot accommodate within the mRNA channel. Thus, I suggest a correction of "hits the 5'-cap" to "hits the eIF4E-cap" or smth. similar.

Lines 466-468: it is actually unclear, why nucleotide usage frequency does matter and why the "numbers were qualitatively consistent" (as far as I understood, the latter means that 0.02 is "qualitatively consistent" with ~0.16 - which is indeed literally true but nevertheless makes little sense). Please either explain or remove this phrase.

Lines 577-578: To my opinion, a successful AUG recognition in the case of leaderless mRNAs (or mRNA with extremely short 5' UTRs, see references above) is worthy of more detailed consideration here, as they provide a really good argument for the findings described. In 2016, T.Pestova (PMID 27401559) used this fact to confirm a model of mRNA "threading" into the mRNA-binding channel. However, it is hard to imagine that the mRNA 5'-end is indeed threaded through both entry and exit channels. The current study, together with those mentioned above, provide arguments for a more plausible model where the mRNA is "slotted" into the channels, followed by some back scanning.

Line 589, "an opportunity to computational model the dynamics": please re-phrase.

Lines 606-608: Is it really necessary to confirm this self-evident fact by these citations? There are actually multiple examples of efficiently translated mRNAs with uORFs (in human, for example, almost a half of mRNAs have at least one uORF in their 5' UTR, yet many of them are efficiently translated; in yeast uORFs have much more deleterious effect on translation, but nevertheless such cases are also not exceptionally rare, to be confirmed by citing particular cases).

Lines 624-628: Here citing is not completely correct. In Vassilenko et al., the authors used two in vitro translation systems, the mammalian Krebs-2 cell extract (working at 30 C) and WGE (25 C). The scanning rates were 6 and 8 nt/s, respectively. The elongation rates, measured in the same systems under the same conditions, were 3 and 3-4.5 nt/s (depending on the K+ concentration), respectively - i.e. twice as slow. In contrast, Ingolia et al. measured elongation rate in living cells and (not surprisingly) obtained much higher rates. By the way, recently the same approach was applied to organs of living mice (PMID 33264395) and produced similar (although not identical) values: 13-20 nt/s, depending on organ - but, again, they should not be compared to scanning rates obtained in vitro (even more so at different temperatures, as mammalian cells are cultured at 37 C).

Lines 638-639: I'm not an expert in transcription and replication, but I would take these estimations with caution: for example, for prokaryotes it is well-known that the DNA replication rate is almost an order of magnitude faster than that of transcription (which leads to a well-recognized DNAP/RNAP collisions and appearance of evolutionary conserved machinery to resolve these collisions). Speaking truly, I do not understand what these values can add to the discussion on difference between initiation and elongation rates (as in eukaryotes replication, transcription and translation are separated by nuclear envelope).

Lines 686-689: I would correct the phrase, as from the current text a reader could suggest that the authors of the cited papers were apologists of "diffusion" model (which is not true, as has been many times mentioned in the text). In contrast, in some other papers (e.g. see PMID 24657959 and references therein; see also (Skabkin et al, 2013) cited in the manuscript) it was indeed shown that under some (artificial) conditions the "diffusional wandering" can take place.

Lines 720-725: To me, the evidence in obscure here. But the authors could be interested in the following papers: PMID 26113725, 11333019, and 24657959 (not for citing them here, but just for future).

Line 705: it would also be appropriate to mention (PMID 31510048) here, as in this paper the authors directly observed multiple 40S subunits on a long 5' UTR. There was also evidence from old eIF crosslinking studies, confirming the "untethered" model (from N.Sonenberg lab, although I'm not ready to provide the references now), while conclusions made by (Bohlen et al., 2020) was actually based on a controversial interpretation of their results obtained with a few constructs with different translation efficiency. This is, of course, just my personal opinion and not a recommendation for adding this to the paper text.

Fig. 2C: It is better to adjust Oy axis so that it starts from zero.

Fig. 6E: Please decapitalize "Subtilis"

Fig. 7B: It is probably better to align headers ("NNNaATG… ….dATG") to the center of the panel.

Fig. S1D: please check whether it was really 6204 variants containing two or more dATGs (since, as far as I understand this issue, it should be ~1/64 of 21,598).

Finally , I'd like to repeat that, to my opinion, the manuscript can be accepted to Genome Biol after revision.

- Are the methods appropriate to the aims of the study, are they well described, and are necessary controls included? If not, please specify what is required.
YES
- Are the conclusions adequately supported by the data shown? If not, please explain
ALMOST YES (see details above)
- Are sufficient details provided to allow replication and comparison with related analyses that may have been performed? If not, please specify what is required.
YES
- Does the work represent a significant advance over previously published studies?
YES
- Is the paper of broad interest to others in the field, or of outstanding interest to a broad audience of biologists?
YES

It is my usual policy to reveal my identity to the authors: Sergey Dmitriev.

**Reviewer 2**

In this interesting paper, the authors propose that ribosome scanning, while overall in a 5'-3' direction, actually involves significant backtracking in the 3'-5' direction. While scanning has previously been proposed to follow a Brownian ratchet type mechanism, definitive data supporting the model has not been presented.

Using a large-scale reporter assay, the authors insert ATG codons downstream of the annotated ATG start codon (aATG) of GFP and then examine the impact of these inserted codons on GFP expression. Interestingly, out-of-frame ATG codons inserted within the first 17 bases downstream of the aATG reduced GFP expression and this effect was more pronounced when the inserted ATG codon was in good initiation context. The authors hypothesized, in accord with a few previous publications, that the scanning ribosome oscillates as it moves down the mRNA and that ribosomes that leaky scan over the aATG are caught be these downstream ATG codons and thus fail to translate GFP. As translation initiating from these downstream out-of-frame ATG codons will terminate at out-of-frame stop codons, it is anticipated that the mRNA would be subject to nonsense mediated decay (NMD). In accord with this hypothesis, the authors show that the mRNA levels of the reporters with downstream out-of-frame ATG codons are reduced and that this reduction parallels the position dependency for how the inserted start codons reduce GFP levels. Moreover, the mRNA reductions are suppressed in a strain lacking the NMD factor UPF1. As a low rate of leaky scanning of the aATG codon and subsequent initiation at the downstream ATG codon could substantially reduce GFP mRNA levels, it is difficult to assess the real impact of the inserted ATG codons on translation unless the reporter assays are performed in a strain lacking UPF1.

Using bioinformatic analyses the authors report an apparent selection against out-of-frame start codons in the immediate region downstream of aATG start codons in yeast and human, but not bacterial, mRNAs. They also see a correlation between decreased mRNA levels and the insertion of downstream out-of-frame ATG codons in cancer cells. Finally, using a Markov modeling approach, the authors identify proposed rates of ribosome leaky scanning, nmd, and a pawl (to prevent backward motion of the scanning ribosome) that can account for their results, and they propose that ribosomes scan at a rate of ~100nt/sec and through oscillations of 5'-3'and 3'-5' movements the ribosome on average will scan over an AUG codon 10 times before selecting it as a start site. This is a remarkable proposal, and I would argue that substantial additional evidence is needed before such a claim is made.

Overall, this is an ambitious study. While the results appear internally consistent, there is a real concern of confirmation bias in the interpretation of results. Moreover, some of the claims, like scanning over individual nucleotides in the leader 10 times are so outside the norm, that the authors should be held to a very high standard to support that claim. As detailed below, a trimmed down paper focusing on the well-controlled studies in the paper might be an approach.

Specific comments:

1. The authors show a strong correlation between the impact of inserted AUG codons on GFP activity and GFP mRNA levels. While the authors try to exploit this NMD as a proxy to assess translation from downstream AUG codons, one is left wondering why the original screen was not performed in a upf1 mutant strain of yeast. At a minimum, the authors should perform the experiment in Figure 1F in a upf1 deletion strain. Moreover, the data should be normalized to the reporter with no inserted ATG codon. We need to see how significant the inserted ATG codons lower GFP expression in the absence of NMD to get a real appreciation of the rate of leaky scanning and start codon competition. As the mRNA curves in Figure 3 look very similar to the GFP curve in Figure 1E, it seems that NMD is the big driver on GFP expression and that the start codon competition might be much smaller than the data at first suggests. If the proposed start site competition cannot be observed in the upf1 mutant strain, then one is left to wonder if the proposed competition is real. Moreover, if the competition is substantially reduced in the absence of UPF1, then the estimate for the extent of back scanning might be lowered as well.

2. In the screen in Figure 1, the inserted ATG codons are generating nonsynonymous substitutions in the GFP reading frame. Can one be sure that the different GFP proteins have the same specific activity? The 2A insertion used in Figure 2C would have been a better design for the screen.

3. Lines 204-205: shouldn't the analysis of context effects at the aATG codon be performed on reporters with no dATGs to avoid issues with NMD.

4. Figure S2C: are 40S footprints before and after aATG codons a good proxy for leaky scanning? We do not know all of the features that contribute to 40S footprints (secondary structure, elongation rate of 80S downstream of aATG, etc). Just because the result correlated with GFP does not mean that the analysis was valid.

5. Lines 230-240: this information should also be defined in the legend to Fig. S1D. This nomenclature is used in the supplemental figure before it is defined in the text.

6. Figure 1F: Are these data normalized to the solo ATG as in Figure 1E? In Fig. 1E, the ATG at +8 appears to be down by around 25%; it is difficult to assess the decrease in Figure 1F. It would be nice to specifically reporter the decrease.

7. In Figure 2C, assess GFP and RFP for reporters lacking aATG or dATG, and then normalize the results to these respective reporters. This would enable a better assessment of leaky scanning. While this mRNA is predicted to not be subject to NMD, it is appropriate to normalize the data to mRNA levels.

8. Line 320. This reporter should be described in a bit more detail so that the reader understands that the out-of-frame stop codons were removed from the GFP coding region. How many stop codons were mutated?

9. As noted above, the results in Figure 3 correlate with the GFP levels. This leads to the question on whether the phenomenon being measured is simply NMD and leaky scanning, and not start codon competition. For this reason, we need to see GFP measurements in upf1 mutant - at least for Figure 1F.

10. The uAUG data in Figure 4 raises many questions. First, where are the stop codons for ORFs initiated by the uAUG codons. The assay examining HIS3 complementation will trigger phosphorylation of eIF2. It is not currently clear whether eIF2 phosphorylation affects leaky scanning, but it clearly can affect translation reinitiation. Are the effects in Figure 4 suppressed by deletion of UPF1? There are so many possible confounding effects that it makes it difficult to draw conclusions from the data in Figure 4, despite the apparent correlation to the authors' favorite model.

11. It is not clear what the Markov modeling adds to this paper - it is also possible that this analysis would need to be altered based on results of reporter assays in a upf1 mutant strain.

12. Line 479-480: What is driving the paucity of out-of-frame AUG codons: amino acid sequence of the zero frame or the potential deleterious impact of the out-of-frame start codons? It is not clear whether this data is needed in the paper.

13. Figure 6F: how does this data compare to Figure 1F? The ATG @+8 is <50% in HeLa cells, but perhaps >90% in yeast (assuming that the normalizations are similar). It seems that the downstream ATG codons might be substantially more inhibitory in mammalian cells. Is NMD stronger in human cells?

14. Line 526: it is stated that the mRNA comparison is to the median level of the gene in all other samples of the same cancer type. Is this comparison restricted to mRNA levels for the genes that contain no mutations anywhere within the ORF?

15. Figure 7B: the authors are again using NMD as a potential proxy for alternative initiation. Can they actually see evidence for alternative translation initiation? I do not find the data in Figure 7B compelling: dATG gaining mutations have similar effects in poor versus good context, and the distance dependence is not consistent with the other data in the paper. This analysis could be omitted without significantly affecting the paper.

16. Lines 624-639: the authors should see the paper by Wang et al (https://www.biorxiv.org/content/10.1101/2021.12.08.471614v1) that measured a net scanning rate of ~100 nt/sec in single molecule studies. If the authors want to claim 10x coverage during scanning, then they would need to multiply this rate another 10-fold which does not seem realistic.

17. Line 657: I do not understand what the authors are trying to say. What does "knows" mean?

18. Lines 675-684 and Fig. S4B: This is a very challenging idea. The number of data points in Figure 1E makes it very difficult to distinguish between the two models. Perhaps only one or two data points are intermediate and consistent with the Brownian ratchet model versus the stepwise model. I don't think that the data rules out the stepwise model. Thus, the data in this paper do not provide a clear distinction between the steric hindrance and Brownian ratchet models to explain the impact of downstream AUG codons on aAUG translation start site selection.

**Reviewer 3**

Harnessing massive variants of the reporter and the subsequent FACS-Seq, Li et al. showed the dATG may be inhibitory for the translation from upstream aATG, when dATG locates close enough for aATG. The authors concluded that this effect comes from backward scanning (i.e., 3′-to-5′) of PIC. Given an earlier study by Pestova's group (Skabkin et al. Mol Cell 2013) that shows 40S migration along with RNA in both directions and also a recent work by Gu et al. Nat Commun 2021 that suggested the 3′-to-5′ movement of PIC, the repeated back and forth movement of PIC for AUG recognition is a reasonable scenario. However, the data presented in this study should be carefully interpreted as summarized below.

Major points:
1. Although the authors discussed the possibility, all the data could be interpreted by the steric hindrance by 40S or 80S on dATG: given that the RNA region can be masked by 40S or 80S, aATG may not be accessible when 40S or 80S locates on dATG. Authors guessed that this should happen in sharp contrast with the distance from aATG (Fig. S4B), but this is just speculation. Authors should provide evidence that the effect of dATG does NOT come from the steric hindrance effect.

2. Even when the point above was addressed, the experiments in this work did NOT directly indicate the "Brownian-Rachet" movement, although this reviewer agrees that this model is consistent with the authors' observation. All the statements related to this should be toned down or replaced with the phrase "bi-directional movement" or equivalent.


Minor points:
1. For Fig. 1F, the same experiment but without dATG (i.e., a Solo variant) would be helpful for highlighting the inhibitory effects of dATG.

2. Y-axis labeling in Fig. 4B-4D is a bit confusing. For better understanding for readers, a detailed explanation in legend and method (and easy-to-understand labeling for the Y-axis too) should be considered.

3. Regarding Fig. 6B and 6C, this reviewer recommends placing the same data but for Frame 0 and Frame +2 in the Supplementary Figures.

4. For Fig. S1B, it is worth considering showing the logo around aATG for highly translated RNAs (data from ribosome profiling), instead of highly abundant RNAs.

5. Regarding Fig. S1C for TCP-seq data, typically this type of data should contain both 40S footprints and 80S footprints. The detailed data analysis for the dissection of these two footprints should be provided in the method section (probably this could be by the footprint length). Related to this, this reviewer asks to show the 40S footprint accumulation on dATG. This may be helpful to support the authors' conclusion.

6. Regarding Fig. S2A, this data may represent the effect of the Kozak sequence for translation initiation, however, they did not follow that pattern (over-representation of A at all the positions). Discussion regarding this discrepancy may be required.

7. For the reference of the Kozak sequence, this reviewer recommends citing the structural work by Simonetti et al. Cell Rep 2020.

**Authors’ response to reviewers**

**Reviewer #1**

**Comment #1**

The manuscript entitled "Distance-dependent inhibition of translation initiation by downstream out-of-frame AUGs reveals that ribosome scans in a Brownian ratchet process" by Li et al. describes a really nice study aimed at solving an old conundrum in the field of eukaryotic translation initiation.

Since 1970s, when M.Kozak established her scanning model, a number of important questions arose regarding mechanistic aspects of ribosomal scanning. Among them, one of the most intriguing ones is the question of how the 5'-to-3' directionality of scanning is provided and whether it is unidirectional or includes diffusional wandering back and forth along the 5' untranslated region. A few classic (mostly in vitro) studies were devoted to solving this puzzle, leading to a concept of net unidirectional movement instead of "random wandering" (stochastic bi-directional movement). However, there is yet a lack of understanding whether scanning is strictly unidirectional (governed by, say, an RNA helicase constantly fed by ATP) or includes some limited back-and-forth movements (governed by Brownian motion and directed from 5'- to 3' by an occasional pawl placement - in this case, ATP is needed for the pawl placement). This is actually very important issue, as these two modes should lead to different patterns of AUG recognition. I would note, however, that most of recent studies in the field, unfortunately, have been based on the premise that scanning is strictly unidirectional, although no evidence has been provided for this model.

In this regard, the study by Prof. Qian's group is very important, as it clearly shows that the 43S pre-initiation complex uses limited bi-directional oscillations (governed by Brownian motion) instead of the strictly unidirectional movement. To reveal this, the authors used systems biology approaches, including a reporter library screen, a genome-wide mRNA sequence analysis and analysis of gene expression data from The Cancer Genome Atlas. Importantly, they also built a mathematical model, which allowed them, taking into account their experimental data, revealing quantitative parameters of 43S PIC scanning.

For those who are well acquainted with the literature on this issue, this is a long-expected discovery, as some fragmentary indications of this could be found in the literature before (carefully cited by the authors: i.e. Matsuda and Dreher, 2006; Williams and Lamb, 1989; Terenin et al., 2016; Gu et al., 2021). However, this has never been clearly stated and confirmed by elegant experiments. Thus, to my opinion, this study should be considered as one of the brightest event in the field during the last few years. I would also note an inventive experimental design and the fact that the authors inspected many aspects of their finding and extensively used good positive and negative controls in their experiments, as well as a careful presentation and very competent discussion of the findings. That's why I enthusiastically recommend this manuscript to be published in Genome Biology, once the authors address a few major and several important minor issues listed below.

**Response #1**

We thank Reviewer #1 for the positive evaluation of our work.

**Comment #2**

Major points

1. Lines 269-277 and Fig. 1F: This experiment clearly lacks the analysis of a control construct having no dAUG. It is strange that the authors did not add these data (all the more so that the corresponding construct was indeed present in a similar experiment in HeLa cells).

**Response #2**

We thank reviewer #1 for pointing out this unintentional oversight. The results of this control construct (lacking an additional dAUG inserted) have been added to Fig. 1F of the revised manuscript. The updated figure is shown below.

Note that the data for the other constructs were also updated since we measured them all together in a new experiment.

**Comment #3**

2. Please double check the data, their description, and the protocol details for the experiment described in lines 174-183 (Fig.S1B). I'm really quite amazed that the authors were so lucky to obtain almost ideal correspondence between a GFP/dTomato ratio for a number of variants in the library (where the GFP/dTomato ratio is actually an abstract mathematical estimation, i.e. the result of a complex calculation based on distribution of a particular variant between eight bins (the variant frequencies) obtained after quantification of Illumina reads) and a real GFP/dTomato ratio obtained for the same variants individually (in this case directly measured by flow cytometry). It could be that in the second case the GFP/dTomato ratio was also a result of some calculation based on distribution between some bins (but which frequencies? - there was no Illumina sequencing here). Please clarify this issue.

**Response #3**

We thank Reviewer #1 for raising this concern, which prompted us to scrutinize the accuracy of our estimation of the GFP/dTomato ratio obtained through the FACS-seq experiment (FACS sorting + Illumina sequencing for each bin). We first pulled out the raw data to confirm the strong correlation observed between the GFP/dTomato ratio estimated by FACS-seq and that determined directly by flow cytometry measurements of individual clones (Reviewer #1 was correct that the latter data was not estimated from sorting and Illumina sequencing). These data are shown below.

After finding that these results indeed showed a high degree of correlation, we then performed computational simulations to better understand why these two approaches could result in such highly correlated log_2_(GFP/dTomato) estimations (*r* = 0.99, as shown in Fig. S1B). To this end, we computationally generated data for 20 pseudo variants (each with 1000 cells) with log_2_(GFP/dTomato) of individual cells of each pseudo variant following a normal distribution (based on the mean log_2_(GFP/dTomato) of 20 randomly chosen variants from the GFP dAUG variant library, with a standard deviation equal to 0.5, which is approximately what we observed in the empirical data). The density curves showing the cell distributions of individual pseudo variants are presented below.

We computationally sorted all 20,000 cells from these 20 pseudo variants into eight bins (with an equal width) and estimated the GFP/dTomato ratio for each pseudo variant based on their respective proportions of cells sorted into the eight bins. The bin boundaries are indicated by red vertical lines in the above figure. (The specific parameters for this estimation were as follows: mean log_2_(GFP/dTomato ratio) for the cells sorted into each bin (from bin #8 to bin #1) was -2.63, -1.79, -0.05, 1.00, 2.55, 3.83, 5.22, 6.15, respectively. And the fraction of cells sorted into each bin (from bin #8 to bin #1) was 0.01, 0.04, 0.09, 0.09, 0.16, 0.21, 0.35, 0.05, respectively.)

We estimated the GFP/dTomato ratios using the same formula as for those calculated from FACS-seq data (shown in Fig. S1A), which showed that the *in silico* estimated GFP/dTomato ratios for pseudo variants were highly correlated with the average GFP/dTomato ratios that we initially randomly generated for the 1000 cells (*i.e.*, equivalent to the direct estimation by flow cytometry). See the figure below.

These simulation results thus confirmed that the high correlation in the GFP/dTomato ratio between the FACS-seq approach and direct flow cytometry measurement of individual variants was in fact reasonable and unsurprising, especially when the variation in average GFP/dTomato ratio is sufficiently large among variants (as in our experiments).

We appreciate the opportunity to rigorously re-examine our data and strengthen our conclusions. We have also edited the legend of Fig. S1B to improve clarity in the revised manuscript.

**Comment #4**

3. It would be useful to analyze effects of dAUGs in the upf1Δ strain, to get an estimation of a "clear" dAUG effects on translation initiation without impact of NMD-related mRNA stability. Of course, it is not necessary to repeat en masse analysis in this strain, but it would not be difficult to perform an additional small-scale experiment with a few particular reporter constructs like those shown in Fig. 1F.

**Response #4**

We thank Reviewer #1 for this constructive suggestion. As recommended, we performed both a FACS-seq experiment in the *upf1Δ* background. The results are now provided in Fig. S6B of the revised manuscript (shown below, to be compared with Fig. S2D in the *ho∆* background).

Furthermore, we also performed a small-scale validation experiment for the variants used in Fig. 1F. See the figure below (to be compared with Fig. 1F in the *ho∆* background).

As shown in these figures, the context- and distance-dependent inhibitory effect of out-of-frame dAUGs was still observed in the absence of *UPF1*. These results indicate that while the deletion of *UPF1* can largely restore mRNA levels reduced by proximal out-of-frame dAUGs (shown in Fig. 3 and Fig. S5B–C), rescue at the protein level was not complete, indicating that the inhibitory effects of proximal out-of-frame dAUGs can be observed without any impact of NMD-related variations on mRNA stability.

We would like to thank Reviewer #1 for raising this very important question. Although the additional activation of NMD induced by proximal out-of-frame dAUGs (Fig. 3, on top of the basal NMD activity that can be reflected by the mRNA level of the Solo variants) is a direct consequence of the competition for translation initiation from the dAUG, and can, in turn, reinforce the impacts of translation initiation at out-of-frame dAUGs, we agree with the reviewer that it would be informative to show a “clear” dAUG effects on translation initiation without impact of NMD. With these additional experiments (both FACS-seq and small-scale experiments, added to Fig. S6 of the revised manuscript), in our view the manuscript is substantially improved.

**Comment #5**

Minor points

Line 74-76: [Berthelot et al., 2004] was clearly not the only or the first paper where "the insertion of a strong mRNA secondary structure between the 5′-cap and the aAUG" was shown to "prevent translation initiation" (either in yeast or in other eukaryotes). Some classic review (by M.Kozak or A.Hinnebusch, e.g. PubMedID 16213112, 27313038 or 24499181) could be cited here; if you prefer a research article, it should be probably PMID 3458245, but I suggest using a review instead.

**Response #5**

[Berthelot et al., 2004] has been removed, and two review papers are cited in the revised manuscript.

**Comment #6**

Lines 137-138: Studies that "have observed reduced protein abundance upon the addition of an out-of-frame uATG" are not limited to these three, of course - there have been multiple studies showing that, published since 1970s. Please specify this issue (e.g. systems studies or smth. like that).

**Response #6**

Corrected.

**Comment #7**

Line 156, "resulting in a representative library": the library used by the authors was not representative. They were actually able to use just a few thousands of constructs in their study, while the complete library, to be indeed representative (at least 1 molecule per variant), should include 4e36 molecules, i.e. ~10e21 ones (which is impossible, as such DNA oligos have a weight of ~0.1 kg)! This is not a problem, as they actually had enough dAUG-containing variants to calculate all they needed, but a reader should be informed that the variants taken into analysis were just a little of the huge number of all possible variants.

**Response #7**

We edited the text in the revised manuscript.

**Comment #8**

Lines 206-213: the prevailing importance of "A" in the -3 position, as the only recognizable feature of "good" AUG context in yeast, was described by Hamilton et al., 1987. It should be mentioned here.

**Response #8**

Paper cited in the revised manuscript.

**Comment #9**

Lines 263-264: It is actually a delusion that IRES is a "consensus sequence", so "IRES similarity" makes no sense. All well approved IRESs are long and complex RNA segments, usually having no extensive similarity at the level of nucleotide sequence but instead belonging to a few types at the level of secondary and tertiary structure. You can find the corresponding discussion elsewhere (e.g. PMID 27853833, 23378589, 16314320, 34565312). I recommend removing this analysis form the text (as well as from Fig. S3), so as not to compromise this high-competent study. Please also check whether citing Kudla et al., 2009 (a study of coding-sequence determinants of gene expression in E.coli) is relevant here.

**Response #9**

The results related to “IRES similarity” have been completely removed from the revised manuscript. The 2009 paper by Kudla et al. was also removed from the revised manuscript.

**Comment #10**

Lines 294-305 and Fig. 2A: I suggest using normalization to results obtained with the corresponding particular Solo constructs: Solo(1A/1G/1C/1T). To my opinion, using the normalization to Solo(1N) is misleading, as a reader may not understand why in the two top subpanels addition of dATGs "stimulated" translation (it was actually the good aAUG context that stimulated, but at first sight it is unclear).

**Response #10**

Agreed. Such normalization has been applied to all relevant figures throughout the revised manuscript (Fig. 2A, S2D, S5B-C, S6B, S9A, S9C, S10A and S10C).

**Comment #11**

Line 308: "an overlapping dual-fluorescent reporter": please re-phrase.

**Response #11**

In the revised manuscript, the reporter is rephrased to: A reporter construct carrying two fluorescent proteins, GFP and dTomato, encoded in different reading frames (hereafter referred to as a dual-frame reporter).

**Comment #12**

Line 342 and elsewhere: according to yeast strain nomenclature, it is better to use "upf1Δ" instead of "Δupf1".

**Response #12**

Thanks! Corrected throughout the revised manuscript.

**Comment #13**

Line 343: I suggest adding a short comment here on using the hoΔ strain as a control (it is actually present in Methods section, but as this issue is mentioned in Fig. 3, it is better to shortly explain this here as well).

**Response #13**

We added one sentence here in the revised manuscript to clarify that the *ho∆* strain was used as the control throughout the manuscript.

**Comment #14**

Line 392, "the translational competition… between… AUGs": I suggest re-phrasing.

**Response #14**

Rephrased to “The competition for translation initiation”.

**Comment #15**

Line 404, "The PIC occupies 19 nt": the authors should be careful in stating this, as it is well known that a portion of mRNA covered by the 40S ribosomal subunit is actually ~28-30 nts (shown by RNase digestion, toe-printing, as well as in structural studies). In some cases, however, a 3'-proximal portion of mRNA is displaced from the mRNA entry channel, thus reducing the footprint length - but this is likely an artificial situation (a result of complex fixation or smth. like that). This does not affect the analysis (as the P-site is still at the 13-15th position), but just textually, it is better to avoid such statement.

**Response #15**

We thank Reviewer #1 for this information. It has been corrected in the revised manuscript.

**Comment #16**

Line 409 and Fig. 5A, B: the words about "hitting the 5' cap" sound not very serious. The m7G cap itself can be easily accommodated within the 40S exit channel (it follows from the fact, for example, that mRNAs with no or extremely short leaders can initiate translation quite efficiency in eukaryotic systems, see PMID 27892500, 27401559, 34782646 and some others). However, it is logically to assume that in most cases (normally) the cap is bound by eIF4E, which cannot accommodate within the mRNA channel. Thus, I suggest a correction of "hits the 5'-cap" to "hits the eIF4E-cap" or smth. similar.

**Response #16**

We thank Reviewer #1 for this suggestion. It has been corrected in the revised manuscript.

**Comment #17**

Lines 466-468: it is actually unclear, why nucleotide usage frequency does matter and why the "numbers were qualitatively consistent" (as far as I understood, the latter means that 0.02 is "qualitatively consistent" with ~0.16 - which is indeed literally true but nevertheless makes little sense). Please either explain or remove this phrase.

**Response #17**

This sentence has been removed from the revised manuscript.

**Comment #18**

Lines 577-578: To my opinion, a successful AUG recognition in the case of leaderless mRNAs (or mRNA with extremely short 5' UTRs, see references above) is worthy of more detailed consideration here, as they provide a really good argument for the findings described. In 2016, T.Pestova (PMID 27401559) used this fact to confirm a model of mRNA "threading" into the mRNA-binding channel. However, it is hard to imagine that the mRNA 5'-end is indeed threaded through both entry and exit channels. The current study, together with those mentioned above, provide arguments for a more plausible model where the mRNA is "slotted" into the channels, followed by some back scanning.

**Response #18**

We thank Reviewer #1 for this constructive suggestion. In the revised manuscript, we added a paragraph discussing the backward scanning explanation for the absence of “blind spot” under the slotting model (see below).

*It remains controversial as to how eIF4E-bound mRNA initially enters the mRNA-binding channel of the small ribosomal subunit. A “slotting” model proposes that eIF4E-bound mRNAs “slot” directly into the mRNA-binding channel whereas a “threading” model hypothesizes that mRNAs “thread” into the mRNA-binding channel after disruption of the eIF4E-cap interaction. The “slotting” model was supported by the exit-tunnel location of eIF4E relative to the translational initiation complex in a structural analysis [69] and by the observation of 5′-UTR length-dependent translation inhibition by tethering eIF4E to the 5′-cap [54]. The “slotting” model would further predict the existence of a translation “blind spot” (i.e., AUG triplets sufficiently close to the 5′-cap cannot be recognized due to the steric hindrance between eIF4E-cap and the small ribosomal subunit) under a strictly unidirectional scanning model [70], and this prediction is contradictory to the observations that eukaryotic mRNAs with extremely short 5′-UTRs could still initiate translation [54, 70, 71]. Nevertheless, this apparent contradiction can be resolved by considering the Brownian ratchet scanning process because AUGs close to the 5′-cap can still be scanned through a 3′–5′ PIC movement after the eIF4E-bound mRNA is “slotted” into the mRNA-binding channel.*

**Comment #19**

Line 589, "an opportunity to computational model the dynamics": please re-phrase.

**Response #19**

Edited.

**Comment #20**

Lines 606-608: Is it really necessary to confirm this self-evident fact by these citations? There are actually multiple examples of efficiently translated mRNAs with uORFs (in human, for example, almost a half of mRNAs have at least one uORF in their 5' UTR, yet many of them are efficiently translated; in yeast uORFs have much more deleterious effect on translation, but nevertheless such cases are also not exceptionally rare, to be confirmed by citing particular cases).

**Response #20**

We agree. Citations removed.

**Comment #21**

Lines 624-628: Here citing is not completely correct. In Vassilenko et al., the authors used two in vitro translation systems, the mammalian Krebs-2 cell extract (working at 30 C) and WGE (25 C). The scanning rates were 6 and 8 nt/s, respectively. The elongation rates, measured in the same systems under the same conditions, were 3 and 3-4.5 nt/s (depending on the K+ concentration), respectively - i.e. twice as slow. In contrast, Ingolia et al. measured elongation rate in living cells and (not surprisingly) obtained much higher rates. By the way, recently the same approach was applied to organs of living mice (PMID 33264395) and produced similar (although not identical) values: 13-20 nt/s, depending on organ - but, again, they should not be compared to scanning rates obtained in vitro (even more so at different temperatures, as mammalian cells are cultured at 37 C).

**Response #21**

We thank Reviewer #1 for this insightful comment. We revised the paragraph in the revised manuscript to avoid unfair comparison between *in vivo* and *in vitro* experimental data (See below).

*Previous studies showed that the PIC scans at a rate of ∼6–8 nt/s in eukaryotic cell lysates based on the relationship between the 5′-UTR length and the time required for the first round of translation products [50] (a similar estimation of ~10 nt/s was obtained independent by another group [26]; see also [55] for a different estimation). However, it is puzzling that this estimated scanning rate is only approximately two times faster than the rate of translation elongation (~3–4.5 nt/s) measured in the same experimental conditions [50]. Note that PIC scanning only inspects for complementarity to the Met-tRNAi anticodon while translation elongation includes several complicated steps such as tRNA selection (which by itself includes multiple rounds of inspection of codon-anticodon complementarity), peptide bond formation, and translocation [56, 57]. We thus propose that PIC scanning rates measured in previous studies represent the “net” scanning rate, and can be better understood in the framework that each triplet is scanned back and forth: the actual scanning rate over individual nucleotides is approximately ten times faster than the net scanning rate, or ~60–100 nt/s.*

**Comment #22**

Lines 638-639: I'm not an expert in transcription and replication, but I would take these estimations with caution: for example, for prokaryotes it is well-known that the DNA replication rate is almost an order of magnitude faster than that of transcription (which leads to a well-recognized DNAP/RNAP collisions and appearance of evolutionary conserved machinery to resolve these collisions). Speaking truly, I do not understand what these values can add to the discussion on difference between initiation and elongation rates (as in eukaryotes replication, transcription and translation are separated by nuclear envelope).

**Response #22**

These values have been removed from the revised manuscript.

**Comment #23**

Lines 686-689: I would correct the phrase, as from the current text a reader could suggest that the authors of the cited papers were apologists of "diffusion" model (which is not true, as has been many times mentioned in the text). In contrast, in some other papers (e.g. see PMID 24657959 and references therein; see also (Skabkin et al, 2013) cited in the manuscript) it was indeed shown that under some (artificial) conditions the "diffusional wandering" can take place.

**Response #23**

We agreed. Citations have been corrected in the revised manuscript.

**Comment #24**

Lines 720-725: To me, the evidence in obscure here. But the authors could be interested in the following papers: PMID 26113725, 11333019, and 24657959 (not for citing them here, but just for future).

**Response #24**

We agree with Reviewer #1 after carefully reading these papers. The inaccurate sentence has been removed from the revised manuscript.

**Comment #25**

Line 705: it would also be appropriate to mention (PMID 31510048) here, as in this paper the authors directly observed multiple 40S subunits on a long 5' UTR. There was also evidence from old eIF crosslinking studies, confirming the "untethered" model (from N.Sonenberg lab, although I'm not ready to provide the references now), while conclusions made by (Bohlen et al., 2020) was actually based on a controversial interpretation of their results obtained with a few constructs with different translation efficiency. This is, of course, just my personal opinion and not a recommendation for adding this to the paper text.

**Response #25**

We thank Reviewer #1 for these valuable comments. We carefully read these references and in the revised manuscript, we discussed our observations based on the “cap-severed” scanning model. The results of PMID 31510048 are also mentioned in the revised manuscript.

**Comment #26**

Fig. 2C: It is better to adjust Oy axis so that it starts from zero.

**Response #26**

Y axis starts from zero in the revised manuscript.

**Comment #27**

Fig. 6E: Please decapitalize "Subtilis"

**Response #27**

Decapitalized.

**Comment #28**

Fig. 7B: It is probably better to align headers ("NNNaATG… ….dATG") to the center of the panel.

**Response #28**

Thanks. The figure is removed from the revised manuscript as suggested by Reviewer #2 (Comment #16).

**Comment #29**

Fig. S1D: please check whether it was really 6204 variants containing two or more dATGs (since, as far as I understand this issue, it should be ~1/64 of 21,598).

**Response #29**

Thanks for your careful reading of our manuscript. We confirmed that 6204 was the correct number of variants. Yes, for each potential triplet position, the probability of ATG occurrence is approximately 1/64 (assuming that A, T, C, and G each have a 25% probability). Therefore, for ~23 potential dATG sites (a second dATG in additions to the fixed one), the expected number of variants containing two or more dATGs should be approximately (1 − (63/64)^23^) × 21,598 = 6563, which is comparable to the number we observed.

**Comment #30**

Finally , I'd like to repeat that, to my opinion, the manuscript can be accepted to Genome Biol after revision.

**Response #30**

We thank Reviewer #1 again for the thoughtful and constructive comments on our manuscript.

- Are the methods appropriate to the aims of the study, are they well described, and are necessary controls included? If not, please specify what is required.

YES

- Are the conclusions adequately supported by the data shown? If not, please explain

ALMOST YES (see details above)

- Are sufficient details provided to allow replication and comparison with related analyses that may have been performed? If not, please specify what is required.

YES

- Does the work represent a significant advance over previously published studies?

YES

- Is the paper of broad interest to others in the field, or of outstanding interest to a broad audience of biologists?

YES

It is my usual policy to reveal my identity to the authors: Sergey Dmitriev.

**Reviewer #2:**

**Comment #1**

In this interesting paper, the authors propose that ribosome scanning, while overall in a 5'-3' direction, actually involves significant backtracking in the 3'-5' direction. While scanning has previously been proposed to follow a Brownian ratchet type mechanism, definitive data supporting the model has not been presented.

Using a large-scale reporter assay, the authors insert ATG codons downstream of the annotated ATG start codon (aATG) of GFP and then examine the impact of these inserted codons on GFP expression. Interestingly, out-of-frame ATG codons inserted within the first 17 bases downstream of the aATG reduced GFP expression and this effect was more pronounced when the inserted ATG codon was in good initiation context. The authors hypothesized, in accord with a few previous publications, that the scanning ribosome oscillates as it moves down the mRNA and that ribosomes that leaky scan over the aATG are caught be these downstream ATG codons and thus fail to translate GFP. As translation initiating from these downstream out-of-frame ATG codons will terminate at out-of-frame stop codons, it is anticipated that the mRNA would be subject to nonsense mediated decay (NMD). In accord with this hypothesis, the authors show that the mRNA levels of the reporters with downstream out-of-frame ATG codons are reduced and that this reduction parallels the position dependency for how the inserted start codons reduce GFP levels. Moreover, the mRNA reductions are suppressed in a strain lacking the NMD factor UPF1. (1) As a low rate of leaky scanning of the aATG codon and subsequent initiation at the downstream ATG codon could substantially reduce GFP mRNA levels, it is difficult to assess the real impact of the inserted ATG codons on translation unless the reporter assays are performed in a strain lacking UPF1.

Using bioinformatic analyses the authors report an apparent selection against out-of-frame start codons in the immediate region downstream of aATG start codons in yeast and human, but not bacterial, mRNAs. They also see a correlation between decreased mRNA levels and the insertion of downstream out-of-frame ATG codons in cancer cells. Finally, using a Markov modeling approach, the authors identify proposed rates of ribosome leaky scanning, nmd, and a pawl (to prevent backward motion of the scanning ribosome) that can account for their results, and they propose that ribosomes scan at a rate of ~100nt/sec and through oscillations of 5'-3'and 3'-5' movements the ribosome on average will scan over an AUG codon 10 times before selecting it as a start site. This is a remarkable proposal, and I would argue that substantial additional evidence is needed before such a claim is made.

Overall, this is an ambitious study. While the results appear internally consistent, there is a real concern of confirmation bias in the interpretation of results. (2) Moreover, some of the claims, like scanning over individual nucleotides in the leader 10 times are so outside the norm, that the authors should be held to a very high standard to support that claim. As detailed below, a trimmed down paper focusing on the well-controlled studies in the paper might be an approach.

**Response #1**

We thank Reviewer #2 for their positive evaluation of the significance of our study and for their constructive comments suggesting possible improvements. Following their insightful recommendations, we have extensively revised the manuscript, by providing more experimental data, adding adequate controls, and removing results that have possible alternative explanations.

(1) We added experimental results (both FACS-seq and small-scale validation experiments) in the background of *upf1∆* to the revised manuscript. See our response #2 for details.

(2) We discussed possible reasons why the net PIC scanning rate estimated in Wang et al preprint (100 nt/second, as mentioned by Reviewer #2 in Comment #17) appears one order of magnitude greater than two previous studies (10 nt/second by Berthelot et al. and 6–8 nt/second by Vassilenko et al.). The PIC scanning rate over individual nucleotides based on these two fully reviewed studies, which would be approximately 60–100 nt/second, appears normal in our view. Please see our response #17 for details.

**Comment #2**

Specific comments:

1.	The authors show a strong correlation between the impact of inserted AUG codons on GFP activity and GFP mRNA levels. (1) While the authors try to exploit this NMD as a proxy to assess translation from downstream AUG codons, one is left wondering why the original screen was not performed in a upf1 mutant strain of yeast. (2) At a minimum, the authors should perform the experiment in Figure 1F in a upf1 deletion strain. (3) Moreover, the data should be normalized to the reporter with no inserted ATG codon. We need to see how significant the inserted ATG codons lower GFP expression in the absence of NMD to get a real appreciation of the rate of leaky scanning and start codon competition. (4) As the mRNA curves in Figure 3 look very similar to the GFP curve in Figure 1E, it seems that NMD is the big driver on GFP expression and that the start codon competition might be much smaller than the data at first suggests. If the proposed start site competition cannot be observed in the upf1 mutant strain, then one is left to wonder if the proposed competition is real. Moreover, if the competition is substantially reduced in the absence of UPF1, then the estimate for the extent of back scanning might be lowered as well.

**Response #2**

We thank Reviewer #2 for these well-considered and highly constructive comments, which prompted us to re-perform the FACS-seq with additional small-scale validation experiments in a *upf1∆* strain, as well as to clarify the relationship between NMD and the competition for translation initiation between closely spaced AUGs.

(1) The apparent inhibitory effect of a proximal out-of-frame dAUG on protein production has two potential sources: (i) the reduction in translation initiation rate at the aAUG because translation initiation at the dAUG consumes PICs and (ii) the elevated activation of NMD induced by translation initiation at the dAUG. Both effects are the consequences of the competition for translation initiation between two closely spaced AUGs, and both contribute to the (total) inhibitory effects of proximal out-of-frame dAUGs on protein production. Therefore, when determining the “complete” inhibitory effects of dAUGs on protein production, in our view, the activation of NMD induced by proximal out-of-frame dAUGs was not an independent factor requiring experimental controls. As an analogy, the NMD activity induced by uAUGs is often not excluded when we describe their inhibitory effects on protein translation, since the NMD activity induced by out-of-frame uAUGs is in fact a direct consequence of uAUG interference in aAUG translation initiation, not an independent factor. The same logic should apply to dAUGs.

We added discussion on the role of NMD activity in the inhibitory effect of dAUGs in the revised manuscript

(2) We agree with Reviewer #2 that performing these experiments in a *upf1∆* strain could further illustrate the contribution of NMD-independent inhibitory effects of dAUGs on protein translation levels. FACS-seq together with small-scale validation experiments in a *upf1∆* strain showed that the inhibitory effects of proximal out-of-frame dAUGs persisted, but were reduced in the absence of *UPF1* (Fig. S6 in the revised manuscript). The figure is also provided below (note that Fig. S6B is to be compared with Fig. S2D, and Fig. S6C is to be compared with Fig. 1F).

(3) Thanks for this suggestion. A variant lacking an inserted ATG was added to Fig. 1F of the revised manuscript (pasted below), and the GFP/dTomato ratios of other variants were normalized to this variant. Such normalization was also performed in other figures of this manuscript where applicable.

(4) We thank Reviewer #2 for their careful reading of our manuscript. The mRNA level curves in Fig. 3B indeed shared a similar trend to the GFP intensity curves in Fig. 1E (*i.e.*, a positive relationship between GFP intensity/mRNA level and the aATG-dATG distance), although there were some quantitative difference. However, the *GFP* mRNA level curve (Fig. 3B) and GFP intensity curve (Fig. 1E and Fig. 2B) may not be directly comparable because these data were obtained through different experimental strategies and equipment. For example, FACS-seq measurement of GFP intensity is based on the measurement of single-cell GFP/dTomato ratios, and dTomato expression was used to normalize GFP intensity in individual cells, which in principle could control for cell-to-cell variation in galactose induction and cell-cycle status. On the contrary, the mRNA levels of GFP variants were measured as a bulk. Therefore, the GFP intensity and mRNA level data may have different linear dynamic range, which makes a quantitative comparison between the two curves not reliable.

Nevertheless, we think Reviewer #2 raised an important question as to whether the inhibitory effect of proximal out-of-frame dATGs can be detected in the absence of the NMD pathway. To address Reviewer #2’s concern on the NMD-independent effect of proximal out-of-frame dAUGs, we performed additional FACS-seq and small-scale experiments in the genetic background of *upf1∆* (Fig. S6 in the revised manuscript, also shown above when answering question (2)). Although the inhibitory effects of proximal out-of-frame dAUGs were reduced upon deletion of *UPF1*, the effects could still be detected in both the FACS-seq and the small-scale validation experiments.

We would like to thank Reviewer #2 again for raising these very important questions, which stimulated considerable and careful thought about the relationship between NMD activity at the mRNA level and the inhibitory effects at the translation level (*i.e.*, consumption of PICs by dAUGs). We have added the new results (both FACS-seq and small-scale validation) to the revised manuscript and added further discussion to clarify the role of NMD. In our view, the manuscript was significantly improved by adding these new data and discussion.

**Comment #3**

2.	In the screen in Figure 1, the inserted ATG codons are generating nonsynonymous substitutions in the GFP reading frame. Can one be sure that the different GFP proteins have the same specific activity? The 2A insertion used in Figure 2C would have been a better design for the screen.

**Response #3**

We thank Reviewer #2 for this valuable suggestion. To determine if the inhibitory effect of proximal dAUGs was caused by the nonsynonymous substitutions in the *GFP* reading frame, we performed the FACS-seq experiments with a 2A self-cleaving sequence inserted between the 30-nt variable region downstream of the aATG and the *GFP* coding sequences. In this experiment, we further controlled for the sequence context of the aAUG and the dAUG. The distance-dependent inhibitory effect of out-of-frame dAUGs persisted. These results were added to the revised manuscript (Fig. S7).

In addition, the variants in Fig. 1F and Fig. S6C in the small-scale validation experiments also shared the identical amino-acid sequence in the N-terminus of the GFP reporter.

**Comment #4**

3.	Lines 204-205: shouldn't the analysis of context effects at the aATG codon be performed on reporters with no dATGs to avoid issues with NMD.

**Response #4**

Following Reviewer #2’s suggestion, we repeated the analysis using the 1805 Solo variants (see Fig. S2A in the revised manuscript) and found that our conclusions remained unchanged.

**Comment #5**

4.	Figure S2C: are 40S footprints before and after aATG codons a good proxy for leaky scanning? We do not know all of the features that contribute to 40S footprints (secondary structure, elongation rate of 80S downstream of aATG, etc). Just because the result correlated with GFP does not mean that the analysis was valid.

**Response #5**

We thank the reviewer for bringing this point to our attention. Careful rereading of the paper in which 40S footprint data were described (Archer et al 2016) led us to agree with Reviewer #2 that the distribution of the 40S footprints may be influenced by the presence of dATGs in the endogenous genes, resulting in pauses of the 40S ribosomes (for the SSU protected fragments with 29 nts or 37 nts in length). Therefore, we removed this analysis from the revised manuscript.

**Comment #6**

5.	Lines 230-240: this information should also be defined in the legend to Fig. S1D. This nomenclature is used in the supplemental figure before it is defined in the text.

**Response #6**

Thanks. We have added this information to the legend of Fig. S1D in the revised manuscript.

**Comment #7**

6.	Figure 1F: Are these data normalized to the solo ATG as in Figure 1E? In Fig. 1E, the ATG at +8 appears to be down by around 25%; it is difficult to assess the decrease in Figure 1F. It would be nice to specifically reporter the decrease.

**Response #7**

We thank Reviewer #2 for this valuable suggestion. We did not generate the Solo variant (lacking additional dATG) for Fig. 1F during the original submission. During revision, we added a variant in Fig. 1F equivalent to the Solo variant in Fig. 1E and normalized all data to this variant. The normalized GFP/dTomato ratio for the variant containing a dAUG at +8 position is 0.41 (Fig. 1F in the revised manuscript). The Duo variants carrying a T at the -3 position of aATG and an A at the -3 position of a [+8] dATG (to control the effects of aAUG and dAUG context between the two experiments) in the FACS-seq experiment had a normalized GFP/dTomato value of 0.50 (with 95% confidence intervals from 0.34–0.66, Fig. S2D in the revised manuscript). This level of reduction was similar, although obtained through different experimental strategies and flow cytometers. We have reported the normalized GFP/dTomato ratios in the legend of Fig. 1F in the revised manuscript.

**Comment #8**

7.	In Figure 2C, assess GFP and RFP for reporters lacking aATG or dATG, and then normalize the results to these respective reporters. This would enable a better assessment of leaky scanning. While this mRNA is predicted to not be subject to NMD, it is appropriate to normalize the data to mRNA levels.

**Response #8**

We thank Reviewer #2 for this suggestion. The respective constructs lacking an aATG or dATG were constructed and their GFP/dTomato values were used to normalize the results. Fig. 2C has been replaced with these new data in the revised manuscript (see below). We also estimated mRNA levels for these constructs, and they did not vary significantly among variants (Fig. S4 of the revised manuscript).

**Comment #9**

8.	Line 320. This reporter should be described in a bit more detail so that the reader understands that the out-of-frame stop codons were removed from the GFP coding region. How many stop codons were mutated?

**Response #9**

Agreed. Six stop codons in +1 frame were mutated. The information is now provided in the Results section and explained in detail in the Methods section. Furthermore, the sequences of mutated “frame +1” stop codons are highlighted in Table S4 of the revised manuscript.

**Comment #10**

9.	(1) As noted above, the results in Figure 3 correlate with the GFP levels. (2) This leads to the question on whether the phenomenon being measured is simply NMD and leaky scanning, and not start codon competition. For this reason, we need to see GFP measurements in upf1 mutant - at least for Figure 1F.

**Response #10**

We thank Reviewer #2 for their careful reading of our manuscript.

(1) Yes, the mRNA levels and GFP levels were highly correlated, which was not unexpected since our data support that NMD is also activated by translation initiation at out-of-frame dAUGs.

(2) Following Reviewer #2’s suggestion, we performed the FACS-seq as well as the small-scale validation experiments in the *upf1∆* genetic background. As shown in Fig. S6 of the revised manuscript, the distance-dependent inhibitory effect of out-of-frame dAUGs could still be observed in the absence of *UPF1,* albeit to a reduced extent, indicating that competition for translation initiation between the two closely spaced AUGs confer additional effects on protein translation.

For more detailed thoughts about the relationship between NMD and the inhibitory effects of proximal out-of-frame dAUGs at the protein translation level, please read our response #2.

**Comment #11**

10.	The uAUG data in Figure 4 raises many questions. First, where are the stop codons for ORFs initiated by the uAUG codons. The assay examining HIS3 complementation will trigger phosphorylation of eIF2. It is not currently clear whether eIF2 phosphorylation affects leaky scanning, but it clearly can affect translation reinitiation. Are the effects in Figure 4 suppressed by deletion of UPF1? There are so many possible confounding effects that it makes it difficult to draw conclusions from the data in Figure 4, despite the apparent correlation to the authors' favorite model.

**Response #11**

We thank Reviewer #2 for this thoughtful comment. To address these concerns, we synthesized a uAUG library and used FACS-seq to estimate the distance dependence of the inhibitory effects of uAUGs (*i.e*., similar to the experimental design for dAUGs in Fig. 1). Fig. 4 has been replaced with these new data in the revised manuscript, and Figs. S9–10 for the GFP intensity and mRNA level results in the *hoΔ* or *upf1Δ* backgrounds were also added.

Fig. 4 and the corresponding Results section are shown below:

***Distance-dependent translational inhibition by upstream AUGs (uAUGs)***

*In general, proximity to the 5′-cap grants an AUG triplet some advantages in competition to initiate translation since they are scanned first [6, 15]. Consistent with this hypothesis, it has been widely reported that out-of-frame uAUGs can inhibit translation at the aAUG because the uAUG can retain a proportion of PICs that would otherwise initiate translation at the aAUG [15, 19, 20]. Given our results showing competition for initiation between a closely spaced aAUG-dAUG pair, we further predicted that a closely spaced uAUG-aAUG pair would also compete for translation initiation. That is, when a uAUG is near the aAUG, a PIC that misses the uAUG (due to leaky scanning) may be retained by the nearby aAUG, thereby reducing the likelihood that the PIC will oscillate 3′–5′ and recognize the uAUG. Therefore, the Brownian ratchet scanning model further predicted that the inhibitory effect by an out-of-frame uAUG should diminish with decreasing uAUG-aAUG distance (Fig. 4A).*

*To test if the inhibitory effects of an out-of-frame uAUG indeed depends on its distance to the aAUG, we synthesized a uATG variant library (Fig. 4B) similar to the dATG variant library. Specifically, we introduced uATGs by chemically synthesizing a 30-nt DNA oligo with doped nucleotides (N) upstream of a fixed aATG triplet (Fig. 4B). To increase the proportion of variants carrying a uATG, we synthesized 28 additional DNA oligos, each with a uATG fixed at one of the 28 possible upstream triplet positions (Fig. 4B). We fused these DNA oligos with the full-length GFP sequence and integrated the fusion constructs individually into the yeast genome. GFP intensity and mRNA level of individual variants were then measured by FACS-seq and en masse RNA sequencing of the variable region, respectively, following the same protocol as used for the dATG library. GFP intensity and mRNA level were quantified in two biological replicates, and since the values were highly correlated between replicates (Additional file 1: Fig. S8), the data from both replicates were pooled in subsequent analyses.*

*The 3112 variants containing stop codons in the frame of uATGs and at a position upstream of the aATG were excluded from the subsequent analyses to avoid their potential impacts of translation reinitiation (i.e., the ability of some short upstream open reading frames to retain the 40S subunit on mRNA post-termination, then reinitiate translation at a downstream AUG). We confirmed that the 15,586 Duo variants containing in-frame uATGs indeed showed higher GFP intensities than the 2872 Solo variants and the Duo variants containing out-of-frame uATGs indeed had lower GFP intensities (Fig. 4B, Additional file 1: Fig. S9). These results led us to further examine the impacts of uATG position relative to aATG, as well as uATG sequence context, on GFP intensities among the Duo variants.*

*To this end, Duo variants were grouped according to the position of their inserted uATGs, in a manner similar to that used for grouping dATGs in Fig. 1E. The results showed that GFP intensities increased with decreasing uATG-aATG distance, a trend which was especially apparent when the distance between the two ATGs was relatively small (Fig. 4B). We also observed that the inhibitory effect of a proximal, out-of-frame uATG was reduced in the variants harboring the aATG in a strong context (Additional file 1: Fig. S9A) or with a uATG in a weak context (Additional file 1: Fig. S9B). We then performed an additional, small-scale experiment in which the flanking sequence was strictly controlled in order to further characterize the distance-dependent inhibitory effects of uAUGs. Specifically, we introduced an out-of-frame uATG in a weak context (with a T in the −3 position) at positions −25, −19, −13, or −7 upstream of the aATG in a strong context (with an A in the −3 position, Fig. 4C). We observed that decreasing distance between the two ATGs indeed resulted in a gradual increase in GFP intensity (Fig. 4C). Taken together, these results showing distance- and context-dependent inhibitory effects by out-of-frame uATGs suggested that aAUGs compete with proximal uAUGs to initiate translation.*

*Translation initiation at out-of-frame uAUGs would result in activation of the NMD pathway. Therefore, if the reduced inhibitory effect of proximal out-of-frame uAUGs did result from competition for translation initiation between the aAUG and a proximal uAUG, we predicted that GFP mRNA level should increase with decreased uAUG-aAUG distance. En masse quantification of mRNA levels for the out-of-frame uATG variants in the hoΔ background revealed that GFP mRNA level was higher in the variants with smaller uAUG-dAUG distance, weaker uAUG context, and/or stronger aAUG context (Additional file 1: Fig. S9C, D), and upon UPF1 deletion, the GFP transcripts of Duo variants carrying an out-of-frame uAUG were restored to levels comparable with that of Solo variants (Additional file 1: Fig. S10A, B), regardless of the uAUG-aAUG distance and the sequence context. Similar to the observation of the dATG variants, NMD inactivation also did not result in a full restoration of GFP intensity in these out-of-frame uATG variants (Additional file 1: Fig. S10C, D). These results thus indicated that the distance- and context-dependent inhibitory effect of out-of-frame uAUGs was indeed a consequence of competition for translation initiation between a pair of uAUG and aAUG.*

**Comment #12**

11.	(1) It is not clear what the Markov modeling adds to this paper - (2) it is also possible that this analysis would need to be altered based on results of reporter assays in a upf1 mutant strain.

**Response #12**

(1) We observed in our study that uAUG placed at −7 position only reduced GFP intensity by ~50% (Fig. 4B, both uAUG and aAUG are in the N-context), clearly violating the first-AUG rule when two AUGs are sufficiently close to each other. Similar phenomena were also observed based on a few variants reported in previous studies (Matsuda and Dreher, 2006; Gu et al., 2021). These phenomena cannot be well explained by occasional 3′–5′ backward scanning but is expected when each triplet is scanned back and forth for many times so that the first AUG only has a trivial advantage for translation initiation by being firstly scanned by the PIC.

However, the frequency of the backward PIC movement (in the 3′–5′ direction) remains not quantified. Our study generated massive dATG variants, providing us a unique opportunity to computationally model the dynamics of PIC movement. Our simulation indicates that the GFP intensities of the 15,242 dATG variants are consistent with a Brownian ratchet model; and if the Brownian ratchet model is correct, then the parameter space could be predicted. We therefore used MCMC algorithm to establish the possible parameters of dynamic of scanning. Our computational modeling revealed that the backward PIC movement is not some occasional relaxation as described in previous reviews (Hinnebusch, 2014; Jackson et al., 2010), but is widespread that each triplet on average is scanned back and forth by the PIC for a dozen of times or so.

Furthermore, with the mass dATG variant data, we can quantitatively distinguish the ATP-independent diffusion model with the Brownian ratchet model. Both models rely on the 5′–3′ and 3′–5′ PIC movement but have different relative frequencies of 5′–3′ vs. 3′–5′ movement. These models are undistinguishable qualitatively with a few dATG variants. We tested these two models using our massive GFP intensity data and provided support for the Brownian ratchet scanning model. This point has been clarified in the revised manuscript. Therefore, we respectfully request that we retain the MCMC analysis in this study.

(2) We think that reviewer #2 has raised a great point as to whether parameters estimated by MCMC algorithms from one dataset can be applied to another. To test the robustness of parameter estimation, we predicted GFP intensities for the dATG variants in the *upf1∆* background, using the parameters estimated from the variants in the *ho∆* background, except that the probability of NMD activation upon translation initiation at an out-of-frame AUG, *p.NMD*, was set to zero. Note that only two variants were shared in the two libraries (transformation of the mixed oligo was performed independently in two different genetic backgrounds), so they are effectively independent datasets. The GFP intensity of the dATG variants in the *upf1∆* background (Fig. S6B, new FACS-seq results added to the revised manuscript) were largely recapitulated, supporting the effectiveness of MCMC modeling. We agree with Reviewer #2 that it would be important to cross-validate the results of MCMC modeling. Therefore, we performed two additional analyses regarding the AUG context-specificity and uATG variant libraries to confirm the MCMC-based parameter estimation. All these results were added to the revised manuscript.

Fig. S11E–H and the corresponding discussion in the revised manuscript are shown below.

*Using an MCMC algorithm, we quantitatively investigate the Brownian ratchet scanning process by estimating the parameters that best fit the observed GFP intensity data in our dATG variant library. Note that although not highlighted in the Results, we also performed additional analyses to confirm the accuracy of parameter estimation. For example, we simulated the Brownian ratchet scanning process using the context-dependent p.Leakage (estimated from the Solo variants, Fig. 5G) and confirmed that the GFP intensities could be successfully recapitulated for a specific aATG/dATG context combination (Additional file 1: Fig. S11E). We also predicted GFP intensities without NMD activity (by setting p.NMD to zero) and confirmed that the GFP intensities in the upf1Δ background were largely recapitulated (Additional file 1: Fig. S11F). Moreover, we repeated the MCMC algorithms using the GFP intensity data of the uATG library (Additional file 1: Fig. S11G), and estimated the number of scans for each triplet to be 19.8, with 95% confidence intervals from 9–30 times (Additional file 1: Fig. S11H). This range overlapped with the 95% confidence intervals estimated from the dATG library (6–14 times). Cross-validation using various datasets confirmed the robustness of our computational modeling of the PIC scanning process.*

**Comment #13**

12.	Line 479-480: What is driving the paucity of out-of-frame AUG codons: amino acid sequence of the zero frame or the potential deleterious impact of the out-of-frame start codons? It is not clear whether this data is needed in the paper.

**Response #13**

There are two reasons underlying our hypothesis that the deleterious impacts of out-of-frame dAUGs are the main driving force leading to the paucity of out-of-frame dAUGs. First, the extent of out-of-frame dAUG paucity was affected by dAUG sequence context (nucleotide at its −3 position), suggesting that this paucity of out-of-frame dAUGs is likely related to translation. Second, this phenomenon was not observed in prokaryotes (*E. coli* and *B. subtilis*), in whom AUGs are not recognized by 5′–3′ scanning.

We thank Reviewer #2 for raising this concern, which prompted us to search for additional evidence supporting our hypothesis. We noticed that proximal out-of-frame dATGs should cause stronger detrimental impacts in more highly expressed genes, because the amount of dAUG-initiation induced truncated peptides and the elevated NMD activity both scale with the gene expression level. Therefore, if the detrimental impacts of proximal out-of-frame dATGs is the underlying mechanism of the paucity of proximal out-of-frame dATGs, we further predict that proximal out-of-frame dATGs should be rarer in more highly expressed genes. To test this hypothesis, we compared dATG frequency between highly (or broadly) expressed genes and lowly (or narrowly) expressed genes. The more highly (or broadly) expressed genes exhibited stronger depletion of proximal out-of-frame dATGs, supporting that the deleterious impact of proximal out-of-frame dATGs is likely the driving force for paucity of such dATGs in the genome. These data have been added to the revised manuscript (Fig. S14).

Overall, the section “Depletion of proximal out-of-frame dATGs in yeast and human genomes” was intended to highlight the potential implications of our theoretical study. Specifically, we presented the evolutionary impacts of distance-dependent inhibitory effects of out-of-frame dAUGs on the DNA sequence downstream of the aATG. We have clarified this point in the revised manuscript.

**Comment #14**

13.	Figure 6F: how does this data compare to Figure 1F? The ATG @+8 is <50% in HeLa cells, but perhaps >90% in yeast (assuming that the normalizations are similar). It seems that the downstream ATG codons might be substantially more inhibitory in mammalian cells. Is NMD stronger in human cells?

**Response #14**

We regret that these data were unintentionally presented without normalization, and thus cannot be directly compared. After normalizing the data to variants lacking an inserted dATG, the inhibitory effects of dATGs at the +8 position were similar between humans (0.46, Fig. 6F) and yeast (0.41, Fig. 1F). We have reported these numbers in the legends of Fig. 1F and 6F in the revised manuscript.

Fig. 1F:

Fig. 6F:

**Comment #15**

14.	Line 526: it is stated that the mRNA comparison is to the median level of the gene in all other samples of the same cancer type. Is this comparison restricted to mRNA levels for the genes that contain no mutations anywhere within the ORF?

**Response #15**

We repeated this analysis with genes containing no mutations anywhere within the ORF (pasted below). However, following the suggestions of Reviewer #2, we have removed this section from the revised manuscript (see our Response #16 below).

**Comment #16**

15.	Figure 7B: the authors are again using NMD as a potential proxy for alternative initiation. Can they actually see evidence for alternative translation initiation? I do not find the data in Figure 7B compelling: dATG gaining mutations have similar effects in poor versus good context, and the distance dependence is not consistent with the other data in the paper. This analysis could be omitted without significantly affecting the paper.

**Response #16**

We presented Fig. 7 with the intention to highlight the potential clinical implications of the inhibitory effects of introduced dAUGs. However, we also agree with Reviewer #2 that a trimmed down paper focusing on the well-controlled studies might be a better strategy for data presentation (stated in summary paragraphs). We are therefore following Reviewer #2’s recommendation to remove Fig. 7 and the accompanying results section from the revised manuscript.

**Comment #17**

16.	Lines 624-639: the authors should see the paper by Wang et al (https://www.biorxiv.org/content/10.1101/2021.12.08.471614v1) that measured a net scanning rate of ~100 nt/sec in single molecule studies. If the authors want to claim 10x coverage during scanning, then they would need to multiply this rate another 10-fold which does not seem realistic.

**Response #17**

We thank Reviewer #2 for pointing out this preprint, which uses an elegant experimental design to measure the duration of each step during translation initiation. Note that the estimated net scanning rate (100 nucleotides per second) in this preprint was an order of magnitude faster than that reported in the two previous studies (6–10 nucleotides per second) cited in our manuscript (Berthelot et al., 2004; Vassilenko et al., 2011).

We found that we were unable to understand the data presented in Fig. 2C and Fig. S4A of the Wang et al. preprint. For example, the burst of Cy3.5-uS19 fluorescence (indicative for the recruitment of 43S complex by mRNAs and considered as the beginning of scanning) occurred at the same time the Cy3-Cy5 FRET started (indicative for the 43S complex arriving at the start codon) in our manual inspection. Please see Fig. S4A from Wang et al shown below (with our interpretation of the data shown at the bottom). We were also confused how the time span of the scanning stage was defined because it seemed to us quite arbitrary, albeit this definition is the key to determining the scanning duration. For example, the start of stage (ii) was labeled by Wang et al at the time point when Cy3.5-uS19 fluorescence rose by half, but the end of stage (ii) was labeled at the time point when Cy3-mRNA to Cy5-uS5 FRET had reached maximum efficiency. (Citing from the materials and methods provided in the supplementary information of Wang et al: “The individual association and dissociation events were manually assigned according to the appearance and disappearance of the corresponding fluorescence signals, to obtain the duration of each molecular event.”) Furthermore, we could not understand why uL18-Cy5.5 fluorescence increased during scanning stage and why Cy3.5-uS19 fluorescence decreased rapidly (within 0.5 seconds) during the scanning stage as labeled by Wang et al. We suspected that these inconsistencies stemmed from some unknown technical errors/incorrect understanding of the data, which may also affect the interpretation of fluorescence changes that used to define stage (ii).

Collectively, we respectfully prefer to use the net scanning rate presented in two previous, fully peer-reviewed studies as the foundation for our current estimates of scanning rate. Nonetheless, we cited the Wang et al. paper in the revised manuscript to leave readers to judge for themselves.

The revised text in the Discussion section is shown below.

*Previous studies showed that the PIC scans at a rate of ∼6–8 nt/s in eukaryotic cell lysates based on the relationship between the 5′-UTR length and the time required for the first round of translation products [50] (a similar estimation of ~10 nt/s was obtained independent by another group [26]; see also [55] for a different estimation). However, it is puzzling that this estimated scanning rate is only approximately two times faster than the rate of translation elongation (~3–4.5 nt/s) measured in the same experimental conditions [50]. Note that PIC scanning only inspects for complementarity to the Met-tRNAi anticodon while translation elongation includes several complicated steps such as tRNA selection (which by itself includes multiple rounds of inspection of codon-anticodon complementarity), peptide bond formation, and translocation [56, 57]. We thus propose that PIC scanning rates measured in previous studies represent the “net” scanning rate, and can be better understood in the framework that each triplet is scanned back and forth: the actual scanning rate over individual nucleotides is approximately ten times faster than the net scanning rate, or ~60–100 nt/s.*

We also provided qualitative (and somewhat intuitive) explanations for the parameters estimated from the MCMC approach, based on the experimental data from this and previous studies in Discussion (See below).

*Our computational modeling of the massive GFP intensity data revealed a 77% average leakage rate for each single scan of an AUG triplet by the PIC and that the net leakage rate of 8% was achieved by multiple scans of the same triplet. A net leakage rate of 8% may ostensibly seem high considering the volume and variety of functional proteins that require accurate translation to maintain cellular function. However, we propose that this leakage rate is reasonable for two reasons. First, the addition of a second in-frame initiation codon as an auxiliary initiation site for translation initiation significantly elevates GFP levels (orange box in Fig. 1D), indicating that a substantial fraction of PICs appears to miss the aAUG and are instead captured by an in-frame, downstream AUG [32]. Second, the canonical ORFs are sometimes efficiently translated in genes bearing out-of-frame uATGs, which indicates that a significant proportion of uAUGs are not recognized by PICs.*

*Our calculation of a 77% average leakage rate for each single scan of an AUG triplet is indeed unexpectedly high. However, this high rate is intuitively consistent with data showing that a dAUG placed at +6 position (which has a G at its −3 position) can reduce GFP intensity by ~50% (i.e., ~50% PICs that could have initiated at the aAUG were retained by a dAUG 5-nt apart, Fig. 1E). Furthermore, when the aATG is in a weak context (with a T at its −3 position), the addition of a dATG at the +6 position even causes a ~75% reduction in GFP intensity (Fig. 2A). Consistently, it was also reported that the proximal, downstream, overlapping ORF in a bicistronic viral mRNA could cause a ~60% reduction in translation initiation at the aATG [29]. Similarly, Gu et al. reported that 50% of PICs were relocated to dAUG placed at +8 position (which has an A at its −3 position), as detected by toe-printing assays [54]. All these observations suggested a substantial single-scan leakage rate.*

*Computational modeling of the massive GFP intensity data suggested that the PIC movement in the 3′–5′ direction is unlikely some occasional relaxation as described in previous reviews [6, 12], but it is widespread that each triplet on average is scanned back and forth by the PIC for a dozen of times or so. While this number of scans may ostensibly seem high, we propose that this number is consistent with our experimental observations. Specifically, we showed that uAUG placed at the −7 position only reduced GFP intensity by ~50% (Fig. 4B, here both uAUG and aAUG are in the N-context), clearly violating the first-AUG rule when two AUGs are sufficiently closely spaced. Similarly, dAUG placed at +8 position also reduced GFP intensity by ~25% (Fig. 1E). These phenomena cannot be well explained by occasional 3′–5′ PIC scanning but is expected when each triplet is scanned back and forth for many times so that the first AUG only has a trivial advantage for translation initiation by being firstly scanned by the PIC.*

**Comment #18**

17.	Line 657: I do not understand what the authors are trying to say. What does "knows" mean?

**Response #18**

Edited.

**Comment #19**

18.	Lines 675-684 and Fig. S4B: This is a very challenging idea. The number of data points in Figure 1E makes it very difficult to distinguish between the two models. Perhaps only one or two data points are intermediate and consistent with the Brownian ratchet model versus the stepwise model. I don't think that the data rules out the stepwise model. Thus, the data in this paper do not provide a clear distinction between the steric hindrance and Brownian ratchet models to explain the impact of downstream AUG codons on aAUG translation start site selection.

**Response #19**

We thank Reviewer #2 for this insightful comment that our GFP intensity data of dATG variants cannot rule out the stepwise model (to reject the steric hindrance hypothesis), which prompted us to carefully consider the predictions of the steric hindrance hypothesis. We agree that the phenomena reported in Figs. 1 & 2 were qualitatively explainable under the strictly unidirectional model as long as the 40S or 80S ribosomes occupying at proximal dAUGs could reduce accessibility to the aAUG (*i.e.*, the steric hindrance effects). In this explanation (*i.e.*, strictly unidirectional PIC movement + steric hindrance effects), the translation initiation rate at the dAUG is independent of the aAUG-dAUG distance (because sequential 5′–3′ decision-making is assumed). Since the NMD activity reflects translation initiation rate at out-of-frame dAUGs, this explanation further predicts that the mRNA level of Duo variants is not affected by the aAUG-dAUG distance. However, the data in Fig. 3 showed that the mRNA level of *GFP* gradually declined with decreasing aATG-dATG distance, indicating more translation initiation at more proximal dAUGs, an observation contradicting the “steric hindrance” explanation above. We have added discussion about these results in the revised manuscript.

Furthermore, the “steric hindrance” explanation is also inconsistent with the observation related to uAUGs in Fig. 4, which is newly added to the revised manuscript. Specifically, the Brownian ratchet model predicts that a closely spaced uAUG-aAUG pair would also compete for translation initiation, and therefore, the inhibitory effect by an out-of-frame uAUG should diminish with decreasing uAUG-aAUG distance (Fig. 4A). On the contrary, under the strictly unidirectional model (plus possible steric hindrance effects), PIC recognition of the aAUG would be unaffected by 5′-trailing PICs, thereby predicting that the inhibitory effect of uAUGs is independent of the uAUG-aAUG distance. To test these two models, we performed new experiments quantifying the inhibitory effect of uAUGs on GFP reporter intensity (Fig. 4, added to the revised manuscript). These new data showed that GFP intensity increased as uAUG-aAUG distance decreased, an observation that is not well-explained by the model involving the strictly unidirectional PIC movement and possible steric hindrance effects but fits well with the Brownian ratchet model. Also note that this phenomenon persisted in the absence of UPF1 (*i.e*., *upf1∆* cells), indicating that it cannot be explained by variation in NMD activity associated with the efficiency of translation initiation at the uAUG (Fig. S10C–D).

Nevertheless, we agree with Reviewer #2 that the possibility that steric hindrance also contributes to the inhibitory effect of dAUGs cannot be excluded, in addition to the Brownian ratchet model. Therefore, we have opted to use more cautious language throughout the manuscript in our support for the Brownian ratchet model. For example, the title of our manuscript has changed from “Distance-dependent inhibition of translation initiation by downstream out-of-frame AUGs reveals that ribosome scans in a Brownian ratchet process” to “Distance-dependent inhibition of translation initiation by downstream out-of-frame AUGs is consistent with a Brownian ratchet process of ribosome scanning”.

**Reviewer #3:**

**Comment #1**

Harnessing massive variants of the reporter and the subsequent FACS-Seq, Li et al. showed the dATG may be inhibitory for the translation from upstream aATG, when dATG locates close enough for aATG. The authors concluded that this effect comes from backward scanning (i.e., 3′-to-5′) of PIC. Given an earlier study by Pestova's group (Skabkin et al. Mol Cell 2013) that shows 40S migration along with RNA in both directions and also a recent work by Gu et al. Nat Commun 2021 that suggested the 3′-to-5′ movement of PIC, the repeated back and forth movement of PIC for AUG recognition is a reasonable scenario. However, the data presented in this study should be carefully interpreted as summarized below.

**Response #1**

We thank Reviewer #3 for their time and efforts in reviewing our study and for their supportive and constructive comments regarding our model. Following Reviewer #3’s suggestion, we used more cautious language throughout the manuscript during revision. For the alternative interpretation (the steric hindrance effect) raised by Reviewer #3, we performed additional experiments by generating thousands of yeast strains, each with an out-of-frame ATG triplet placed upstream of the annotated ATG codon of a GFP reporter gene. In our view, the paper becomes more rigorous after revision.

**Comment #2**

Major points:

1. Although the authors discussed the possibility, all the data could be interpreted by the steric hindrance by 40S or 80S on dATG: given that the RNA region can be masked by 40S or 80S, aATG may not be accessible when 40S or 80S locates on dATG. Authors guessed that this should happen in sharp contrast with the distance from aATG (Fig. S4B), but this is just speculation. Authors should provide evidence that the effect of dATG does NOT come from the steric hindrance effect.

**Response #2**

We thank Reviewer #3 for this insightful comment, which prompted us to carefully consider the issue of a possible steric hindrance effect. We agree with Reviewer #3 that the phenomena reported in Figs. 1 & 2 were qualitatively explainable under the strictly unidirectional model as long as the 40S or 80S ribosomes occupying at proximal dAUGs could reduce accessibility to the aAUG (*i.e.*, the steric hindrance effects). In this explanation (*i.e.*, strictly unidirectional PIC movement + steric hindrance effects), the translation initiation rate at the dAUG is independent of the aAUG-dAUG distance (because sequential 5′–3′ decision-making by the PIC is assumed). Since the NMD activity reflects translation initiation rate at out-of-frame dAUGs, this explanation further predicts that the mRNA level of Duo variants is not affected by the aAUG-dAUG distance. However, the data in Fig. 3 showed that the mRNA level of *GFP* gradually declined with decreasing aATG-dATG distance, indicating more translation initiation at more proximal dAUGs, an observation contradicting the “steric hindrance” explanation above. We have added discussion about these results in the revised manuscript.

Furthermore, the “steric hindrance” explanation is also inconsistent with our observation related to uAUGs in Fig. 4, which is newly added to the revised manuscript. Specifically, the Brownian ratchet model predicts that a closely spaced uAUG-aAUG pair would also compete for translation initiation, and therefore, the inhibitory effect by an out-of-frame uAUG should diminish with decreasing uAUG-aAUG distance (Fig. 4A). On the contrary, under the strictly unidirectional model (plus possible steric hindrance effects), PIC recognition of the aAUG would be unaffected by the 5′-trailing PICs, thereby predicting that the inhibitory effect of uAUGs is independent of the uAUG-aAUG distance. To test these two models, we performed new experiments quantifying the inhibitory effect of uAUGs on GFP reporter intensity (Fig. 4B–C, added to the revised manuscript). These new data showed that GFP intensity increased as uAUG-aAUG distance decreased, an observation that is not well-explained by the model involving the strictly unidirectional PIC movement and possible steric hindrance effects but fits well with the Brownian ratchet model. Also note that this phenomenon persisted in the absence of *UPF1* (*i.e*., *upf1∆* cells), indicating that it cannot be explained by variation in NMD activity associated with the efficiency of translation initiation at the uAUG (Fig. S10C–D).

Although we do not think the occurrence of steric hindrance effects weakens the likelihood that the Brownian ratchet model best explains our data, we cannot fully exclude the possibility that steric hindrance also involves. Thus, we agree with Reviewer #3 that a cautious tone is appropriate, and we have changed the title from “Distance-dependent inhibition of translation initiation by downstream out-of-frame AUGs reveals that ribosome scans in a Brownian ratchet process” to “Distance-dependent inhibition of translation initiation by downstream out-of-frame AUGs is consistent with a Brownian ratchet process of ribosome scanning”.

**Comment #3**

2. Even when the point above was addressed, the experiments in this work did NOT directly indicate the "Brownian-Rachet" movement, although this reviewer agrees that this model is consistent with the authors' observation. All the statements related to this should be toned down or replaced with the phrase "bi-directional movement" or equivalent.

**Response #3**

We thank Reviewer #3 for this suggestion.

In this study, the massive GFP intensity data obtained in the experiments enabled computational modeling of the dynamics of ribosome scanning. We agree with Reviewer #3 that although our observations are best explained by the Brownian-ratchet scanning process among current models on PIC scanning (including the strictly unidirectional model and the ATP-independent diffusion model), this study does not provide evidence that the data can *only* be explained by the Brownian ratchet model (*i.e.*, additional models may be proposed in the future). Therefore, we have changed the title of our paper following Reviewer #3’s suggestion, as also mentioned in our Response #2 above. We have also toned down other statements strongly asserting the Brownian ratchet model throughout the manuscript.

The reason why we chose not to use the phrase "bi-directional movement", as suggested by Reviewer #3 as an alternative option, is that our observations were inconsistent with the ATP-independent diffusion model, which also belongs to the general category of the bi-directional PIC movement. We clarified this point in the revised manuscript (See the MCMC analyses based on massive dATG variants presented in section “Computational modeling reveals that each successive triplet is on average scanned by the PIC approximately ten times”).

**Comment #4**

Minor points:

1. For Fig. 1F, the same experiment but without dATG (i.e., a Solo variant) would be helpful for highlighting the inhibitory effects of dATG.

**Response #4**

Thanks for the suggestion. Controls without an additional ATG inserted were added to Fig. 1F of the revised manuscript. (See below)

**Comment #5**

2. Y-axis labeling in Fig. 4B-4D is a bit confusing. For better understanding for readers, a detailed explanation in legend and method (and easy-to-understand labeling for the Y-axis too) should be considered.

**Response #5**

We thank Reviewer #3 for this comment. Since we have generated our own experimental results for uAUGs, we have deleted the data re-analysis of uAUGs (Fig. 4B–4D) presented in the original submission. The label of newly added data (Fig. 4B and Figs. S9–10) should be sufficiently clear and easily understood.

**Comment #6**

3. Regarding Fig. 6B and 6C, this reviewer recommends placing the same data but for Frame 0 and Frame +2 in the Supplementary Figures.

**Response #6**

Figures have been added to the revised manuscript (Fig. S13).

**Comment #7**

4. For Fig. S1B, it is worth considering showing the logo around aATG for highly translated RNAs (data from ribosome profiling), instead of highly abundant RNAs.

**Response #7**

The logo around aATG for highly translated RNAs (ribo-seq data from Ingolia et al. 2009 Science) is presented in the revised manuscript (Fig. S2B), which is also shown below.

**Comment #8**

5. (1) Regarding Fig. S1C for TCP-seq data, typically this type of data should contain both 40S footprints and 80S footprints. The detailed data analysis for the dissection of these two footprints should be provided in the method section (probably this could be by the footprint length). (2) Related to this, this reviewer asks to show the 40S footprint accumulation on dATG. This may be helpful to support the authors' conclusion.

**Response #8**

(1) The 40S and 80S footprints were separated experimentally by density gradient centrifugation in the study (Archer et al 2016 Nature) to generate the raw TCP-seq data. We clarified this in the revised manuscript.

(2) Although we did see enrichment with 40S footprints at some dAUGs (see two examples pasted below), we have removed this analysis from the manuscript, as recommended by Reviewer #2 (Comment #5). We thank Reviewer #3 for this constructive suggestion all the same.

**Comment #9**

6. Regarding Fig. S2A, this data may represent the effect of the Kozak sequence for translation initiation, however, they did not follow that pattern (over-representation of A at all the positions). Discussion regarding this discrepancy may be required.

**Response #9**

We thank Reviewer #3 for this insightful comment. In the upstream region of the aAUG, the data shown in Fig. S2A are largely consistent with the sequence logo shown in Fig. S2B: “A” is over-represented from −6 to −1 positions, and the −3 position is the most significant. We speculate that the discrepancy between Fig. S2A and Fig. S2B in the downstream region of the aATG is related to the coding potential of these sequences. That is, downstream adenines may be favored for the AUG recognition by the PIC, as suggested by the GFP reporter data in Fig. S2A but are not necessarily favored during the evolution of endogenous genes due to natural selection for their respective specific protein functions (thereby amino-acid sequences). Note that in our experiments, these downstream adenines are in the designed, variable region upstream of a typical coding sequence of GFP reporter. We briefly discussed this discrepancy in the figure legend of the revised manuscript.

**Comment #10**

7. For the reference of the Kozak sequence, this reviewer recommends citing the structural work by Simonetti et al. Cell Rep 2020.

**Response #10**

Reference added.

**Second round of review**

**Reviewer 1**

The authors have thoroughly addressed all the issues I raised in my first review. Both the study and the text have been improved significantly. I recommend this very interesting, well-qualified, and brave study to be accepted for publication in Genome Biol. S.D.

**Reviewer 2**

In this resubmission the authors have substantially improved the manuscript by removing extraneous results and focusing on the key finding – evidence consistent with a Brownian ratchet mechanism of ribosomal scanning. While I believe the data, in general, supports the authors conclusions, I still have a few questions and concerns that have not been adequately addressed:

1) In my view, Figure S6B is one of the most important in the paper. This experiment examines translation in the upf1 mutant and avoids the amplifying effects of NMD. I disagree with the authors’ advocacy of using NMD as a proxy for translation (lines 315-316) because it is based on assumptions including that mRNA turnover will respond in a linear fashion with translational activity of a dAUG. Do we really know that this true? If not, then I feel that the authors should de-emphasize their interpretation of NMD results.

2) The data in Figure S6B should be replotted as in Figure 1E and presented in the paper beside Figure 1E. We need to be able to directly analyze the effects of the dAUGs on translation without the amplifying effect of NMD. Is this data presented by the blue dots in Figure S11F? Likewise, the data in Figure S6C should be shown in main Figure 1, beside panel 1F. This will enable readers to directly appreciate the contribution of NMD to the results; it is noteworthy that eliminating NMD has had a dramatic impact on the p-values of the results.

3) It seems from the data in Figure 2A-B that the competitive effects of the closely spaced dAUG is really driven by the -3T aAUG with -3A/G dAUG constructs. If these three constructs are removed from the analysis, it is not clear that the competition would be observed. This is important because the current interpretations of the composite results might be overstating the leakiness of good context aAUGs and accordingly perhaps overstating the number of times these good contexts are scanned.

4) Line 327: first word “that” should be “when”

5) Line 339: “abolished” is too strong, “reduced” would be more appropriate

6) Line 340: “are consistent with” would be more appropriate than “indicated”

7) The experiment in Figure S7 should have been performed in the upf1 mutant strain.

8) Are the data in Figure 4C from a upf1 mutant or WT strain? To accurately assess translation, it is important to remove the NMD effects from the analysis.

9) The uAUG data is good and nicely supports the authors’ model. It also presents a compelling argument against the occlusion model.

10) I remain concerned about the authors modeling. While I am not an expert on modeling, I wonder (as stated in #3) if the profound effects for the -3T aAUG with -3A/G dAUG constructs are driving the whole competitive phenomenon. If this is true, I don’t know how this would impact the modeling and whether it is appropriate to derive a model driven by only a small subset of the data.

11) Lines 567-570: I think there is a possible flaw in this analysis or I misinterpreted the authors’ argument. Proximal dAUG will trigger NMD and these mRNAs will – due to the NMD – be underrepresented among the highly expressed genes. Thus, we expect to see fewer dAUGs in the most abundant mRNAs. Thus, the lack of dAUGs in highly expressed genes might not be due to selection against high level production of toxic peptides, but simply due to NMD.

12) Lines 583-4: “likely generally drove” seems too strong

13) The data in Figure 6F in not interpretable without RNA levels. As stated above, we know that NMD is amplifying effects with these reporters. As this paper is trying to investigate translation, it is important to remove the effects of mRNA turnover to reveal the true translation effects.

14) Six times in the Discussion and Conclusion, the authors use the word “massive”. I feel that this word is not justified. The authors performed a screen, I would not describe it as massive.

15) Lines 644-646: the insertion of a second inframe AUG increased GFP levels. What was the impact on mRNA levels?

16) Lines 652-663: see point #2 above. In this paragraph the authors interpret a 50% reduction in GFP as indicating a 50% change in utilization of the aAUG versus dAUG. However, the 50% reduction in GFP includes both the translation change and NMD. If the authors examine the data in Fig. S6B, the impacts of the dAUGs are less than the values stated in this paragraph. Again, it is important to focus on translation effects and not combined translation and NMD effects. This same criticism applies to the following paragraph (lines 665-677).

17) Line 802: “promotes” is not the correct word. The presence of an impediment to 5’-3’ motion does not promote 3’-5’ motion, it just blocks forward motion.

18) Finally, I would like to comment on the authors’ rebuttal to reviewer #2, question 17 regarding the recently reported faster scanning rate for eukaryotic translation. From my understanding, it is best to focus on Fig. 2C which clearly shows the time gap between ribosome binding to an mRNA and large subunit association. This time gap, the time of scanning, is quantified in Figure S4C and shows a correlation with the length of the 5’ leader. While it could be argued that the ribosomes in this reconstituted system diffuse at a faster rate down the mRNA due to the lack of some factors that are present in vivo or perhaps do not flip directions as often as in vivo, an alternative possibility is that false assumptions in the authors’ modeling have resulted in overestimating the number of times the ribosome samples each nucleotide as it scans down the mRNA.

Finally, I would point out that the data in the bioRxiv paper reveals that while scanning is fast (100 nt/sec), the post-scanning delay of the ribosome at the start codon before subunit joining is slow (and rate-limiting). Thus, the published 10 nt/sec scanning times, which rely on the appearance of a reporter protein and thus do not directly measure scanning, are dominated by this slow rate of subunit joining and not a slow rate of scanning. This is an important distinction with direct implications on the authors’ proposed rate of scanning (with backtracking).

**Reviewer 3**

The new experiments and analyses and careful wording to describe their findings addressed the concerns of this reviewer. This reviewer recommends this manuscript for publication.

**Authors’ response to reviewers**

Point-by-Point responses to reviewers’ comments

Reviewer #1:
>The authors have thoroughly addressed all the issues I raised in my first review. Both the study and the text have been improved significantly. I recommend this very interesting, well-qualified, and brave study to be accepted for publication in Genome Biol. No further revision is needed.
>>Response: We thank Reviewer #1 for the recommendation of our work.

Reviewer #2:
>In this resubmission the authors have substantially improved the manuscript by removing extraneous results and focusing on the key finding – evidence consistent with a Brownian ratchet mechanism of ribosomal scanning. While I believe the data, in general, supports the authors conclusions, I still have a few questions and concerns that have not been adequately addressed:
>>Response: We thank Reviewer #2 for careful reading of our revised manuscript and providing an additional set of valuable advice. We have revised our manuscript according to these helpful suggestions. We have also edited the manuscript to further improve the clarity of our data presentation.

>1) In my view, Figure S6B is one of the most important in the paper. This experiment examines translation in the upf1 mutant and avoids the amplifying effects of NMD. I disagree with the authors’ advocacy of using NMD as a proxy for translation (lines 315-316) because it is based on assumptions including that mRNA turnover will respond in a linear fashion with translational activity of a dAUG. Do we really know that this true? If not, then I feel that the authors should de-emphasize their interpretation of NMD results.
>>Response: We agree with Reviewer #2 that while NMD activity should be positively correlated with the abundance of translation initiation from out-of-frame dAUGs, they do not necessarily share a linear relationship. Following this suggestion, we have removed the sentence mentioned by Reviewer #2 in the revised manuscript. NMD activity is only used to test if translation initiation occurs at a proximal out-of-frame dAUG in our study.

>2) The data in Figure S6B should be replotted as in Figure 1E and presented in the paper beside Figure 1E. We need to be able to directly analyze the effects of the dAUGs on translation without the amplifying effect of NMD. Is this data presented by the blue dots in Figure S11F? Likewise, the data in Figure S6C should be shown in main Figure 1, beside panel 1F. This will enable readers to directly appreciate the contribution of NMD to the results; it is noteworthy that eliminating NMD has had a dramatic impact on the p-values of the results.
>>Response: We thank Reviewer #2 for expressing their opinion about the relationship between 1) the inhibitory effects of out-of-frame dAUGs on protein synthesis by competing for translation initiation and 2) the NMD effect. The NMD pathway is triggered during translation when a premature termination codon is recognized by ribosomes, so by definition represents a “downstream” effect of translation. Indeed, NMD activity is elevated upon insertion of proximal out-of-frame dAUGs (Figure 3), and therefore, is an important component of the total inhibitory effects caused by the insertion of these dAUGs. In alignment with this view, it is not unexpected that eliminating NMD activity will have an impact on the p-values of the results, as noted by Reviewer #2.
Let us make an analogy using out-of-frame upstream AUGs (uAUGs). In an influential, highly cited review paper on uAUGs (doi: 10.1371/journal.pgen.1003529), Barbosa et al. stated that “uORF-mediated translational control” could “occur through different mechanisms” and NMD is clearly one of them (Figure 1D). Consistent with this view, the inhibitory effects of uAUGs by competing for translation initiation are seldomly studied in an NMD-deficient genetic background (using a few studies that have been cited in our manuscript as examples: 10.15252/msb.20145136, 10.1101/gr.224964.117, 10.1073/pnas.1222534110).
In this study, we defined the “inhibitory effect” as the fraction of reduction in GFP intensity for Duo variants relative to Solo variants (Lines 239-240 before line numbers were removed), and therefore, the effects rising from NMD activity should be included. We suspect that Reviewer #2’s comment arose from confusion between the inhibitory effect of proximal out-of-frame dAUGs and the estimated number of scans for each nucleotide in the Brownian ratchet scanning process. They are related but different things. The latter is indeed sensitive to the “nonlinear” effect of NMD activity, although it has been carefully modeled in our MCMC simulation (Figure 5A). We have further clarified the consideration of the amplification effect of NMD in the section about computational modeling during revision.
Figure 1 is already quite busy, and the GFP intensity results in the upf1 mutants requested by Reviewer #2 reflect only part of the total inhibitory effects of downstream out-of-frame AUGs. Therefore, we hope that we can keep Figures S6B and S6C in the supplementary figure. The figure requested by Reviewer #2 (which was not presented in Figure S11F) is added to Figure S6 during revision (as Figure S6B). The previous Figure S6B is now Figure S6C after revision, which can be directly compared with Figure S2D, with detailed information on the sequence contexts for initiation codons. The previous Figure S6C is now Figure S6D after revision, which can be compared with Figure 1F. Furthermore, we added the summary graph for the uATG library in the upf1 mutants to Figure 4 (side-by-side with the results in the wild-type cells) during revision, which still has space, although not requested by Reviewer #2.

>3) It seems from the data in Figure 2A-B that the competitive effects of the closely spaced dAUG is really driven by the -3T aAUG with -3A/G dAUG constructs. If these three constructs are removed from the analysis, it is not clear that the competition would be observed. This is important because the current interpretations of the composite results might be overstating the leakiness of good context aAUGs and accordingly perhaps overstating the number of times these good contexts are scanned.
>>Response: If the Brownian ratchet scanning model reflects the real translation initiation process, the experimental detection of the inhibitory effect of proximal out-of-frame dAUG would depend on 1) sufficient leaky scanning at the aAUG and 2) efficient translation initiation at the dAUG. Therefore, the difference in sequence contexts of initiation codons mentioned by Reviewer #2 is actually consistent with the Brownian ratchet scanning model. Furthermore, all data shown in Figure S2D were quantitatively fitted in our MCMC modeling, considering variation in leakage rate among sequence contexts of the initiation codon (explained in detail in the second to last paragraph of section “Computational modeling reveals that each successive triplet is on average scanned by the PIC approximately ten times”). The parameters of leakage rates for AUGs in various sequence contexts are provided in Figure 5G and Figure S11D.
It is worth noting that the scanning process by definition is separated from the AUG recognition process (i.e., the PIC scan the mRNA even if there is not an AUG to be recognized). In this study, aAUGs and dAUGs are only used as reporters and perturbators to understand the process of scanning. As a matter of fact, tests of biological hypotheses often demand relatively strong perturbation/reporter systems due to technical errors in experimental measurement. The scanning model (strictly unidirectional vs. Brownian ratchet) could be compared using C/T-context aAUG and A/G-context dAUG does not mean that the supported scanning model only applies to these constructs. Please allow us again to make an analogy using out-of-frame uAUGs. The current working model of translation initiation (i.e., PIC scanning starts with attachment to the 5′cap of an mRNA, after which the PIC migrates along the mRNA one nucleotide at a time, inspecting for the AUG codon) is supported by the evidence showing that the insertion of a uAUG can reduce translation initiation at the aAUG. The effect can be better detected when uAUGs are in stronger sequence contexts (DOI: 10.1073/pnas.1222534110). But it does not mean that the supported model is valid only for the genes containing uAUGs in a strong context. Instead, it is a general rule that applies to the majority of the eukaryotic genes, including the genes without any uAUGs. We thank reviewer #2 for raising this concern, and have provided additional comparisons for observed and predicted GFP intensities in different aATG/dATG sequence context combinations during revision (Fig. S11E, F).

>4) Line 327: first word “that” should be “when”
>>Response: Edited during revision.

>5) Line 339: “abolished” is too strong, “reduced” would be more appropriate
>>Response: Edited to “nearly abolished” during revision. The word “reduced” was not used because the subject of the clause is “reduction”.

>6) Line 340: “are consistent with” would be more appropriate than “indicated”
>> Response: Edited during revision.

>7) The experiment in Figure S7 should have been performed in the upf1 mutant strain.
>>Response: Doing this experiment again (generating ~30,000 dAUG variants and using FACS-seq to determine their respective GFP intensities) would require approximately two months. As stated in our response to comment (3), the downstream NMD effect is an important component of the inhibitory effect of proximal out-of-frame dAUGs. Therefore, we thought that it would be better to detect the total impact of proximal out-of-frame dAUGs (i.e., in the wild-type background). Note that Figure S7 is only a supplementary figure added during revision to address Reviewer #2’s specific concern about how amino acid sequences at the N-terminus could affect GFP folding and fluorescence. The concern could also be addressed using small-scale validation experiments with all variants having identical N-terminus amino acid (Figure 1F and Figure S6C). Furthermore, we also provided massive FACS-seq and mRNA-seq data showing the distance-dependent impact of both proximal out-of-frame dAUGs (Figures 3C and S6B) and out-of-frame uAUGs (Figure S10) in the upf1 mutant. Considering the strength of these experimental results, we respectfully request that this experiment (i.e., 2A-containing constructs) is not performed in the upf1 mutant strain.

>8) Are the data in Figure 4C from a upf1 mutant or WT strain? To accurately assess translation, it is important to remove the NMD effects from the analysis.
>>Response: We have now provided the small-scale uATG experiment in the upf1 mutant in Figure S10E.

>9) The uAUG data is good and nicely supports the authors’ model. It also presents a compelling argument against the occlusion model.
>>Response: Thank you.

>10) I remain concerned about the authors modeling. While I am not an expert on modeling, I wonder (as stated in #3) if the profound effects for the -3T aAUG with -3A/G dAUG constructs are driving the whole competitive phenomenon. If this is true, I don’t know how this would impact the modeling and whether it is appropriate to derive a model driven by only a small subset of the data.
>>Response: In fact, the inhibitory effects for the -3T aAUG with -3A/G dAUG constructs were “diluted” by other sequence contexts (which can be observed by comparing Figure 1E vs. the top right panel of Figure S2D). Please note that in our simulation, we considered all variants instead of only a subset of variants with T at the -3 position of the aAUG and A/G at the -3 position of the dAUG. The parameters of leakage rates for initiation codons in various sequence contexts are provided in Figure 5G and Figure S11D. The similarity between observed and simulated results for the -3A aAUG and -3T dAUG constructs using these parameters are now provided in the revised manuscript, in the wild-type or upf1 deletion background (Figure S11E-F).

>11) Lines 567-570: I think there is a possible flaw in this analysis or I misinterpreted the authors’ argument. Proximal dAUG will trigger NMD and these mRNAs will – due to the NMD – be underrepresented among the highly expressed genes. Thus, we expect to see fewer dAUGs in the most abundant mRNAs. Thus, the lack of dAUGs in highly expressed genes might not be due to selection against high level production of toxic peptides, but simply due to NMD.
>>Response: We thank Reviewer #2 for this valuable comment. The presence/absence of proximal out-of-frame dATG could at most make a two-fold difference in the mRNA level (Figure 3B and Figure S5B), and therefore, the observed mRNA level variation is mainly determined by other contributing factors such as transcription activity. During revision, we performed an additional analysis by dividing genes into two groups according to their transcription rates (which will not be affected by the presence/absence of dATGs) and our conclusions remained. The new results have been added to the revised manuscript (Figure S14B).

>12) Lines 583-4: “likely generally drove” seems too strong
>>Response: edited to “probably generally drove” during revision.

>13) The data in Figure 6F in not interpretable without RNA levels. As stated above, we know that NMD is amplifying effects with these reporters. As this paper is trying to investigate translation, it is important to remove the effects of mRNA turnover to reveal the true translation effects.
>>Response: Data for mRNA level are added to Figure 6F of the revised manuscript. Luciferase data are also updated to avoid incompatibility caused by batch effect (between luciferase assay and quantitative PCR) due to independent transfection between experiments.

>14) Six times in the Discussion and Conclusion, the authors use the word “massive”. I feel that this word is not justified. The authors performed a screen, I would not describe it as massive.
>>Response: We performed FACS-seq to quantify protein levels for thousands or tens of thousands of variants simultaneously. We can confirm that FACS-seq is one of the massively parallel assays (DOI: 10.1146/annurev-genom-083118-014845). We designed the library with the aim of testing the effects of dATGs, so the term “screen” is probably not adequate here.

>15) Lines 644-646: the insertion of a second inframe AUG increased GFP levels. What was the impact on mRNA levels?
>>Response: As shown in Figure 3B the mRNA levels were largely unchanged upon the insertion of a second in-frame AUG.

>16) Lines 652-663: see point #2 above. In this paragraph the authors interpret a 50% reduction in GFP as indicating a 50% change in utilization of the aAUG versus dAUG. However, the 50% reduction in GFP includes both the translation change and NMD. If the authors examine the data in Fig. S6B, the impacts of the dAUGs are less than the values stated in this paragraph. Again, it is important to focus on translation effects and not combined translation and NMD effects. This same criticism applies to the following paragraph (lines 665-677).
>>Response: We thank Reviewer #2 for this insightful comment. The numbers in both paragraphs have been updated with the corresponding results in the upf1 mutants during revision. The conclusions remained qualitatively unchanged.

>17) Line 802: “promotes” is not the correct word. The presence of an impediment to 5’-3’ motion does not promote 3’-5’ motion, it just blocks forward motion.
>>Response: edited to “elevate the probability of” during revision.

>18) Finally, I would like to comment on the authors’ rebuttal to reviewer #2, question 17 regarding the recently reported faster scanning rate for eukaryotic translation. From my understanding, it is best to focus on Fig. 2C which clearly shows the time gap between ribosome binding to an mRNA and large subunit association. This time gap, the time of scanning, is quantified in Figure S4C and shows a correlation with the length of the 5’ leader. While it could be argued that the ribosomes in this reconstituted system diffuse at a faster rate down the mRNA due to the lack of some factors that are present in vivo or perhaps do not flip directions as often as in vivo, an alternative possibility is that false assumptions in the authors’ modeling have resulted in overestimating the number of times the ribosome samples each nucleotide as it scans down the mRNA.
Finally, I would point out that the data in the bioRxiv paper reveals that while scanning is fast (100 nt/sec), the post-scanning delay of the ribosome at the start codon before subunit joining is slow (and rate-limiting). Thus, the published 10 nt/sec scanning times, which rely on the appearance of a reporter protein and thus do not directly measure scanning, are dominated by this slow rate of subunit joining and not a slow rate of scanning. This is an important distinction with direct implications on the authors’ proposed rate of scanning (with backtracking).
>>Response:
Two previously published papers measured the scanning rates as 6–10 nucleotides per second (Berthelot et al., 2004; Vassilenko et al., 2011). Note that they also generated variants with different 5’-UTR lengths and estimated the scanning rate from linear regression (an experimental strategy similar to that used in the bioRxiv paper mentioned by Reviewer #2).
Quoting from Berthelot et al.: “By considering the differences in delay times for the mRNAs with different length 5’ UTRs, we could eliminate the contributions of the early, undefined phase of the interactions between the mRNAs and the cell-free translation system that precedes binding of scanning-competent 40S subunits.”
Quoting from Vassilenko et al.: “Figure 4 shows the dependence of full-translation time on 5’-UTR length as determined for all 11 mRNAs in the wheat germ (Figure 4A) and Krebs-2 (Figure 4B) cell-free translation systems (see Supplementary Table S1 for numerical values). Remarkably, for all the cases analyzed, the data show that the full-translation time displayed positive correlation with 5’-UTR length and that the relationship was close to linear.”
We thank Reviewer #2 for providing an explanation of why the scanning rate estimated in this bioRxiv paper was ~10 times faster than the two previous studies; that is, their ribosomes in the reconstituted system is probably different from the transitional environment that is present in vivo. The two previous studies used cell lysates instead, which probably better reflects the full components of the cellular translation machinery. We mentioned this possibility in the revised manuscript and discussed how this system would help understand the scanning process.

>Reviewer #3: The new experiments and analyses and careful wording to describe their findings addressed the concerns of this reviewer. This reviewer recommends this manuscript for publication.
>>Response: We thank Reviewer #3 for recommending our work.
